# Supplementary material for: Pathophysiology of Cerebellar Degeneration in Mitochondrial Disorders: Insights from the Harlequin Mouse
Source: Int J Mol Sci. 2023 Jun 30;24(13):10973. doi: 10.3390/ijms241310973 (PMC10341771; doi:10.3390/ijms241310973)
Supplement: Supplementary file 1 [file ijms-24-10973-s001.zip › Amino acids 2m cerebellum/20201029_001Hq.53 Cbl_Method Report.pdf]

Biochrom 30+ Final Test

Method: C:\Biochrom\OpenLAB Projects\Default\Method\20180828mod.met
Standard: C:\Biochrom\OpenLAB Projects\Default\Result\20201029_001Hq.53 Cbl.dat
Date : 11/5/2020 1:29:55 AM (GMT +01:00)

Instrument Serial No : 133260
Column No : H-0795
Resin No : 132-56

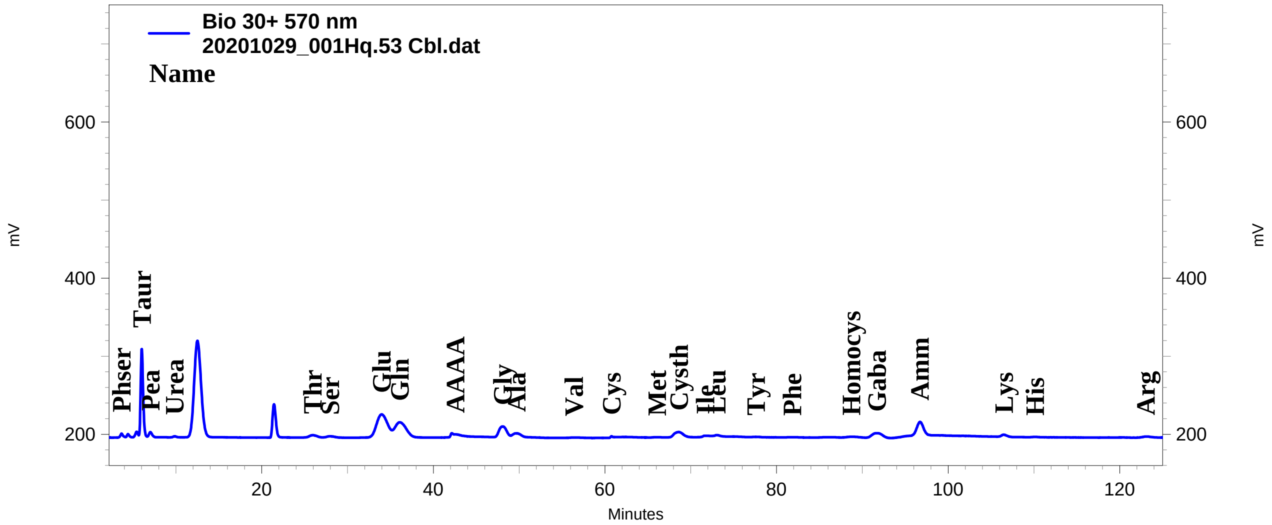

Bio 30+ 570 nm
Results

Pk #	Name	Retention Time	Area	ESTD concentration	Units
1	Phser	3.667	10652134	7.411	µmol/L
4	Taur	6.033	227367197	200.924	µmol/L
5	Pea	7.033	18209591	22.029	µmol/L
6	Urea	9.833	3472946	91.159	µmol/L
	Asp			0.000 BDL	µmol/L
9	Thr	25.933	17624468	13.730	µmol/L
10	Ser	27.967	10983081	8.454	µmol/L
	Asn			0.000 BDL	µmol/L
11	Glu	33.967	261756794	207.134	µmol/L
12	Gln	36.067	178769994	141.179	µmol/L
	Sarc			0.000 BDL	µmol/L
14	AAAA	42.567	20377488	16.990	µmol/L
15	Gly	48.067	82298658	59.786	µmol/L
16	Ala	49.700	33758488	26.691	µmol/L
	Citr			0.000 BDL	µmol/L
	Aaba			0.000 BDL	µmol/L
17	Val	56.400	5123322	4.233	µmol/L
18	Cys	60.767	2602045	1.768	µmol/L
19	Met	66.067	2371482	1.839	µmol/L
20	Cysth	68.633	48357014	35.008	µmol/L
21	Ile	71.700	10008737	7.926	µmol/L
22	Leu	73.000	9485081	7.103	µmol/L
	Nleu			0.000 BDL	µmol/L
23	Tyr	77.600	2501584	1.998	µmol/L
	B-ala			0.000 BDL	µmol/L
24	Phe	81.833	2530152	1.984	µmol/L
	Baiba			0.000 BDL	µmol/L
26	Homocys	88.700	10621120	4.247	µmol/L
27	Gaba	91.700	48943202	49.064	µmol/L
	Ethan			0.000 BDL	µmol/L
28	Amm	96.700	114439689	84.752	µmol/L
	Hylys			0.000 BDL	µmol/L
	Orn			0.000 BDL	µmol/L
29	Lys	106.500	11189749	8.255	µmol/L
	1-Mhis			0.000 BDL	µmol/L
30	His	110.133	2553006	1.805	µmol/L
	Trp			0.000 BDL	µmol/L
	3-Mhis			0.000 BDL	µmol/L
	Ans			0.000 BDL	µmol/L
	Car			0.000 BDL	µmol/L
31	Arg	123.033	8716694	7.043	µmol/L

Totals			1144713716	1012.514	

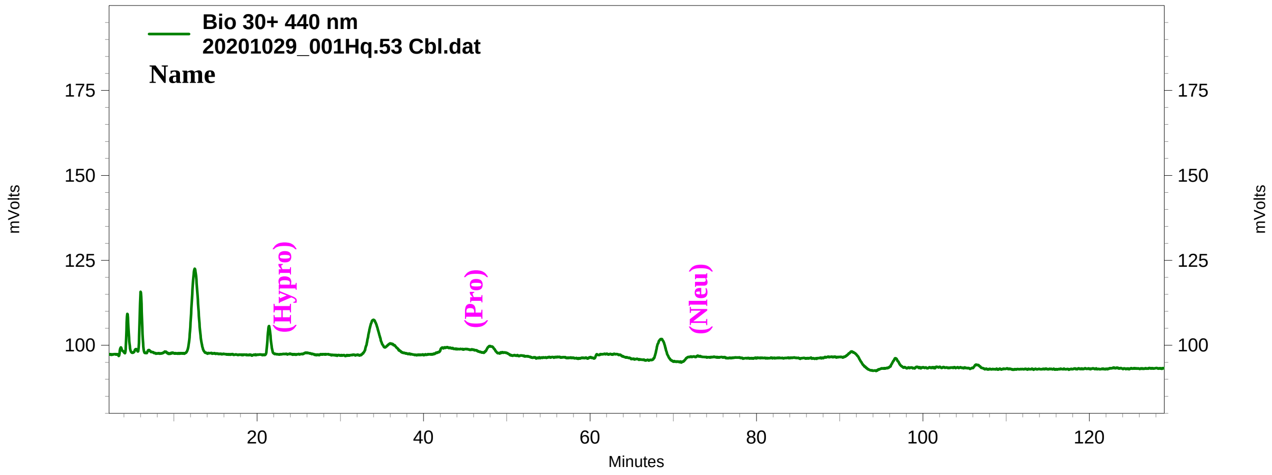

Bio 30+ 440 nm
Results

Pk #	Name	Retention Time	Area	ESTD concentration	Units
	Hypro			0.000 BDL	µmol/L
	Pro			0.000 BDL	µmol/L
	Nleu			0.000 BDL	µmol/L

Totals					
