# Supplementary material for: Pathophysiology of Cerebellar Degeneration in Mitochondrial Disorders: Insights from the Harlequin Mouse
Source: Int J Mol Sci. 2023 Jun 30;24(13):10973. doi: 10.3390/ijms241310973 (PMC10341771; doi:10.3390/ijms241310973)
Supplement: Supplementary file 1 [file ijms-24-10973-s001.zip › Amino acids 2m cerebellum/20201029_001Hq.55 Cbl_Method Report.pdf]

Biochrom 30+ Final Test

Method: C:\Biochrom\OpenLAB Projects\Default\Method\20180828mod.met
Standard: C:\Biochrom\OpenLAB Projects\Default\Result\20201029_001Hq.55 Cbl.dat
Date : 11/5/2020 1:32:01 AM (GMT +01:00)

Instrument Serial No : 133260
Column No : H-0795
Resin No : 132-56

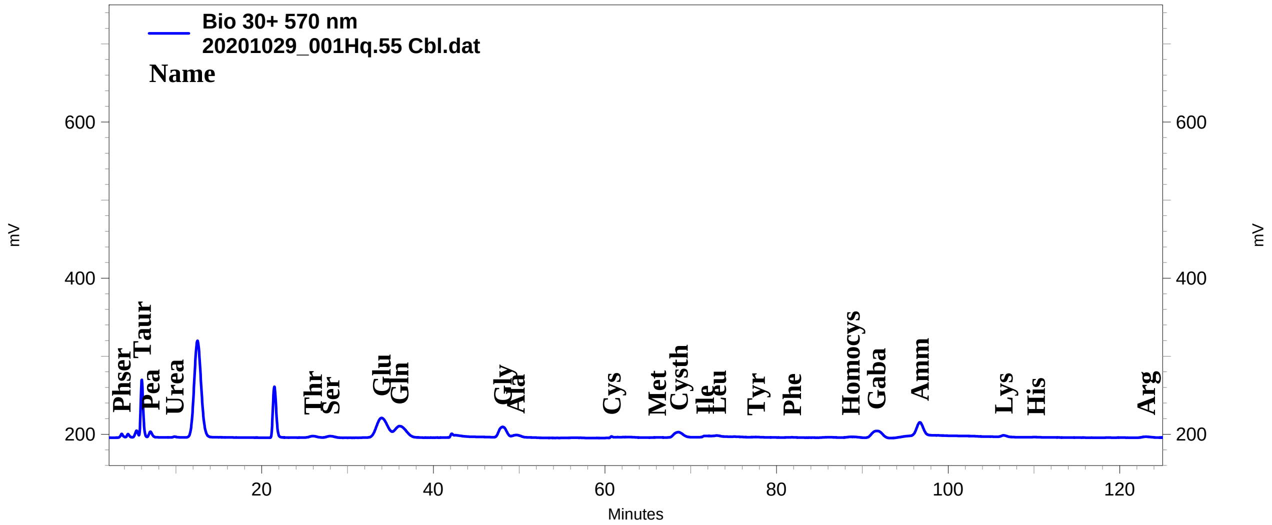

Bio 30+ 570 nm
Results

Pk #	Name	Retention Time	Area	ESTD concentration	Units
1	Phser	3.667	9849881	6.853	µmol/L
4	Taur	6.033	148925024	131.605	µmol/L
5	Pea	7.033	20260571	24.510	µmol/L
6	Urea	9.833	2651268	69.592	µmol/L
	Asp			0.000 BDL	µmol/L
9	Thr	25.967	10877170	8.474	µmol/L
10	Ser	28.000	12203820	9.393	µmol/L
	Asn			0.000 BDL	µmol/L
11	Glu	33.967	223261486	176.672	µmol/L
12	Gln	36.033	135433096	106.955	µmol/L
	Sarc			0.000 BDL	µmol/L
	AAAA			0.000 BDL	µmol/L
14	Gly	48.100	80527699	58.499	µmol/L
15	Ala	49.600	19804361	15.658	µmol/L
	Citr			0.000 BDL	µmol/L
	Aaba			0.000 BDL	µmol/L
	Val			0.000 BDL	µmol/L
16	Cys	60.767	2567455	1.745	µmol/L
17	Met	66.067	1809365	1.403	µmol/L
18	Cysth	68.600	47430606	34.338	µmol/L
19	Ile	71.667	8385743	6.641	µmol/L
20	Leu	73.067	7142798	5.349	µmol/L
	Nleu			0.000 BDL	µmol/L
21	Tyr	77.600	2572346	2.055	µmol/L
	B-ala			0.000 BDL	µmol/L
22	Phe	81.800	2692842	2.111	µmol/L
	Baiba			0.000 BDL	µmol/L
23	Homocys	88.633	8238523	3.295	µmol/L
24	Gaba	91.633	73638305	73.820	µmol/L
	Ethan			0.000 BDL	µmol/L
25	Amm	96.700	112590476	83.383	µmol/L
	Hylys			0.000 BDL	µmol/L
	Orn			0.000 BDL	µmol/L
26	Lys	106.467	7650292	5.644	µmol/L
	1-Mhis			0.000 BDL	µmol/L
27	His	110.233	2192131	1.550	µmol/L
	Trp			0.000 BDL	µmol/L
	3-Mhis			0.000 BDL	µmol/L
	Ans			0.000 BDL	µmol/L
	Car			0.000 BDL	µmol/L
28	Arg	123.067	7824157	6.322	µmol/L
Totals			948529415	835.865	

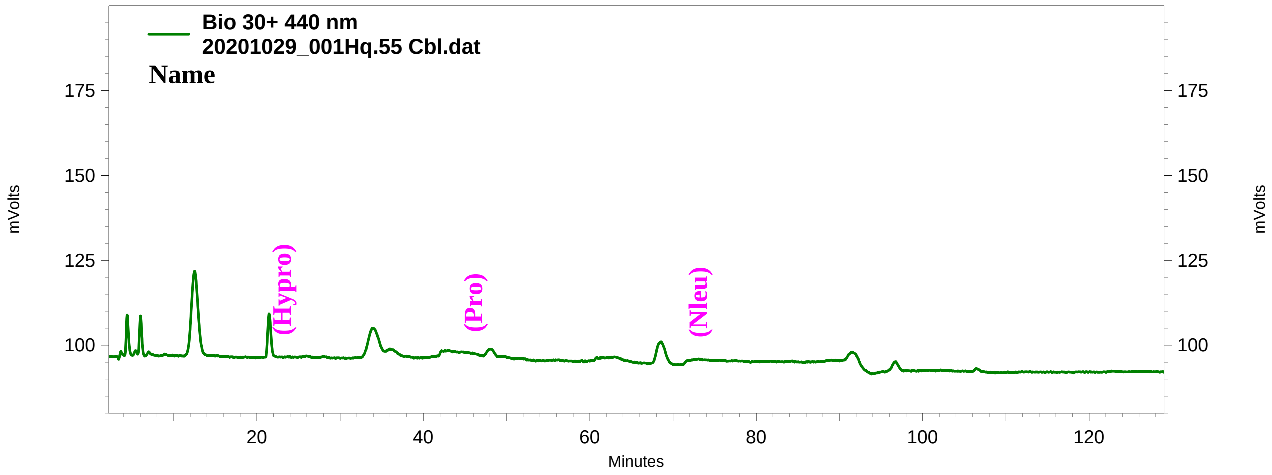

Bio 30+ 440 nm
Results

Pk #	Name	Retention Time	Area	ESTD concentration	Units
	Hypro			0.000 BDL	µmol/L
	Pro			0.000 BDL	µmol/L
	Nleu			0.000 BDL	µmol/L

Totals					
