# Supplementary material for: Pathophysiology of Cerebellar Degeneration in Mitochondrial Disorders: Insights from the Harlequin Mouse
Source: Int J Mol Sci. 2023 Jun 30;24(13):10973. doi: 10.3390/ijms241310973 (PMC10341771; doi:10.3390/ijms241310973)
Supplement: Supplementary file 1 [file ijms-24-10973-s001.zip › Amino acids 2m cerebellum/20201029_001Hq.57 Cbl_Method Report.pdf]

Biochrom 30+ Final Test

Method: C:\Biochrom\OpenLAB Projects\Default\Method\20180828mod.met
Standard: C:\Biochrom\OpenLAB Projects\Default\Result\20201029_001Hq.57 Cbl.dat
Date : 11/5/2020 1:37:00 AM (GMT +01:00)

Instrument Serial No : 133260
Column No : H-0795
Resin No : 132-56

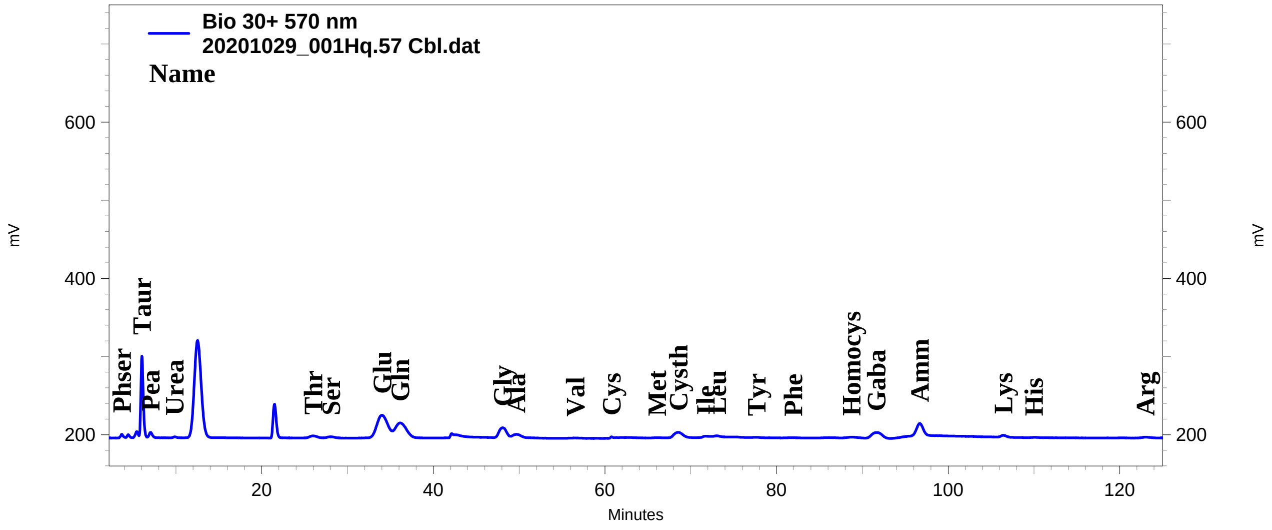

Bio 30+ 570 nm
Results

Pk #	Name	Retention Time	Area	ESTD concentration	Units
1	Phser	3.700	9997834	6.956	µmol/L
4	Taur	6.033	207515915	183.382	µmol/L
5	Pea	7.033	18839064	22.791	µmol/L
6	Urea	9.833	3354988	88.063	µmol/L
	Asp			0.000 BDL	µmol/L
9	Thr	26.000	14961797	11.656	µmol/L
10	Ser	28.067	9583607	7.377	µmol/L
	Asn			0.000 BDL	µmol/L
11	Glu	34.033	255683566	202.328	µmol/L
12	Gln	36.133	174663762	137.936	µmol/L
	Sarc			0.000 BDL	µmol/L
	AAAA			0.000 BDL	µmol/L
14	Gly	48.067	74955753	54.452	µmol/L
15	Ala	49.667	28246733	22.333	µmol/L
	Citr			0.000 BDL	µmol/L
	Aaba			0.000 BDL	µmol/L
16	Val	56.567	3692423	3.051	µmol/L
17	Cys	60.767	2839337	1.930	µmol/L
18	Met	66.067	2095670	1.625	µmol/L
19	Cysth	68.567	48139854	34.851	µmol/L
20	Ile	71.733	9399154	7.443	µmol/L
21	Leu	73.067	7315983	5.479	µmol/L
	Nleu			0.000 BDL	µmol/L
22	Tyr	77.667	2803776	2.239	µmol/L
	B-ala			0.000 BDL	µmol/L
23	Phe	81.933	2902834	2.276	µmol/L
	Baiba			0.000 BDL	µmol/L
24	Homocys	88.733	8500428	3.399	µmol/L
25	Gaba	91.633	61214572	61.366	µmol/L
	Ethan			0.000 BDL	µmol/L
26	Amm	96.700	104848322	77.649	µmol/L
	Hylys			0.000 BDL	µmol/L
	Orn			0.000 BDL	µmol/L
27	Lys	106.433	9259092	6.831	µmol/L
	1-Mhis			0.000 BDL	µmol/L
28	His	110.000	2698833	1.908	µmol/L
	Trp			0.000 BDL	µmol/L
	3-Mhis			0.000 BDL	µmol/L
	Ans			0.000 BDL	µmol/L
	Car			0.000 BDL	µmol/L
29	Arg	123.000	5881172	4.752	µmol/L

Totals			1069394469	952.072	

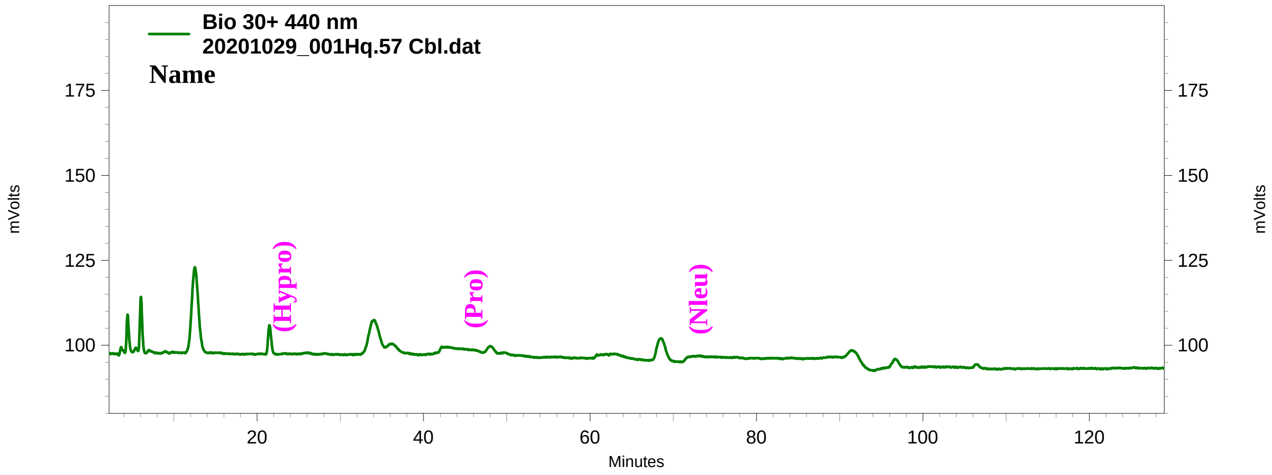

Bio 30+ 440 nm
Results

Pk #	Name	Retention Time	Area	ESTD concentration	Units
	Hypro			0.000 BDL	µmol/L
	Pro			0.000 BDL	µmol/L
	Nleu			0.000 BDL	µmol/L

Totals					
