# Supplementary material for: Pathophysiology of Cerebellar Degeneration in Mitochondrial Disorders: Insights from the Harlequin Mouse
Source: Int J Mol Sci. 2023 Jun 30;24(13):10973. doi: 10.3390/ijms241310973 (PMC10341771; doi:10.3390/ijms241310973)
Supplement: Supplementary file 1 [file ijms-24-10973-s001.zip › Amino acids 2m cerebellum/20201029_001Hq56 Cbl_Method Report.pdf]

Biochrom 30+ Final Test

Method: C:\Biochrom\OpenLAB Projects\Default\Method\20180828mod.met
Standard: C:\Biochrom\OpenLAB Projects\Default\Result\20201029_001Hq56 Cbl.dat
Date : 11/5/2020 1:35:51 AM (GMT +01:00)

Instrument Serial No : 133260
Column No : H-0795
Resin No : 132-56

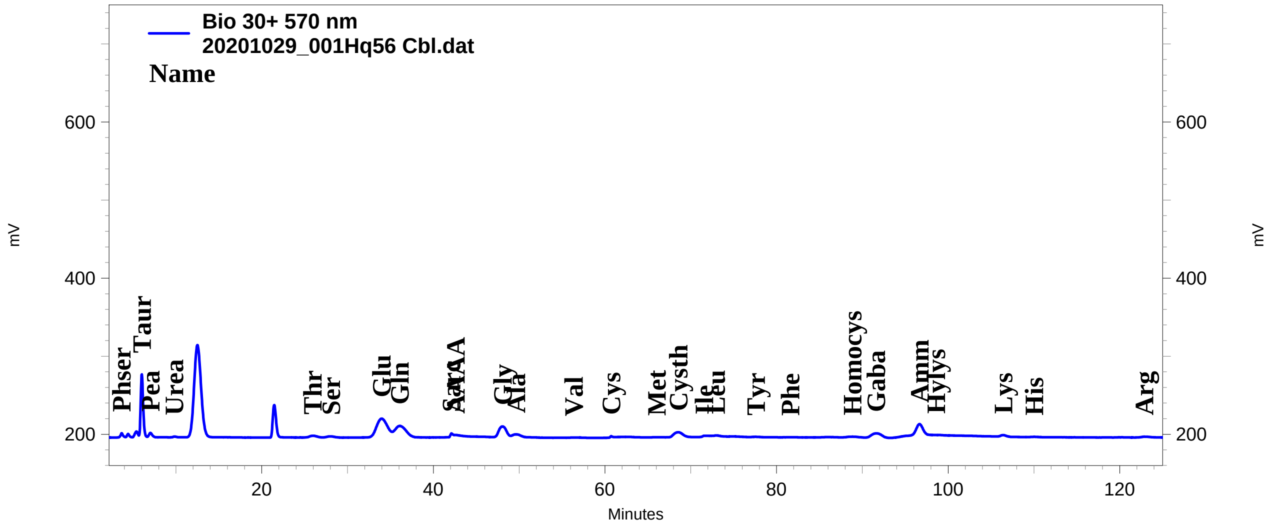

Bio 30+ 570 nm
Results

Pk #	Name	Retention Time	Area	ESTD concentration	Units
1	Phser	3.667	10373507	7.217	µmol/L
4	Taur	6.033	161511806	142.728	µmol/L
5	Pea	7.033	15813445	19.130	µmol/L
6	Urea	9.800	2660242	69.827	µmol/L
	Asp			0.000 BDL	µmol/L
9	Thr	25.933	12411294	9.669	µmol/L
10	Ser	28.067	9205760	7.086	µmol/L
	Asn			0.000 BDL	µmol/L
11	Glu	33.967	210307515	166.421	µmol/L
12	Gln	36.067	135433926	106.955	µmol/L
13	Sarc	42.100	11490864	71.707	µmol/L
14	AAAA	42.600	13927754	11.613	µmol/L
15	Gly	48.100	82690621	60.071	µmol/L
16	Ala	49.667	23933818	18.923	µmol/L
	Citr			0.000 BDL	µmol/L
	Aaba			0.000 BDL	µmol/L
17	Val	56.367	2350886	1.943	µmol/L
18	Cys	60.733	2501466	1.700	µmol/L
19	Met	66.033	1941152	1.505	µmol/L
20	Cysth	68.567	45581799	32.999	µmol/L
21	Ile	71.567	9719376	7.697	µmol/L
22	Leu	72.933	7161176	5.363	µmol/L
	Nleu			0.000 BDL	µmol/L
23	Tyr	77.600	2635260	2.105	µmol/L
	B-ala			0.000 BDL	µmol/L
24	Phe	81.600	2330900	1.827	µmol/L
	Baiba			0.000 BDL	µmol/L
25	Homocys	88.833	9857113	3.942	µmol/L
26	Gaba	91.600	43789116	43.897	µmol/L
	Ethan			0.000 BDL	µmol/L
27	Amm	96.667	113607982	84.136	µmol/L
28	Hylys	98.600	3348982	2.600	µmol/L
	Orn			0.000 BDL	µmol/L
29	Lys	106.433	7996593	5.899	µmol/L
	1-Mhis			0.000 BDL	µmol/L
30	His	110.033	2454268	1.735	µmol/L
	Trp			0.000 BDL	µmol/L
	3-Mhis			0.000 BDL	µmol/L
	Ans			0.000 BDL	µmol/L
	Car			0.000 BDL	µmol/L
31	Arg	122.867	7674372	6.201	µmol/L

Totals			952710993	894.896	

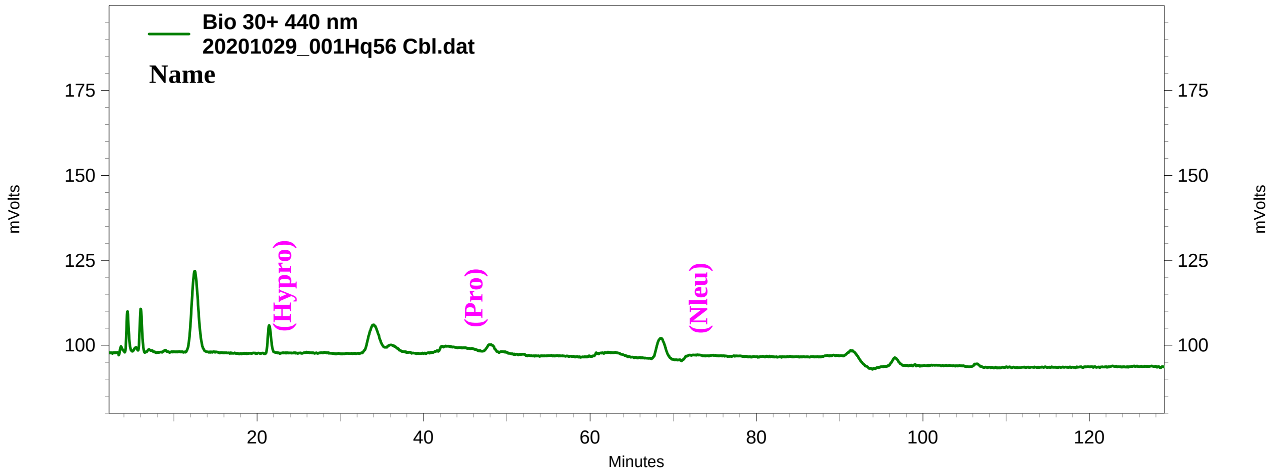

Bio 30+ 440 nm
Results

Pk #	Name	Retention Time	Area	ESTD concentration	Units
	Hypro			0.000 BDL	µmol/L
	Pro			0.000 BDL	µmol/L
	Nleu			0.000 BDL	µmol/L

Totals					
