# Supplementary material for: Pathophysiology of Cerebellar Degeneration in Mitochondrial Disorders: Insights from the Harlequin Mouse
Source: Int J Mol Sci. 2023 Jun 30;24(13):10973. doi: 10.3390/ijms241310973 (PMC10341771; doi:10.3390/ijms241310973)
Supplement: Supplementary file 1 [file ijms-24-10973-s001.zip › Amino acids 2m cerebellum/20201029_001WT1 Cbl_Method Report.pdf]

Biochrom 30+ Final Test

Method: C:\Biochrom\OpenLAB Projects\Default\Method\20180828mod.met
Standard: C:\Biochrom\OpenLAB Projects\Default\Result\20201029_001WT1 Cbl.dat
Date : 11/5/2020 1:19:37 AM (GMT +01:00)

Instrument Serial No : 133260
Column No : H-0795
Resin No : 132-56

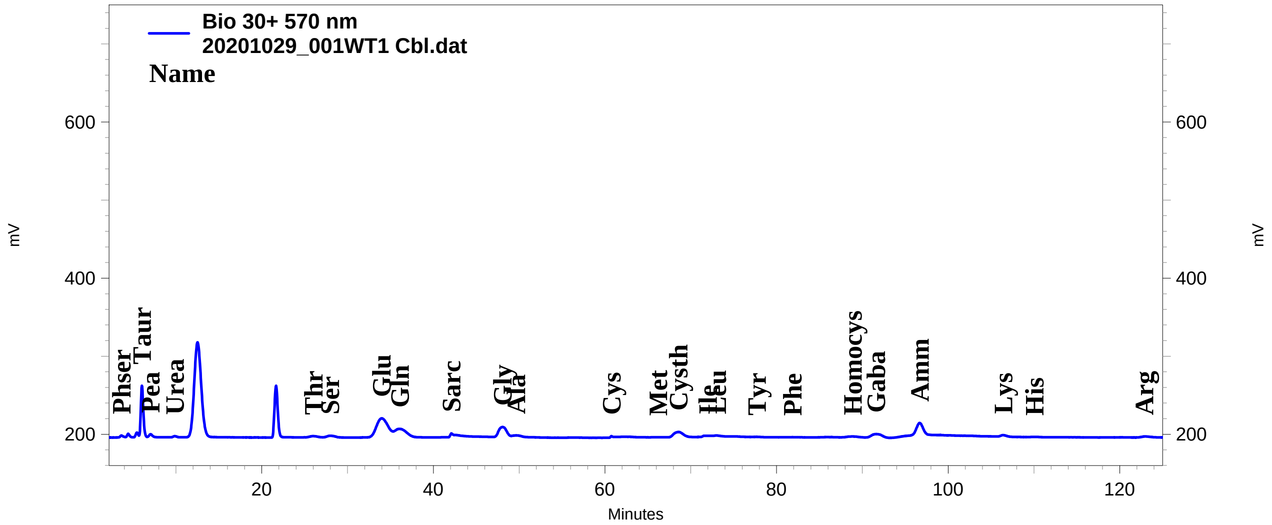

Bio 30+ 570 nm
Results

Pk #	Name	Retention Time	Area	ESTD concentration	Units
1	Phser	3.667	5830050	4.056	µmol/L
4	Taur	6.033	134768844	119.095	µmol/L
5	Pea	7.033	10970878	13.272	µmol/L
6	Urea	9.867	3923466	102.985	µmol/L
	Asp			0.000 BDL	µmol/L
9	Thr	26.033	9420213	7.339	µmol/L
10	Ser	27.967	14553155	11.202	µmol/L
	Asn			0.000 BDL	µmol/L
11	Glu	33.967	228275715	180.640	µmol/L
12	Gln	36.100	104082976	82.197	µmol/L
13	Sarc	42.100	7506176	46.841	µmol/L
	AAAA			0.000 BDL	µmol/L
14	Gly	48.067	81512059	59.214	µmol/L
15	Ala	49.667	14831067	11.726	µmol/L
	Citr			0.000 BDL	µmol/L
	Aaba			0.000 BDL	µmol/L
	Val			0.000 BDL	µmol/L
16	Cys	60.767	2554418	1.736	µmol/L
17	Met	66.200	1043406	0.809	µmol/L
18	Cysth	68.567	49064471	35.520	µmol/L
19	Ile	72.000	7657263	6.064	µmol/L
20	Leu	73.067	5156302	3.861	µmol/L
	Nleu			0.000 BDL	µmol/L
21	Tyr	77.700	1552400	1.240	µmol/L
	B-ala			0.000 BDL	µmol/L
22	Phe	81.867	2350208	1.843	µmol/L
	Baiba			0.000 BDL	µmol/L
23	Homocys	88.867	11350900	4.539	µmol/L
24	Gaba	91.600	38768058	38.864	µmol/L
	Ethan			0.000 BDL	µmol/L
25	Amm	96.700	105781247	78.340	µmol/L
	Hylys			0.000 BDL	µmol/L
	Orn			0.000 BDL	µmol/L
26	Lys	106.433	7880350	5.814	µmol/L
	1-Mhis			0.000 BDL	µmol/L
27	His	110.067	1845575	1.305	µmol/L
	Trp			0.000 BDL	µmol/L
	3-Mhis			0.000 BDL	µmol/L
	Ans			0.000 BDL	µmol/L
	Car			0.000 BDL	µmol/L
28	Arg	122.867	8238230	6.656	µmol/L
Totals			858917427	825.158	

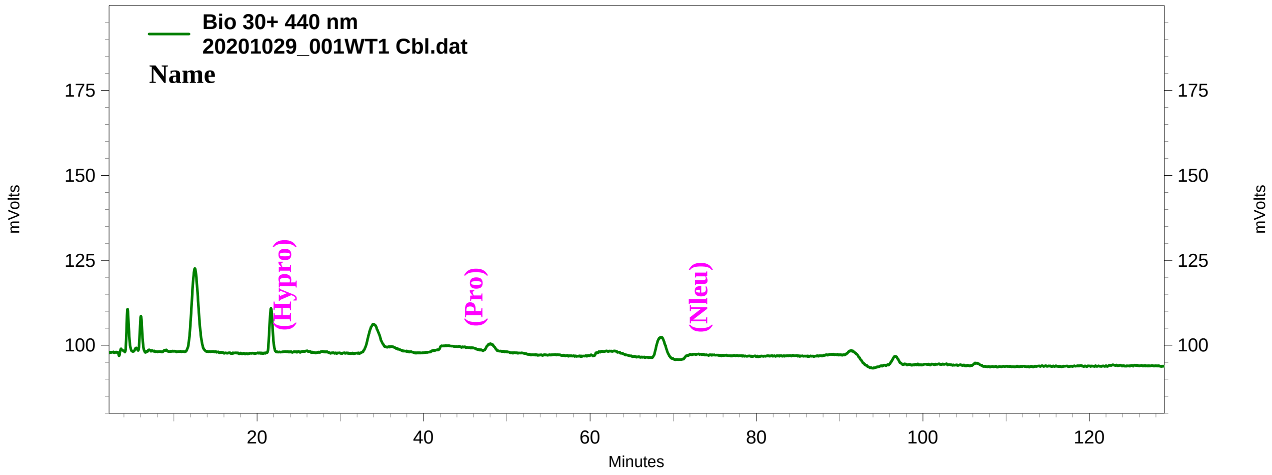

Bio 30+ 440 nm
Results

Pk #	Name	Retention Time	Area	ESTD concentration	Units
	Hypro			0.000 BDL	µmol/L
	Pro			0.000 BDL	µmol/L
	Nleu			0.000 BDL	µmol/L

Totals					
