# Supplementary material for: Pathophysiology of Cerebellar Degeneration in Mitochondrial Disorders: Insights from the Harlequin Mouse
Source: Int J Mol Sci. 2023 Jun 30;24(13):10973. doi: 10.3390/ijms241310973 (PMC10341771; doi:10.3390/ijms241310973)
Supplement: Supplementary file 1 [file ijms-24-10973-s001.zip › Amino acids 2m cerebellum/20201029_001WT11 Cbl_Method Report.pdf]

Biochrom 30+ Final Test

Method: C:\Biochrom\OpenLAB Projects\Default\Method\20180828mod.met
Standard: C:\Biochrom\OpenLAB Projects\Default\Result\20201029_001WT11 Cbl.dat
Date : 11/5/2020 1:22:55 AM (GMT +01:00)

Instrument Serial No : 133260
Column No : H-0795
Resin No : 132-56

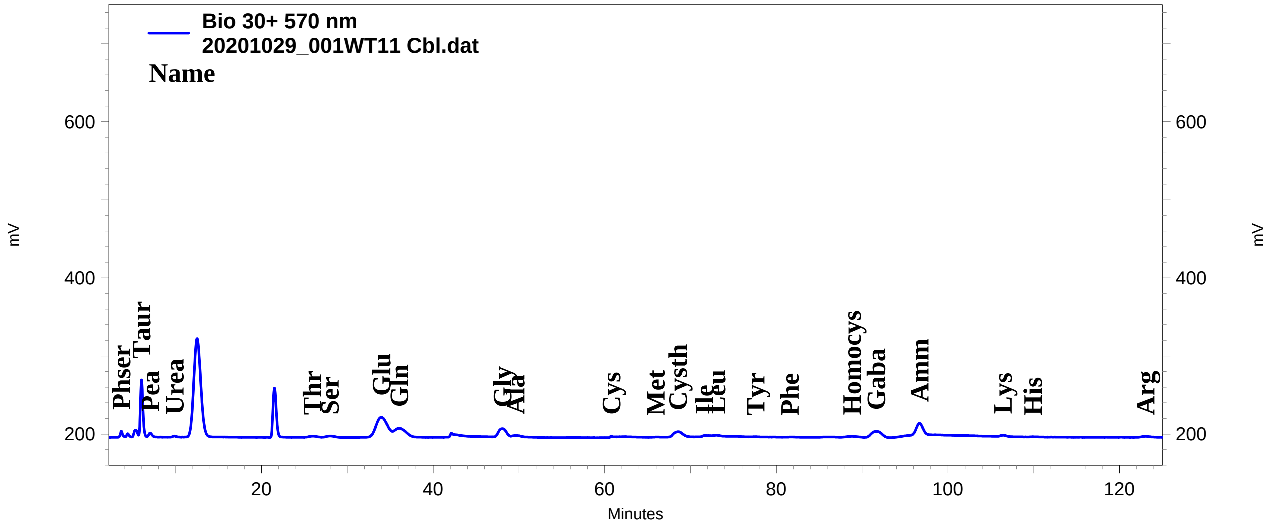

Bio 30+ 570 nm
Results

Pk #	Name	Retention Time	Area	ESTD concentration	Units
1	Phser	3.667	14127921	9.829	µmol/L
4	Taur	6.000	150530629	133.024	µmol/L
5	Pea	7.033	15037747	18.192	µmol/L
6	Urea	9.867	3689267	96.837	µmol/L
	Asp			0.000 BDL	µmol/L
9	Thr	25.900	8098206	6.309	µmol/L
10	Ser	27.933	11382614	8.761	µmol/L
	Asn			0.000 BDL	µmol/L
11	Glu	33.967	237056881	187.588	µmol/L
12	Gln	36.033	107599112	84.973	µmol/L
	Sarc			0.000 BDL	µmol/L
	AAAA			0.000 BDL	µmol/L
14	Gly	48.067	65138183	47.320	µmol/L
15	Ala	49.600	12280581	9.710	µmol/L
	Citr			0.000 BDL	µmol/L
	Aaba			0.000 BDL	µmol/L
	Val			0.000 BDL	µmol/L
16	Cys	60.767	2704058	1.838	µmol/L
17	Met	65.933	1225474	0.950	µmol/L
18	Cysth	68.533	49082128	35.533	µmol/L
19	Ile	71.600	8460520	6.700	µmol/L
20	Leu	73.000	5441134	4.075	µmol/L
	Nleu			0.000 BDL	µmol/L
21	Tyr	77.567	2061160	1.646	µmol/L
	B-ala			0.000 BDL	µmol/L
22	Phe	81.567	2130218	1.670	µmol/L
	Baiba			0.000 BDL	µmol/L
23	Homocys	88.800	11639309	4.654	µmol/L
24	Gaba	91.633	66037450	66.201	µmol/L
	Ethan			0.000 BDL	µmol/L
25	Amm	96.700	106000641	78.502	µmol/L
	Hylys			0.000 BDL	µmol/L
	Orn			0.000 BDL	µmol/L
26	Lys	106.400	6304970	4.651	µmol/L
	1-Mhis			0.000 BDL	µmol/L
27	His	109.900	2683282	1.897	µmol/L
	Trp			0.000 BDL	µmol/L
	3-Mhis			0.000 BDL	µmol/L
	Ans			0.000 BDL	µmol/L
	Car			0.000 BDL	µmol/L
28	Arg	123.033	7889542	6.375	µmol/L

Totals			896601027	817.237	

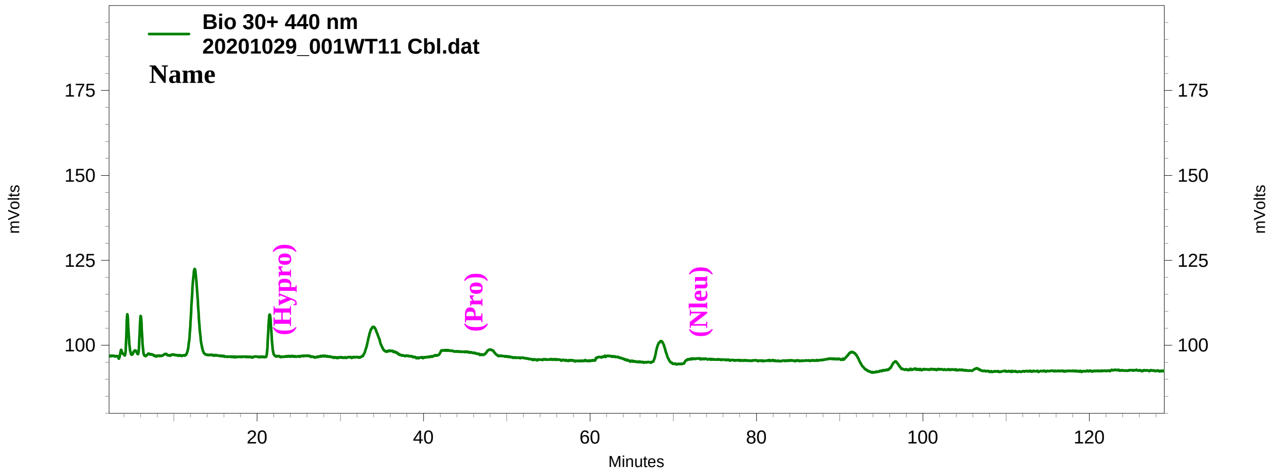

Bio 30+ 440 nm
Results

Pk #	Name	Retention Time	Area	ESTD concentration	Units
	Hypro			0.000 BDL	µmol/L
	Pro			0.000 BDL	µmol/L
	Nleu			0.000 BDL	µmol/L

Totals					
