# Supplementary material for: Pathophysiology of Cerebellar Degeneration in Mitochondrial Disorders: Insights from the Harlequin Mouse
Source: Int J Mol Sci. 2023 Jun 30;24(13):10973. doi: 10.3390/ijms241310973 (PMC10341771; doi:10.3390/ijms241310973)
Supplement: Supplementary file 1 [file ijms-24-10973-s001.zip › Amino acids 2m cerebellum/20201029_001WT12 Cbl_Method Report.pdf]

Biochrom 30+ Final Test

Method: C:\Biochrom\OpenLAB Projects\Default\Method\20180828mod.met
Standard: C:\Biochrom\OpenLAB Projects\Default\Result\20201029_001WT12 Cbl.dat
Date : 11/5/2020 1:24:25 AM (GMT +01:00)

Instrument Serial No : 133260
Column No : H-0795
Resin No : 132-56

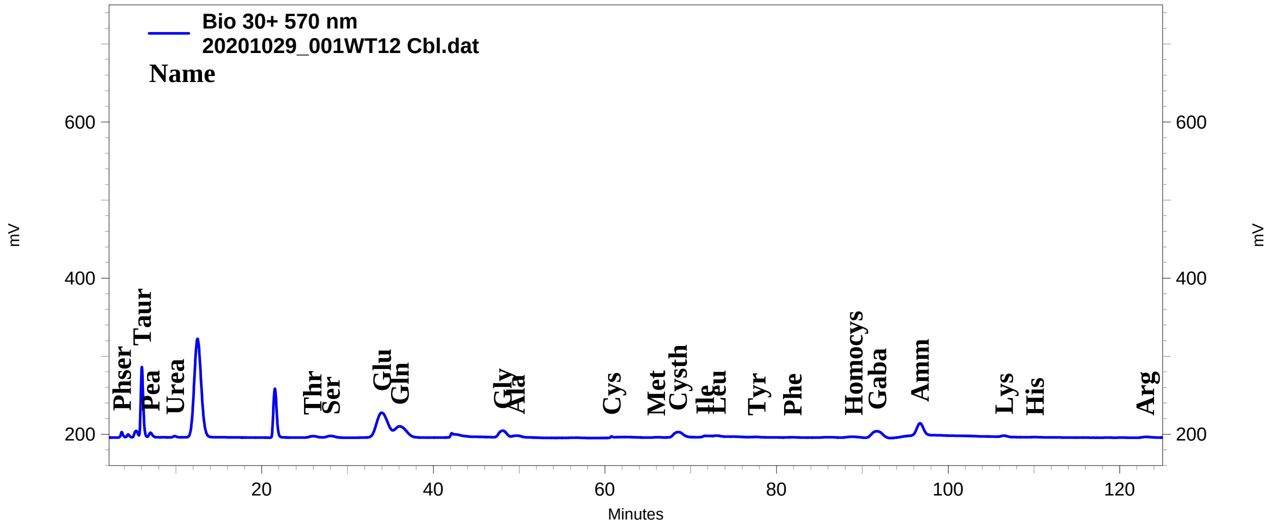

Bio 30+ 570 nm
Results

Pk #	Name	Retention Time	Area	ESTD concentration	Units
1	Phser	3.700	12968647	9.023	µmol/L
4	Taur	6.033	181399333	160.303	µmol/L
5	Pea	7.033	17097519	20.684	µmol/L
6	Urea	9.900	4270176	112.085	µmol/L
	Asp			0.000 BDL	µmol/L
9	Thr	25.900	10381682	8.088	µmol/L
10	Ser	28.033	14491907	11.155	µmol/L
	Asn			0.000 BDL	µmol/L
11	Glu	34.000	289739550	229.277	µmol/L
12	Gln	36.067	132795280	104.871	µmol/L
	Sarc			0.000 BDL	µmol/L
	AAAA			0.000 BDL	µmol/L
14	Gly	48.067	50867130	36.952	µmol/L
15	Ala	49.600	15147584	11.976	µmol/L
	Citr			0.000 BDL	µmol/L
	Aaba			0.000 BDL	µmol/L
	Val			0.000 BDL	µmol/L
16	Cys	60.767	2243536	1.525	µmol/L
17	Met	65.967	1648558	1.278	µmol/L
18	Cysth	68.500	49467076	35.812	µmol/L
19	Ile	71.667	9325700	7.385	µmol/L
20	Leu	73.033	6555272	4.909	µmol/L
	Nleu			0.000 BDL	µmol/L
21	Tyr	77.667	2933346	2.343	µmol/L
	B-ala			0.000 BDL	µmol/L
22	Phe	81.867	2750654	2.156	µmol/L
	Baiba			0.000 BDL	µmol/L
23	Homocys	88.967	11351247	4.539	µmol/L
24	Gaba	91.733	70643418	70.818	µmol/L
	Ethan			0.000 BDL	µmol/L
25	Amm	96.700	104561696	77.437	µmol/L
	Hylys			0.000 BDL	µmol/L
	Orn			0.000 BDL	µmol/L
26	Lys	106.500	5912440	4.362	µmol/L
	1-Mhis			0.000 BDL	µmol/L
27	His	110.100	2138233	1.511	µmol/L
	Trp			0.000 BDL	µmol/L
	3-Mhis			0.000 BDL	µmol/L
	Ans			0.000 BDL	µmol/L
	Car			0.000 BDL	µmol/L
28	Arg	122.967	6108480	4.935	µmol/L

Totals			1004798464	923.426	

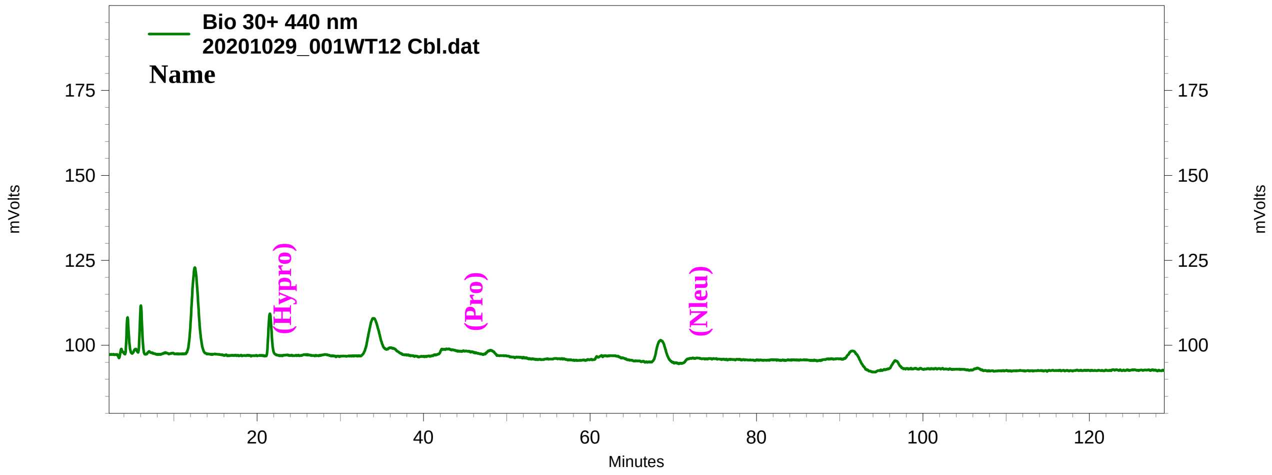

Bio 30+ 440 nm
Results

Pk #	Name	Retention Time	Area	ESTD concentration	Units
	Hypro			0.000 BDL	µmol/L
	Pro			0.000 BDL	µmol/L
	Nleu			0.000 BDL	µmol/L

Totals					
