# Supplementary material for: Pathophysiology of Cerebellar Degeneration in Mitochondrial Disorders: Insights from the Harlequin Mouse
Source: Int J Mol Sci. 2023 Jun 30;24(13):10973. doi: 10.3390/ijms241310973 (PMC10341771; doi:10.3390/ijms241310973)
Supplement: Supplementary file 1 [file ijms-24-10973-s001.zip › Amino acids 2m cerebellum/20201029_001WT3 Cbl_Method Report.pdf]

## Biochrom 30+ Final Test

Method: C:\Biochrom\OpenLAB Projects\Default\Method\20180828mod.met
Standard: C:\Biochrom\OpenLAB Projects\Default\Result\20201029_001WT3 Cbl.dat
Date : 11/5/2020 1:21:30 AM (GMT +01:00)

Instrument Serial No : 133260
Column No : H-0795
Resin No : 132-56

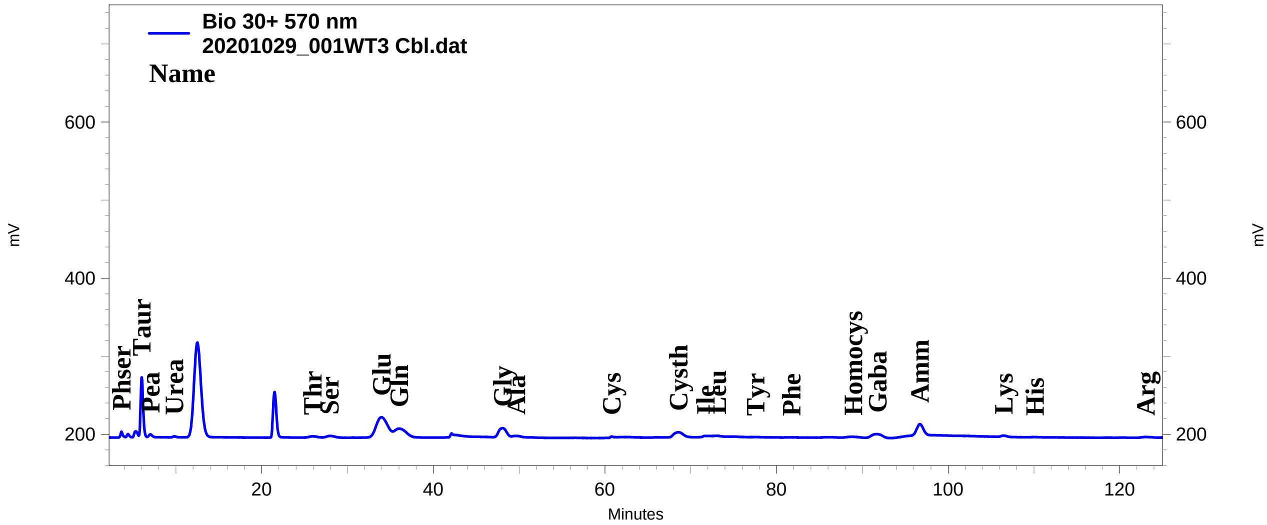


**Bio 30+ 570 nm**
**Results**

| Pk # | Name | Retention Time | Area | ESTD concentration | Units |
|---|---|---|---|---|---|
| 1 | Phser | 3.667 | 12863106 | 8.949 | µmol/L |
| 4 | Taur | 6.000 | 157432772 | 139.123 | µmol/L |
| 5 | Pea | 7.033 | 10538325 | 12.749 | µmol/L |
| 6 | Urea | 9.800 | 3559370 | 93.428 | µmol/L |
| | Asp | | | 0.000 BDL | µmol/L |
| 9 | Thr | 25.933 | 8724977 | 6.797 | µmol/L |
| 10 | Ser | 27.900 | 14281545 | 10.993 | µmol/L |
| | Asn | | | 0.000 BDL | µmol/L |
| 11 | Glu | 33.967 | 240515755 | 190.326 | µmol/L |
| 12 | Gln | 36.000 | 106471749 | 84.083 | µmol/L |
| | Sarc | | | 0.000 BDL | µmol/L |
| | AAAA | | | 0.000 BDL | µmol/L |
| 14 | Gly | 48.067 | 72757185 | 52.854 | µmol/L |
| 15 | Ala | 49.667 | 11276917 | 8.916 | µmol/L |
| | Citr | | | 0.000 BDL | µmol/L |
| | Aaba | | | 0.000 BDL | µmol/L |
| | Val | | | 0.000 BDL | µmol/L |
| 16 | Cys | 60.767 | 2237166 | 1.520 | µmol/L |
| | Met | | | 0.000 BDL | µmol/L |
| 17 | Cysth | 68.567 | 48194856 | 34.891 | µmol/L |
| 18 | Ile | 71.733 | 8710425 | 6.898 | µmol/L |
| 19 | Leu | 73.067 | 5897354 | 4.416 | µmol/L |
| | Nleu | | | 0.000 BDL | µmol/L |
| 20 | Tyr | 77.533 | 2071387 | 1.654 | µmol/L |
| | B-ala | | | 0.000 BDL | µmol/L |
| 21 | Phe | 81.733 | 1738723 | 1.363 | µmol/L |
| | Baiba | | | 0.000 BDL | µmol/L |
| 22 | Homocys | 88.933 | 11339942 | 4.535 | µmol/L |
| 23 | Gaba | 91.767 | 40282958 | 40.383 | µmol/L |
| | Ethan | | | 0.000 BDL | µmol/L |
| 24 | Amm | 96.700 | 99130664 | 73.414 | µmol/L |
| | Hylys | | | 0.000 BDL | µmol/L |
| | Orn | | | 0.000 BDL | µmol/L |
| 25 | Lys | 106.467 | 5975717 | 4.409 | µmol/L |
| | 1-Mhis | | | 0.000 BDL | µmol/L |
| 26 | His | 110.100 | 2100103 | 1.484 | µmol/L |
| | Trp | | | 0.000 BDL | µmol/L |
| | 3-Mhis | | | 0.000 BDL | µmol/L |
| | Ans | | | 0.000 BDL | µmol/L |
| | Car | | | 0.000 BDL | µmol/L |
| 27 | Arg | 123.033 | 4936408 | 3.988 | µmol/L |

| Totals | | | 871037404 | 787.175 | |
|---|---|---|---|---|---|

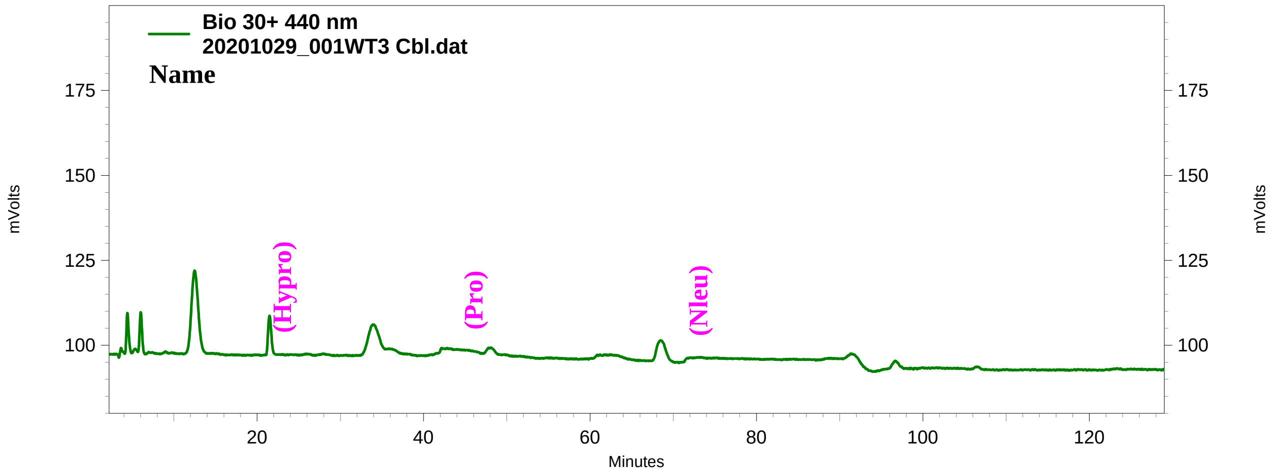


**Bio 30+ 440 nm**
**Results**

| Pk # | Name | Retention Time | Area | ESTD concentration | Units |
|---|---|---|---|---|---|
| | Hypro | | | 0.000 BDL | µmol/L |
| | Pro | | | 0.000 BDL | µmol/L |
| | Nleu | | | 0.000 BDL | µmol/L |

| | | | | | |
|---|---|---|---|---|---|
| Totals | | | | | |
