# Supplementary material for: Pathophysiology of Cerebellar Degeneration in Mitochondrial Disorders: Insights from the Harlequin Mouse
Source: Int J Mol Sci. 2023 Jun 30;24(13):10973. doi: 10.3390/ijms241310973 (PMC10341771; doi:10.3390/ijms241310973)
Supplement: Supplementary file 1 [file ijms-24-10973-s001.zip › Amino acids 6 m brain/20201001_001Hq.4-19_Method Report.pdf]

Biochrom 30+ Final Test

Method: C:\Biochrom\OpenLAB Projects\Default\Method\20180828mod.met
Standard: C:\Biochrom\OpenLAB Projects\Default\Result\20201001_001Hq.4-19.dat
Date : 10/7/2020 10:06:47 AM (GMT +02:00)

Instrument Serial No : 133260
Column No : H-0795
Resin No : 132-56

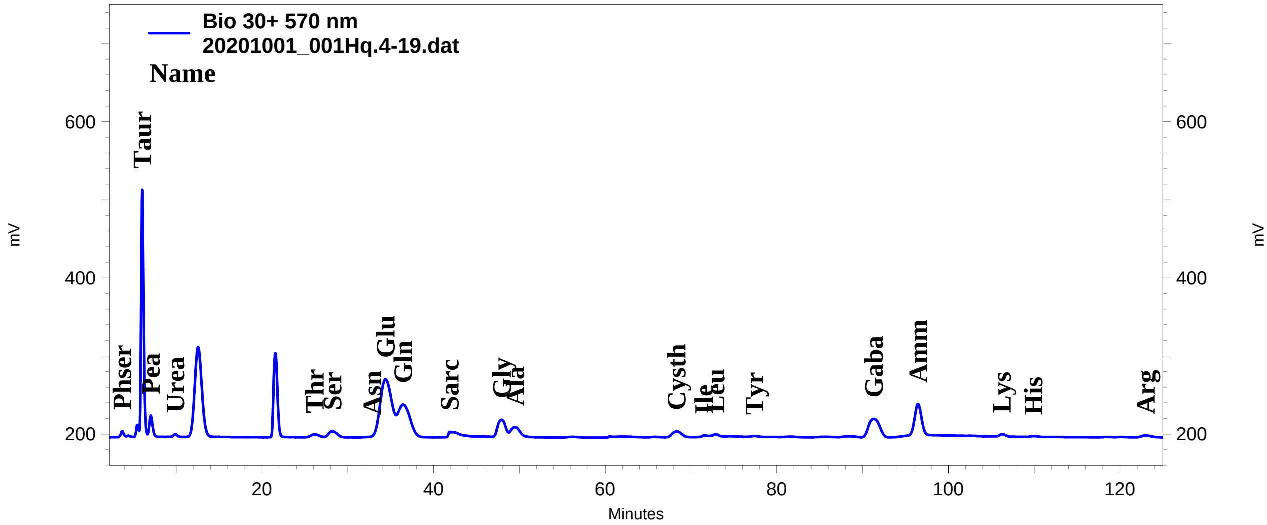

Bio 30+ 570 nm
Results

Pk #	Name	Retention Time	Area	ESTD concentration	Units
1	Phser	3.700	18357407	12.772	µmol/L
4	Taur	6.033	652554311	576.662	µmol/L
5	Pea	7.033	74645958	90.303	µmol/L
6	Urea	9.900	9962347	261.496	µmol/L
	Asp			0.000 BDL	µmol/L
9	Thr	26.133	22734874	17.711	µmol/L
10	Ser	28.200	52689090	40.556	µmol/L
11	Asn	32.833	4996688	6.397	µmol/L
12	Glu	34.400	709082375	561.113	µmol/L
13	Gln	36.433	410605521	324.264	µmol/L
14	Sarc	41.867	12892049	80.451	µmol/L
	AAAA			0.000 BDL	µmol/L
16	Gly	47.933	138033360	100.274	µmol/L
17	Ala	49.500	95007738	75.118	µmol/L
	Citr			0.000 BDL	µmol/L
	Aaba			0.000 BDL	µmol/L
	Val			0.000 BDL	µmol/L
	Cys			0.000 BDL	µmol/L
	Met			0.000 BDL	µmol/L
19	Cysth	68.300	54715346	39.611	µmol/L
20	Ile	71.500	6946835	5.501	µmol/L
21	Leu	72.833	15983193	11.969	µmol/L
	Nleu			0.000 BDL	µmol/L
22	Tyr	77.367	5203521	4.156	µmol/L
	B-ala			0.000 BDL	µmol/L
	Phe			0.000 BDL	µmol/L
	Baiba			0.000 BDL	µmol/L
	Homocys			0.000 BDL	µmol/L
23	Gaba	91.300	209506740	210.024	µmol/L
	Ethan			0.000 BDL	µmol/L
24	Amm	96.467	234854783	173.929	µmol/L
	Hylys			0.000 BDL	µmol/L
	Orn			0.000 BDL	µmol/L
25	Lys	106.200	12171157	8.979	µmol/L
	1-Mhis			0.000 BDL	µmol/L
26	His	109.900	5179386	3.661	µmol/L
	Trp			0.000 BDL	µmol/L
	3-Mhis			0.000 BDL	µmol/L
	Ans			0.000 BDL	µmol/L
	Car			0.000 BDL	µmol/L
27	Arg	123.000	15725296	12.706	µmol/L

Totals			2761847975	2617.656	

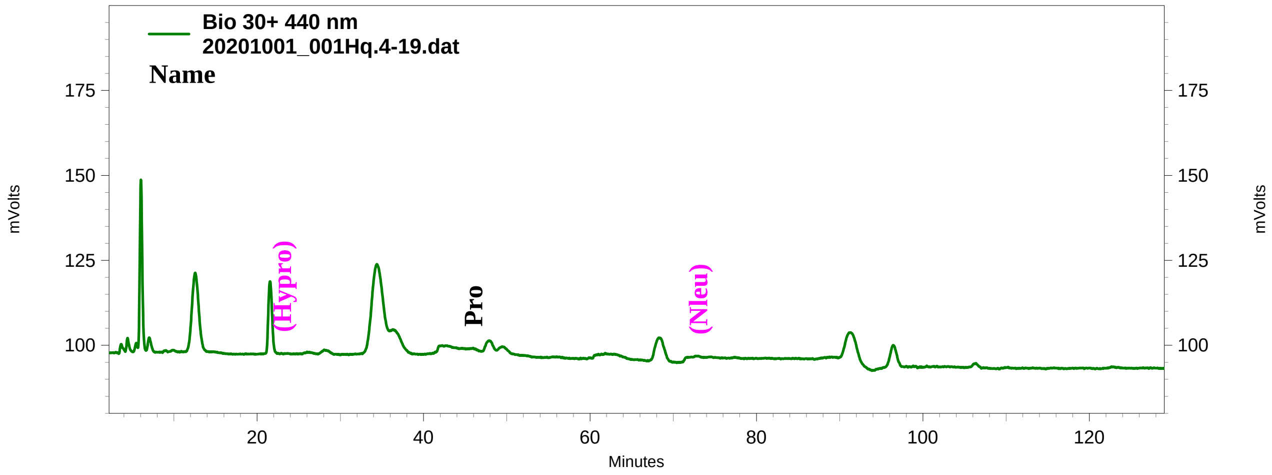

Bio 30+ 440 nm
Results

Pk #	Name	Retention Time	Area	ESTD concentration	Units
	Hypro			0.000 BDL	µmol/L
13	Pro	45.967	2647539	5.743	µmol/L
	Nleu			0.000 BDL	µmol/L

Totals			2647539	5.743	
