# Supplementary material for: Pathophysiology of Cerebellar Degeneration in Mitochondrial Disorders: Insights from the Harlequin Mouse
Source: Int J Mol Sci. 2023 Jun 30;24(13):10973. doi: 10.3390/ijms241310973 (PMC10341771; doi:10.3390/ijms241310973)
Supplement: Supplementary file 1 [file ijms-24-10973-s001.zip › Amino acids 6 m brain/20201001_001Hq.4-61_Method Report.pdf]

Biochrom 30+ Final Test

Method: C:\Biochrom\OpenLAB Projects\Default\Method\20180828mod.met
Standard: C:\Biochrom\OpenLAB Projects\Default\Result\20201001_001Hq.4-61.dat
Date : 10/7/2020 10:07:06 AM (GMT +02:00)

Instrument Serial No : 133260
Column No : H-0795
Resin No : 132-56

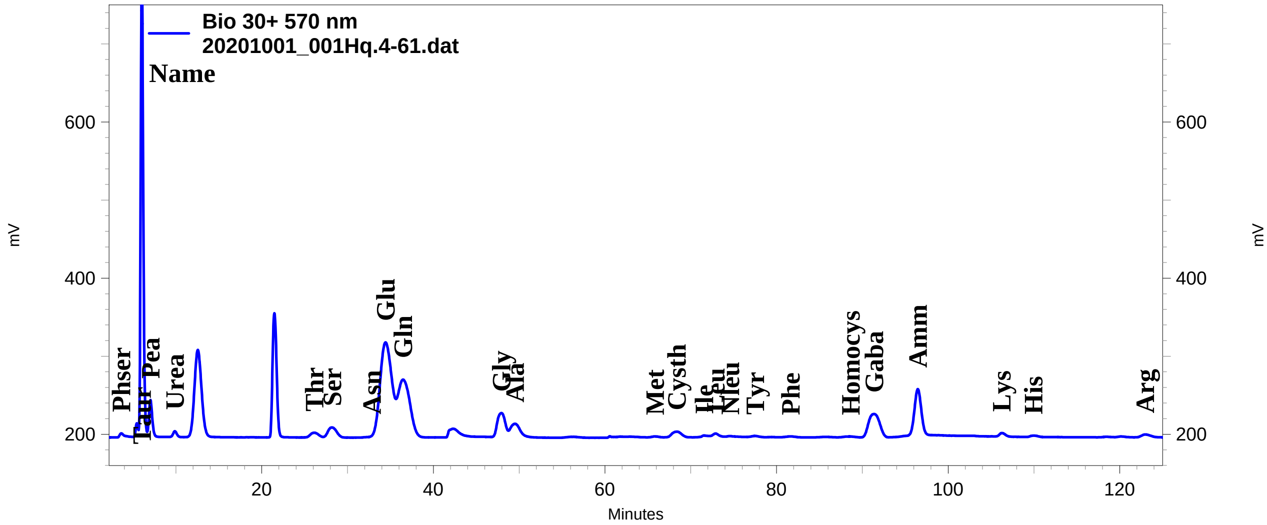

Bio 30+ 570 nm
Results

Pk #	Name	Retention Time	Area	ESTD concentration	Units
1	Phser	3.633	21009552	14.617	µmol/L
3	Taur	6.033	1258898876	1112.489	µmol/L
4	Pea	7.033	130048853	157.327	µmol/L
5	Urea	9.867	21469812	563.549	µmol/L
	Asp			0.000 BDL	µmol/L
8	Thr	26.133	39256543	30.583	µmol/L
9	Ser	28.200	92058341	70.859	µmol/L
10	Asn	32.833	4226086	5.411	µmol/L
11	Glu	34.433	1163242048	920.500	µmol/L
12	Gln	36.467	730648874	577.010	µmol/L
	Sarc			0.000 BDL	µmol/L
	AAAA			0.000 BDL	µmol/L
14	Gly	47.933	198079142	143.895	µmol/L
15	Ala	49.500	134251566	106.146	µmol/L
	Citr			0.000 BDL	µmol/L
	Aaba			0.000 BDL	µmol/L
	Val			0.000 BDL	µmol/L
	Cys			0.000 BDL	µmol/L
17	Met	65.833	6045321	4.688	µmol/L
18	Cysth	68.367	56784896	41.110	µmol/L
19	Ile	71.567	8219432	6.509	µmol/L
20	Leu	72.900	20767810	15.553	µmol/L
21	Nleu	74.600	2072308	0.000	µmol/L
22	Tyr	77.500	6054662	4.836	µmol/L
	B-ala			0.000 BDL	µmol/L
23	Phe	81.633	6251392	4.901	µmol/L
	Baiba			0.000 BDL	µmol/L
24	Homocys	88.633	8624456	3.449	µmol/L
25	Gaba	91.367	269536294	270.202	µmol/L
	Ethan			0.000 BDL	µmol/L
26	Amm	96.467	340121990	251.888	µmol/L
	Hylys			0.000 BDL	µmol/L
	Orn			0.000 BDL	µmol/L
27	Lys	106.233	20844188	15.378	µmol/L
	1-Mhis			0.000 BDL	µmol/L
28	His	109.933	9355846	6.613	µmol/L
	Trp			0.000 BDL	µmol/L
	3-Mhis			0.000 BDL	µmol/L
	Ans			0.000 BDL	µmol/L
	Car			0.000 BDL	µmol/L
30	Arg	122.967	25563578	20.655	µmol/L
Totals			4573431866	4348.165	

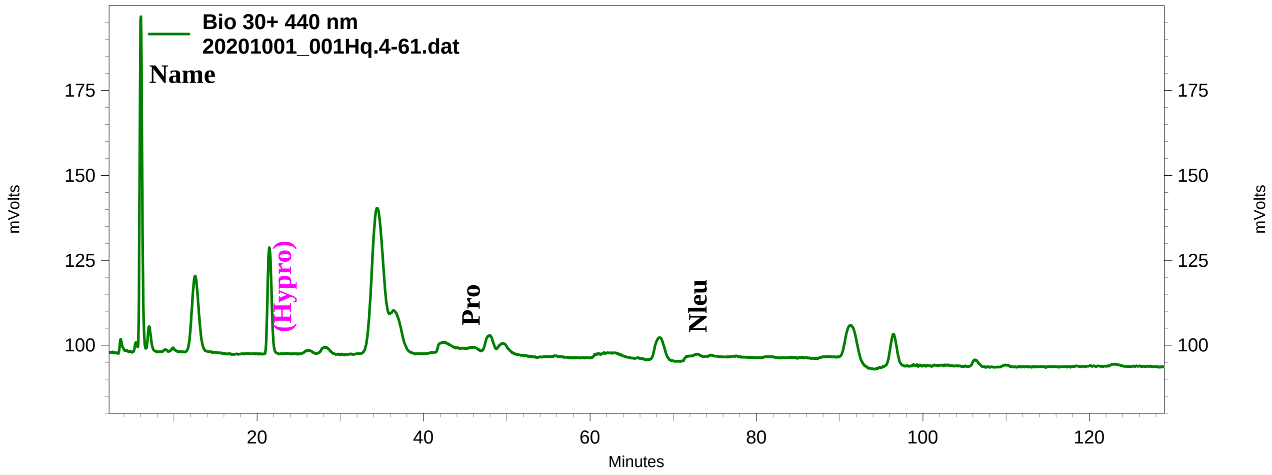

Bio 30+ 440 nm
Results

Pk #	Name	Retention Time	Area	ESTD concentration	Units
	Hypro			0.000 BDL	µmol/L
16	Pro	45.667	4469286	9.694	µmol/L
20	Nleu	72.933	15274927	53.408	µmol/L
Totals			19744213	63.103	
