# Supplementary material for: Pathophysiology of Cerebellar Degeneration in Mitochondrial Disorders: Insights from the Harlequin Mouse
Source: Int J Mol Sci. 2023 Jun 30;24(13):10973. doi: 10.3390/ijms241310973 (PMC10341771; doi:10.3390/ijms241310973)
Supplement: Supplementary file 1 [file ijms-24-10973-s001.zip › Amino acids 6 m brain/20201001_001Hq.55_Method Report.pdf]

Biochrom 30+ Final Test

Method: C:\Biochrom\OpenLAB Projects\Default\Method\20180828mod.met
Standard: C:\Biochrom\OpenLAB Projects\Default\Result\20201001_001Hq.55.dat
Date : 10/7/2020 10:06:56 AM (GMT +02:00)

Instrument Serial No : 133260
Column No : H-0795
Resin No : 132-56

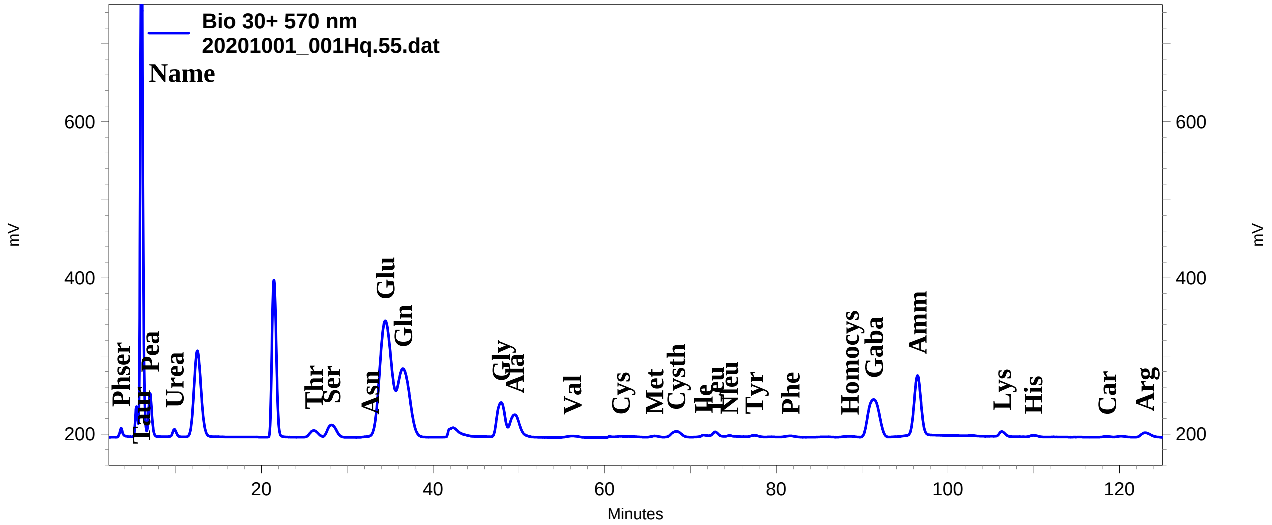

Bio 30+ 570 nm
Results

Pk #	Name	Retention Time	Area	ESTD concentration	Units
1	Phser	3.633	28170058	19.599	µmol/L
3	Taur	6.000	1401290143	1238.320	µmol/L
4	Pea	7.000	151143192	182.846	µmol/L
5	Urea	9.867	27946625	733.555	µmol/L
	Asp			0.000 BDL	µmol/L
8	Thr	26.100	56559931	44.063	µmol/L
9	Ser	28.133	112618625	86.685	µmol/L
10	Asn	32.667	5281598	6.762	µmol/L
11	Glu	34.433	1440321376	1139.759	µmol/L
12	Gln	36.500	864260842	682.526	µmol/L
	Sarc			0.000 BDL	µmol/L
	AAAA			0.000 BDL	µmol/L
14	Gly	47.933	279728029	203.208	µmol/L
15	Ala	49.533	220040747	173.975	µmol/L
	Citr			0.000 BDL	µmol/L
	Aaba			0.000 BDL	µmol/L
16	Val	56.233	15222113	12.578	µmol/L
18	Cys	61.867	4550680	3.093	µmol/L
19	Met	65.800	8598932	6.668	µmol/L
20	Cysth	68.333	58635663	42.449	µmol/L
21	Ile	71.533	13206038	10.458	µmol/L
22	Leu	72.867	31307230	23.445	µmol/L
23	Nleu	74.500	4443674	0.000	µmol/L
24	Tyr	77.400	9915483	7.920	µmol/L
	B-ala			0.000 BDL	µmol/L
25	Phe	81.667	10242441	8.030	µmol/L
	Baiba			0.000 BDL	µmol/L
26	Homocys	88.567	6606705	2.642	µmol/L
27	Gaba	91.367	436845128	437.925	µmol/L
	Ethan			0.000 BDL	µmol/L
28	Amm	96.467	439160546	325.235	µmol/L
	Hylys			0.000 BDL	µmol/L
	Orn			0.000 BDL	µmol/L
29	Lys	106.300	28077942	20.714	µmol/L
	1-Mhis			0.000 BDL	µmol/L
30	His	109.967	10490568	7.415	µmol/L
	Trp			0.000 BDL	µmol/L
	3-Mhis			0.000 BDL	µmol/L
	Ans			0.000 BDL	µmol/L
31	Car	118.567	4729602	8.279	µmol/L
33	Arg	122.967	43727934	35.331	µmol/L

Totals			5713121845	5463.480	

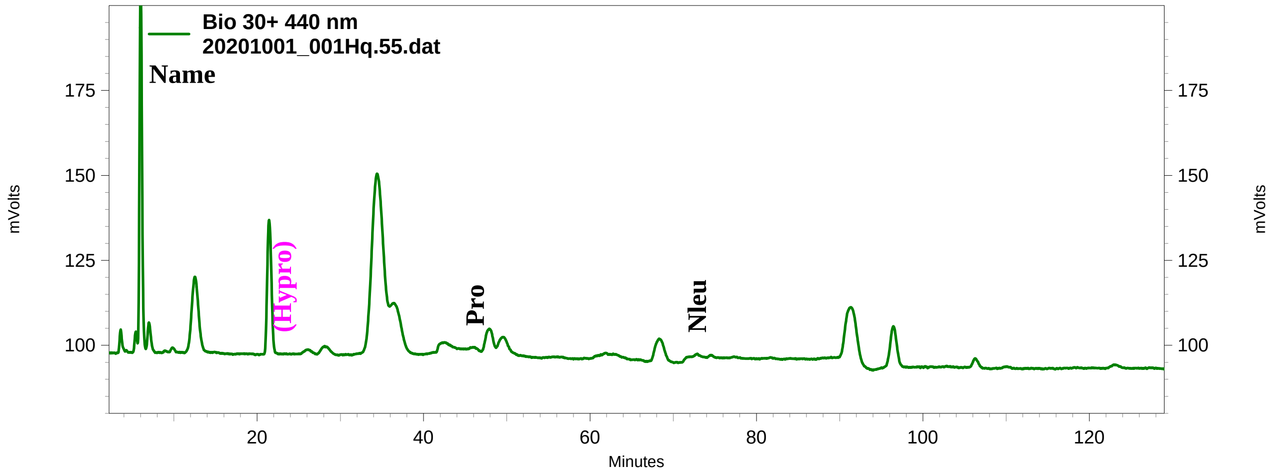

Bio 30+ 440 nm
Results

Pk #	Name	Retention Time	Area	ESTD concentration	Units
	Hypro			0.000 BDL	µmol/L
15	Pro	46.167	3709238	8.046	µmol/L
20	Nleu	72.867	13212808	46.198	µmol/L

Totals			16922046	54.244	
