# Supplementary material for: Pathophysiology of Cerebellar Degeneration in Mitochondrial Disorders: Insights from the Harlequin Mouse
Source: Int J Mol Sci. 2023 Jun 30;24(13):10973. doi: 10.3390/ijms241310973 (PMC10341771; doi:10.3390/ijms241310973)
Supplement: Supplementary file 1 [file ijms-24-10973-s001.zip › Amino acids 6 m brain/20201001_001Hq.76_Method Report.pdf]

Biochrom 30+ Final Test

Method: C:\Biochrom\OpenLAB Projects\Default\Method\20180828mod.met
Standard: C:\Biochrom\OpenLAB Projects\Default\Result\20201001_001Hq.76.dat
Date : 10/7/2020 10:06:19 AM (GMT +02:00)

Instrument Serial No : 133260
Column No : H-0795
Resin No : 132-56

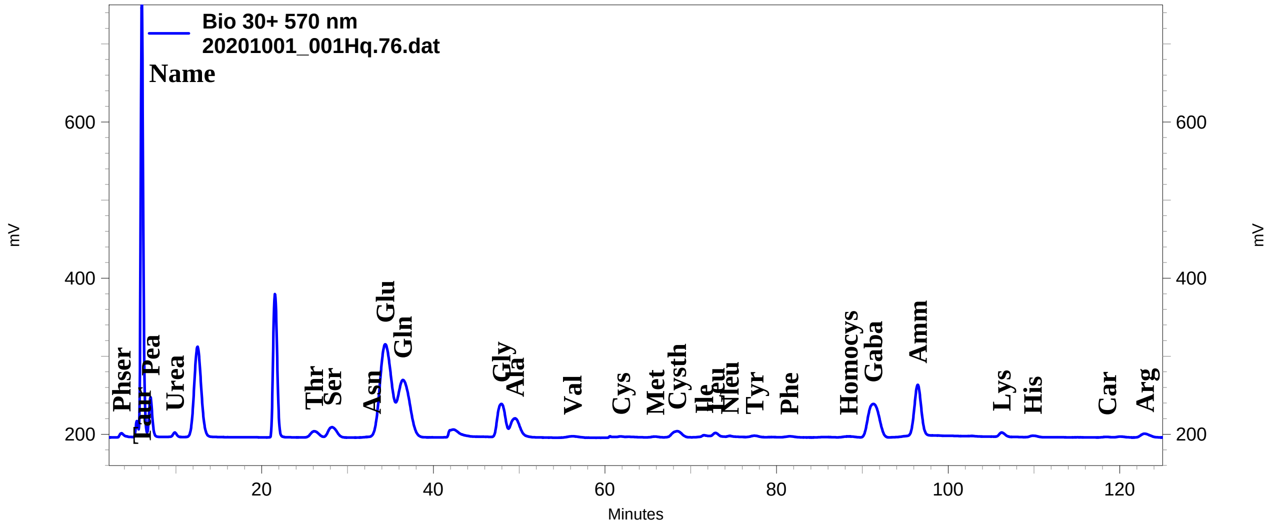

Bio 30+ 570 nm
Results

Pk #	Name	Retention Time	Area	ESTD concentration	Units
1	Phser	3.667	20056167	13.954	µmol/L
3	Taur	6.033	1146241197	1012.933	µmol/L
4	Pea	7.033	137596760	166.458	µmol/L
5	Urea	9.867	16838834	441.993	µmol/L
	Asp			0.000 BDL	µmol/L
8	Thr	26.100	51331914	39.990	µmol/L
9	Ser	28.200	92051054	70.853	µmol/L
10	Asn	32.833	3253679	4.166	µmol/L
11	Glu	34.400	1122899279	888.576	µmol/L
12	Gln	36.433	705923412	557.484	µmol/L
	Sarc			0.000 BDL	µmol/L
	AAAA			0.000 BDL	µmol/L
14	Gly	47.933	268728255	195.218	µmol/L
15	Ala	49.500	182111018	143.986	µmol/L
	Citr			0.000 BDL	µmol/L
	Aaba			0.000 BDL	µmol/L
16	Val	56.233	13845312	11.440	µmol/L
18	Cys	61.867	4431801	3.012	µmol/L
19	Met	65.867	5358146	4.155	µmol/L
20	Cysth	68.400	62598306	45.318	µmol/L
21	Ile	71.567	12768026	10.111	µmol/L
22	Leu	72.900	27621713	20.685	µmol/L
23	Nleu	74.533	4483121	0.000	µmol/L
24	Tyr	77.433	9237861	7.379	µmol/L
	B-ala			0.000 BDL	µmol/L
25	Phe	81.467	8019260	6.287	µmol/L
	Baiba			0.000 BDL	µmol/L
26	Homocys	88.400	9257344	3.702	µmol/L
27	Gaba	91.267	383696604	384.645	µmol/L
	Ethan			0.000 BDL	µmol/L
28	Amm	96.467	370435102	274.338	µmol/L
	Hylys			0.000 BDL	µmol/L
	Orn			0.000 BDL	µmol/L
29	Lys	106.233	24888714	18.361	µmol/L
	1-Mhis			0.000 BDL	µmol/L
30	His	109.867	9824806	6.945	µmol/L
	Trp			0.000 BDL	µmol/L
	3-Mhis			0.000 BDL	µmol/L
	Ans			0.000 BDL	µmol/L
31	Car	118.533	2757960	4.828	µmol/L
33	Arg	122.933	36661926	29.622	µmol/L

Totals			4732917571	4366.438	

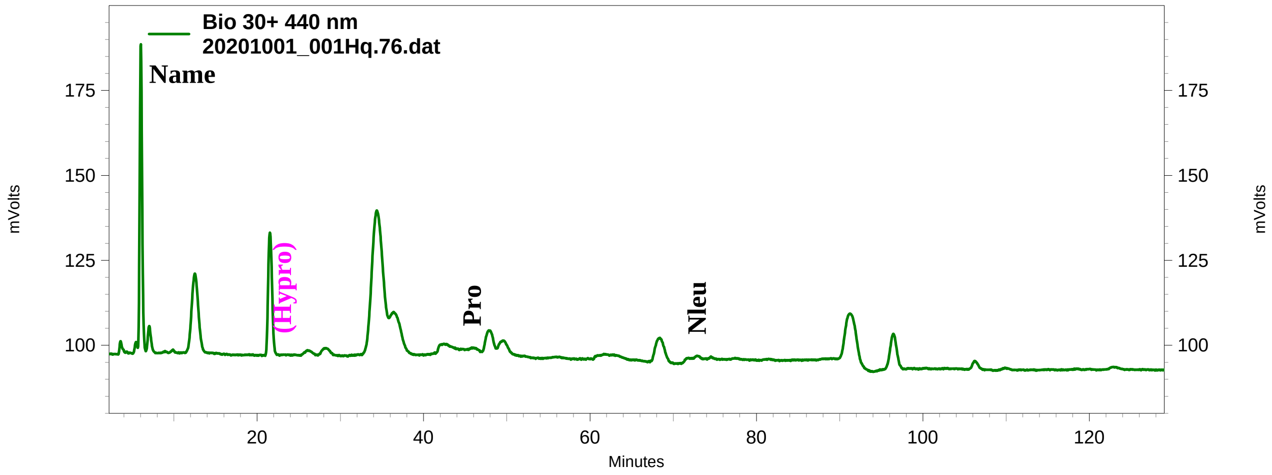

Bio 30+ 440 nm
Results

Pk #	Name	Retention Time	Area	ESTD concentration	Units
	Hypro			0.000 BDL	µmol/L
16	Pro	45.800	3342512	7.250	µmol/L
21	Nleu	72.833	15545949	54.356	µmol/L

Totals			18888461	61.606	
