# Supplementary material for: Pathophysiology of Cerebellar Degeneration in Mitochondrial Disorders: Insights from the Harlequin Mouse
Source: Int J Mol Sci. 2023 Jun 30;24(13):10973. doi: 10.3390/ijms241310973 (PMC10341771; doi:10.3390/ijms241310973)
Supplement: Supplementary file 1 [file ijms-24-10973-s001.zip › Amino acids 6 m brain/20201001_001Hq.8-93_Method Report.pdf]

Biochrom 30+ Final Test

Method: C:\Biochrom\OpenLAB Projects\Default\Method\20180828mod.met
Standard: C:\Biochrom\OpenLAB Projects\Default\Result\20201001_001Hq.8-93.dat
Date : 10/7/2020 10:06:32 AM (GMT +02:00)

Instrument Serial No : 133260
Column No : H-0795
Resin No : 132-56

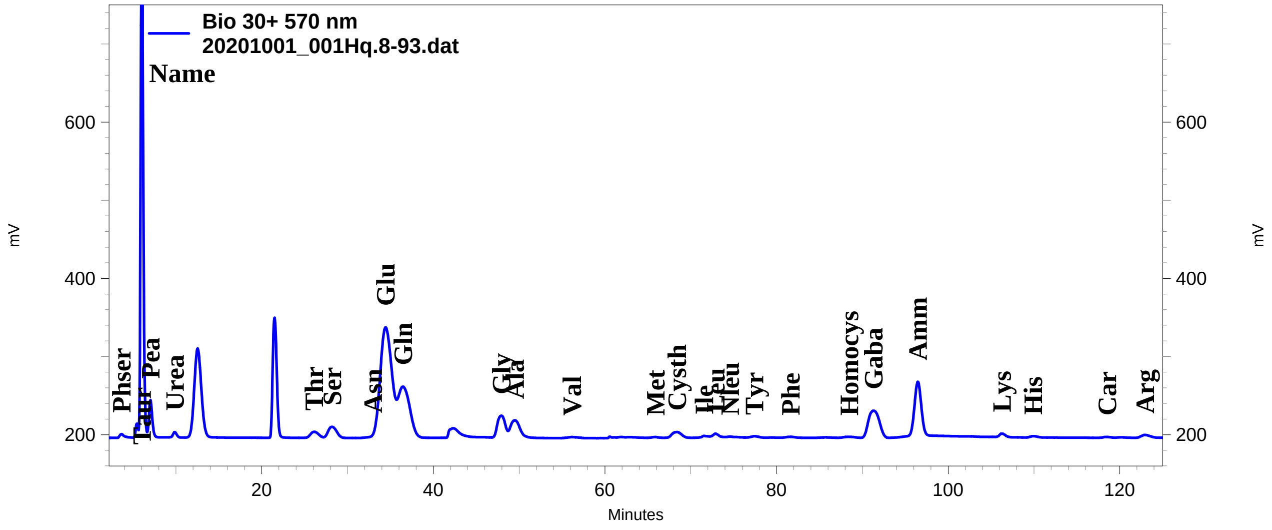

Bio 30+ 570 nm
Results

Pk #	Name	Retention Time	Area	ESTD concentration	Units
1	Phser	3.667	15344150	10.676	µmol/L
3	Taur	6.033	1386732836	1225.455	µmol/L
4	Pea	7.033	129321282	156.447	µmol/L
5	Urea	9.867	19763342	518.757	µmol/L
	Asp			0.000 BDL	µmol/L
8	Thr	26.100	50076142	39.012	µmol/L
9	Ser	28.200	98415600	75.752	µmol/L
10	Asn	32.967	8503110	10.887	µmol/L
11	Glu	34.433	1348031655	1066.728	µmol/L
12	Gln	36.467	617900829	487.970	µmol/L
	Sarc			0.000 BDL	µmol/L
	AAAA			0.000 BDL	µmol/L
14	Gly	47.933	175659153	127.608	µmol/L
15	Ala	49.500	172744830	136.580	µmol/L
	Citr			0.000 BDL	µmol/L
	Aaba			0.000 BDL	µmol/L
16	Val	56.167	9677145	7.996	µmol/L
	Cys			0.000 BDL	µmol/L
18	Met	65.900	5337632	4.139	µmol/L
19	Cysth	68.400	58358760	42.249	µmol/L
20	Ile	71.567	8207895	6.500	µmol/L
21	Leu	72.900	22203822	16.628	µmol/L
22	Nleu	74.567	2478943	0.000	µmol/L
23	Tyr	77.400	8322926	6.648	µmol/L
	B-ala			0.000 BDL	µmol/L
24	Phe	81.633	7374540	5.782	µmol/L
	Baiba			0.000 BDL	µmol/L
25	Homocys	88.467	9699422	3.879	µmol/L
26	Gaba	91.300	309031804	309.796	µmol/L
	Ethan			0.000 BDL	µmol/L
27	Amm	96.467	393563598	291.466	µmol/L
	Hylys			0.000 BDL	µmol/L
	Orn			0.000 BDL	µmol/L
28	Lys	106.267	20100582	14.829	µmol/L
	1-Mhis			0.000 BDL	µmol/L
29	His	109.900	8538376	6.035	µmol/L
	Trp			0.000 BDL	µmol/L
	3-Mhis			0.000 BDL	µmol/L
	Ans			0.000 BDL	µmol/L
30	Car	118.533	4559822	7.982	µmol/L
31	Arg	122.967	25019360	20.215	µmol/L
Totals			4914967556	4600.015	

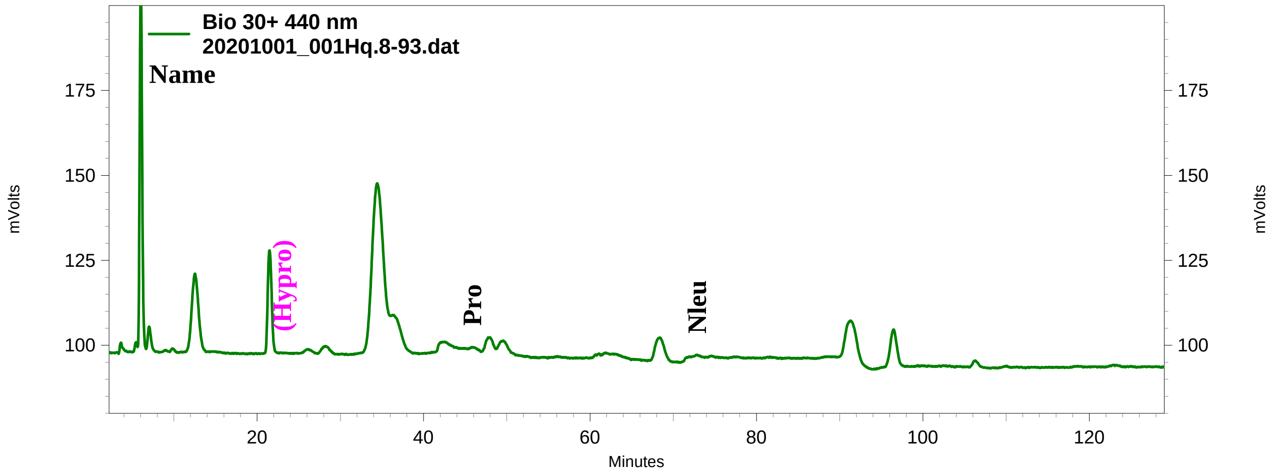

Bio 30+ 440 nm
Results

Pk #	Name	Retention Time	Area	ESTD concentration	Units
	Hypro			0.000 BDL	µmol/L
14	Pro	45.833	2996248	6.499	µmol/L
19	Nleu	72.867	10907346	38.137	µmol/L

Totals			13903594	44.636	
