# Supplementary material for: Pathophysiology of Cerebellar Degeneration in Mitochondrial Disorders: Insights from the Harlequin Mouse
Source: Int J Mol Sci. 2023 Jun 30;24(13):10973. doi: 10.3390/ijms241310973 (PMC10341771; doi:10.3390/ijms241310973)
Supplement: Supplementary file 1 [file ijms-24-10973-s001.zip › Amino acids 6 m brain/20201001_001WT3-23_Method Report.pdf]

Biochrom 30+ Final Test

Method: C:\Biochrom\OpenLAB Projects\Default\Method\20180828mod.met
Standard: C:\Biochrom\OpenLAB Projects\Default\Result\20201001_001WT3-23.dat
Date : 10/7/2020 10:07:54 AM (GMT +02:00)

Instrument Serial No : 133260
Column No : H-0795
Resin No : 132-56

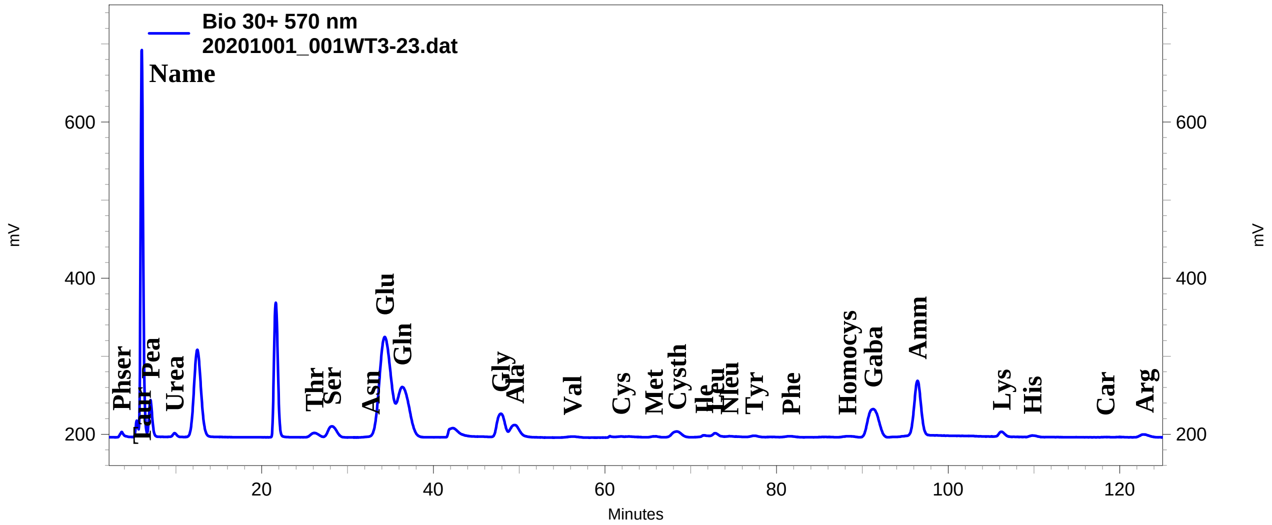

Bio 30+ 570 nm
Results

Pk #	Name	Retention Time	Area	ESTD concentration	Units
1	Phser	3.667	19917553	13.858	µmol/L
3	Taur	6.033	1017882411	899.502	µmol/L
4	Pea	7.033	126909988	153.530	µmol/L
5	Urea	9.833	14030728	368.284	µmol/L
	Asp			0.000 BDL	µmol/L
8	Thr	26.133	35692152	27.806	µmol/L
9	Ser	28.167	98049191	75.470	µmol/L
10	Asn	32.700	4161505	5.328	µmol/L
11	Glu	34.333	1208128108	956.019	µmol/L
12	Gln	36.367	616781064	487.086	µmol/L
	Sarc			0.000 BDL	µmol/L
	AAAA			0.000 BDL	µmol/L
14	Gly	47.867	185675054	134.884	µmol/L
15	Ala	49.500	118389886	93.605	µmol/L
	Citr			0.000 BDL	µmol/L
	Aaba			0.000 BDL	µmol/L
16	Val	56.200	9748879	8.056	µmol/L
18	Cys	61.867	2766826	1.880	µmol/L
19	Met	65.700	6024776	4.672	µmol/L
20	Cysth	68.400	55254212	40.001	µmol/L
21	Ile	71.567	7941298	6.289	µmol/L
22	Leu	72.833	21540525	16.131	µmol/L
23	Nleu	74.500	1929860	0.000	µmol/L
24	Tyr	77.367	7015495	5.603	µmol/L
	B-ala			0.000 BDL	µmol/L
25	Phe	81.700	7107407	5.572	µmol/L
	Baiba			0.000 BDL	µmol/L
26	Homocys	88.233	10439986	4.175	µmol/L
27	Gaba	91.267	316724053	317.507	µmol/L
	Ethan			0.000 BDL	µmol/L
28	Amm	96.433	395129830	292.626	µmol/L
	Hylys			0.000 BDL	µmol/L
	Orn			0.000 BDL	µmol/L
29	Lys	106.233	29350476	21.653	µmol/L
	1-Mhis			0.000 BDL	µmol/L
30	His	109.833	10609735	7.500	µmol/L
	Trp			0.000 BDL	µmol/L
	3-Mhis			0.000 BDL	µmol/L
	Ans			0.000 BDL	µmol/L
31	Car	118.333	1441898	2.524	µmol/L
32	Arg	122.900	26331336	21.275	µmol/L

Totals					
			4354974232	3970.836	

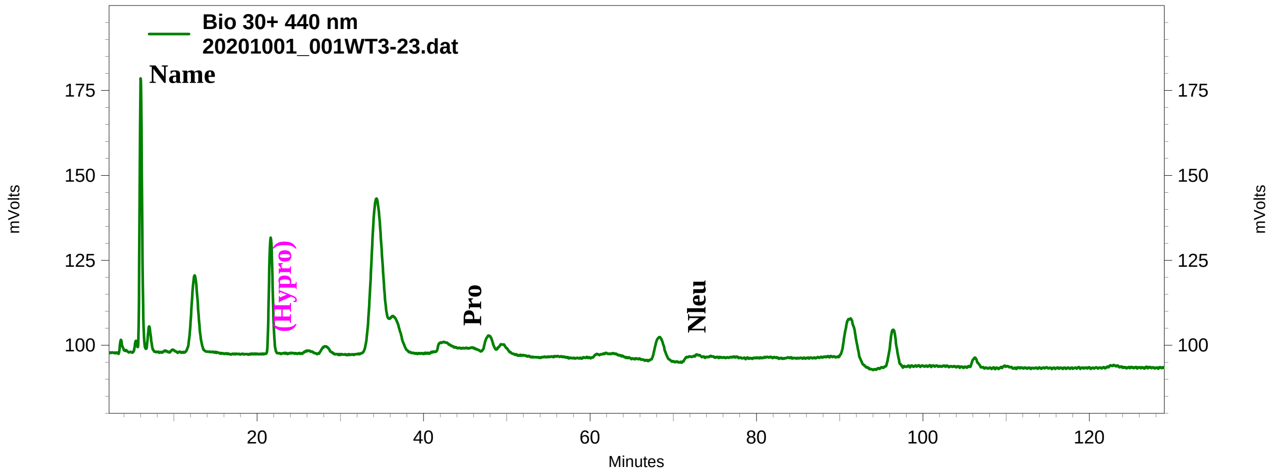

Bio 30+ 440 nm
Results

Pk #	Name	Retention Time	Area	ESTD concentration	Units
	Hypro			0.000 BDL	µmol/L
14	Pro	45.833	4685571	10.164	µmol/L
20	Nleu	72.800	15017122	52.507	µmol/L

Totals			19702693	62.670	
