# Supplementary material for: Pathophysiology of Cerebellar Degeneration in Mitochondrial Disorders: Insights from the Harlequin Mouse
Source: Int J Mol Sci. 2023 Jun 30;24(13):10973. doi: 10.3390/ijms241310973 (PMC10341771; doi:10.3390/ijms241310973)
Supplement: Supplementary file 1 [file ijms-24-10973-s001.zip › Amino acids 6 m brain/20201001_001WT3-25_Method Report.pdf]

Biochrom 30+ Final Test

Method: C:\Biochrom\OpenLAB Projects\Default\Method\20180828mod.met
Standard: C:\Biochrom\OpenLAB Projects\Default\Result\20201001_001WT3-25.dat
Date : 10/7/2020 10:05:51 AM (GMT +02:00)

Instrument Serial No : 133260
Column No : H-0795
Resin No : 132-56

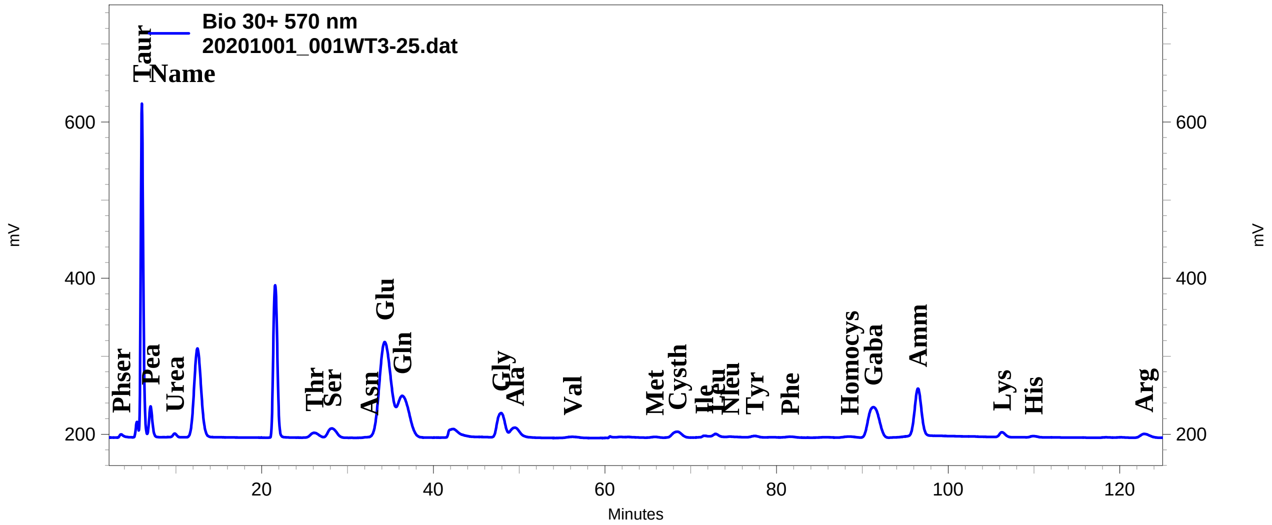

Bio 30+ 570 nm
Results

Pk #	Name	Retention Time	Area	ESTD concentration	Units
1	Phser	3.633	14218425	9.892	µmol/L
3	Taur	6.033	870376812	769.152	µmol/L
4	Pea	7.033	106287981	128.582	µmol/L
5	Urea	9.867	13270234	348.323	µmol/L
	Asp			0.000 BDL	µmol/L
8	Thr	26.133	39619489	30.865	µmol/L
9	Ser	28.200	82292501	63.342	µmol/L
10	Asn	32.533	2805654	3.592	µmol/L
11	Glu	34.333	1161597721	919.199	µmol/L
12	Gln	36.367	508188075	401.328	µmol/L
	Sarc			0.000 BDL	µmol/L
	AAAA			0.000 BDL	µmol/L
14	Gly	47.900	193733458	140.738	µmol/L
15	Ala	49.500	95741096	75.697	µmol/L
	Citr			0.000 BDL	µmol/L
	Aaba			0.000 BDL	µmol/L
16	Val	56.233	9807556	8.104	µmol/L
	Cys			0.000 BDL	µmol/L
18	Met	65.800	4898230	3.798	µmol/L
19	Cysth	68.433	58559597	42.394	µmol/L
20	Ile	71.567	8699176	6.889	µmol/L
21	Leu	72.933	20884461	15.640	µmol/L
22	Nleu	74.600	2529320	0.000	µmol/L
23	Tyr	77.433	9156234	7.313	µmol/L
	B-ala			0.000 BDL	µmol/L
24	Phe	81.567	5901020	4.626	µmol/L
	Baiba			0.000 BDL	µmol/L
25	Homocys	88.500	10103290	4.040	µmol/L
26	Gaba	91.267	343052560	343.900	µmol/L
	Ethan			0.000 BDL	µmol/L
27	Amm	96.467	343744648	254.571	µmol/L
	Hylys			0.000 BDL	µmol/L
	Orn			0.000 BDL	µmol/L
28	Lys	106.267	27680490	20.421	µmol/L
	1-Mhis			0.000 BDL	µmol/L
29	His	109.967	8908332	6.297	µmol/L
	Trp			0.000 BDL	µmol/L
	3-Mhis			0.000 BDL	µmol/L
	Ans			0.000 BDL	µmol/L
	Car			0.000 BDL	µmol/L
30	Arg	122.833	36333347	29.356	µmol/L

Totals			3978389707	3638.062	

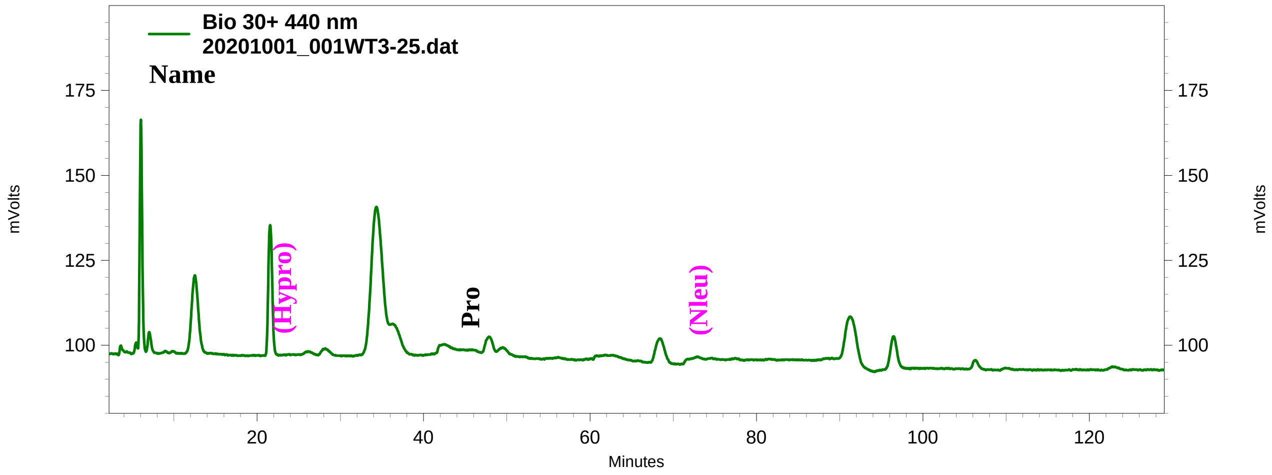

Bio 30+ 440 nm
Results

Pk #	Name	Retention Time	Area	ESTD concentration	Units
	Hypro			0.000 BDL	µmol/L
15	Pro	45.600	3168161	6.872	µmol/L
	Nleu			0.000 BDL	µmol/L

Totals					
			3168161	6.872	
