# Supplementary material for: Pathophysiology of Cerebellar Degeneration in Mitochondrial Disorders: Insights from the Harlequin Mouse
Source: Int J Mol Sci. 2023 Jun 30;24(13):10973. doi: 10.3390/ijms241310973 (PMC10341771; doi:10.3390/ijms241310973)
Supplement: Supplementary file 1 [file ijms-24-10973-s001.zip › Amino acids 6 m brain/20201001_001WT4-19_Method Report.pdf]

Biochrom 30+ Final Test

Method: C:\Biochrom\OpenLAB Projects\Default\Method\20180828mod.met
Standard: C:\Biochrom\OpenLAB Projects\Default\Result\20201001_001WT4-19.dat
Date : 10/7/2020 10:07:46 AM (GMT +02:00)

Instrument Serial No : 133260
Column No : H-0795
Resin No : 132-56

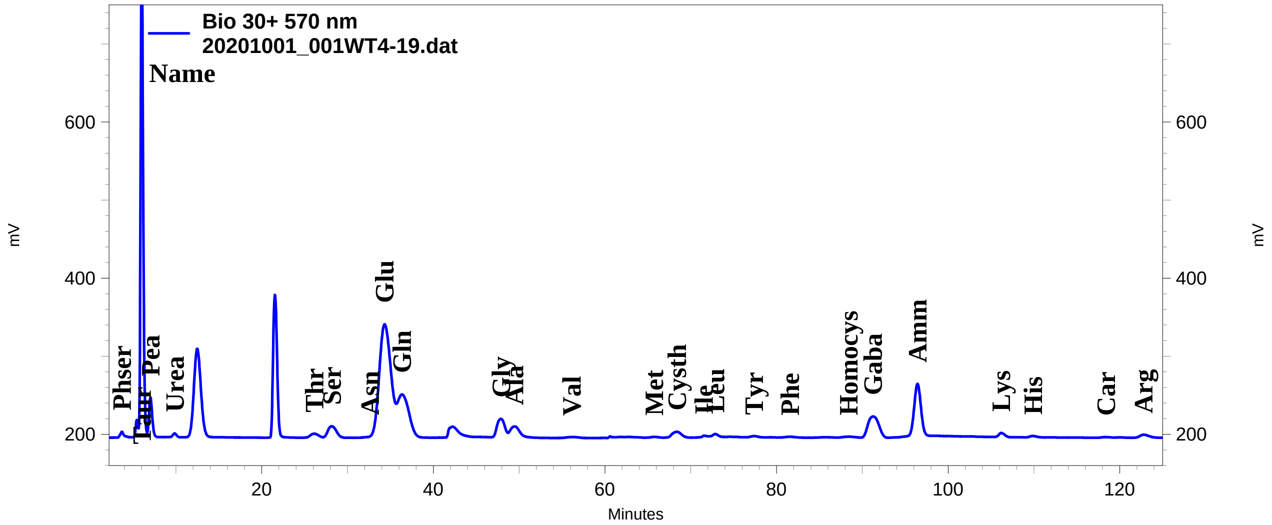

Bio 30+ 570 nm
Results

Pk #	Name	Retention Time	Area	ESTD concentration	Units
1	Phser	3.700	20613217	14.342	µmol/L
3	Taur	6.033	1288400112	1138.559	µmol/L
4	Pea	7.033	136238754	164.816	µmol/L
5	Urea	9.867	13733721	360.489	µmol/L
	Asp			0.000 BDL	µmol/L
8	Thr	26.133	32063663	24.979	µmol/L
9	Ser	28.167	100819634	77.603	µmol/L
10	Asn	32.600	2959822	3.790	µmol/L
11	Glu	34.300	1381362685	1093.104	µmol/L
12	Gln	36.333	512335925	404.603	µmol/L
	Sarc			0.000 BDL	µmol/L
	AAAA			0.000 BDL	µmol/L
14	Gly	47.900	147872141	107.422	µmol/L
15	Ala	49.433	108728965	85.966	µmol/L
	Citr			0.000 BDL	µmol/L
	Aaba			0.000 BDL	µmol/L
16	Val	56.133	8080311	6.677	µmol/L
	Cys			0.000 BDL	µmol/L
18	Met	65.733	4480938	3.475	µmol/L
19	Cysth	68.400	56529440	40.925	µmol/L
20	Ile	71.533	9408901	7.451	µmol/L
21	Leu	72.867	20499426	15.352	µmol/L
	Nleu			0.000 BDL	µmol/L
22	Tyr	77.367	8579254	6.852	µmol/L
	B-ala			0.000 BDL	µmol/L
23	Phe	81.567	5874681	4.606	µmol/L
	Baiba			0.000 BDL	µmol/L
24	Homocys	88.400	9827608	3.930	µmol/L
25	Gaba	91.233	239842942	240.436	µmol/L
	Ethan			0.000 BDL	µmol/L
26	Amm	96.433	374734478	277.522	µmol/L
	Hylys			0.000 BDL	µmol/L
	Orn			0.000 BDL	µmol/L
27	Lys	106.167	23862050	17.604	µmol/L
	1-Mhis			0.000 BDL	µmol/L
28	His	109.900	9143979	6.464	µmol/L
	Trp			0.000 BDL	µmol/L
	3-Mhis			0.000 BDL	µmol/L
	Ans			0.000 BDL	µmol/L
29	Car	118.400	3045373	5.331	µmol/L
30	Arg	122.767	27082280	21.882	µmol/L

Totals			4546120300	4134.175	

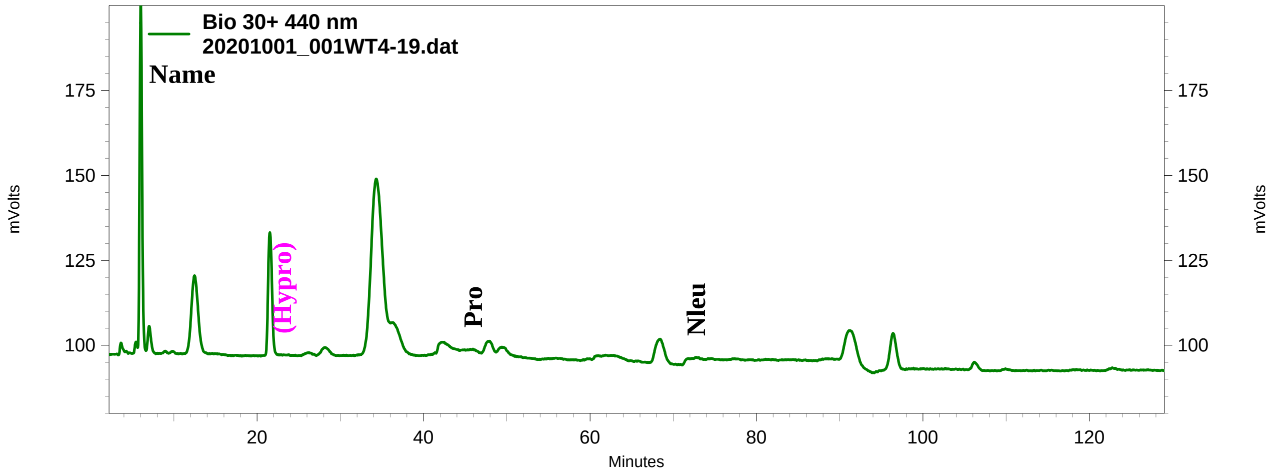

Bio 30+ 440 nm
Results

Pk #	Name	Retention Time	Area	ESTD concentration	Units
	Hypro			0.000 BDL	µmol/L
15	Pro	45.933	3148423	6.829	µmol/L
19	Nleu	72.733	1198676	4.191	µmol/L

Totals			4347099	11.020	
