# Supplementary material for: Pathophysiology of Cerebellar Degeneration in Mitochondrial Disorders: Insights from the Harlequin Mouse
Source: Int J Mol Sci. 2023 Jun 30;24(13):10973. doi: 10.3390/ijms241310973 (PMC10341771; doi:10.3390/ijms241310973)
Supplement: Supplementary file 1 [file ijms-24-10973-s001.zip › Amino acids 6 m brain/20201001_001WT4-60_Method Report.pdf]

Biochrom 30+ Final Test

Method: C:\Biochrom\OpenLAB Projects\Default\Method\20180828mod.met
Standard: C:\Biochrom\OpenLAB Projects\Default\Result\20201001_001WT4-60.dat
Date : 10/7/2020 10:07:30 AM (GMT +02:00)

Instrument Serial No : 133260
Column No : H-0795
Resin No : 132-56

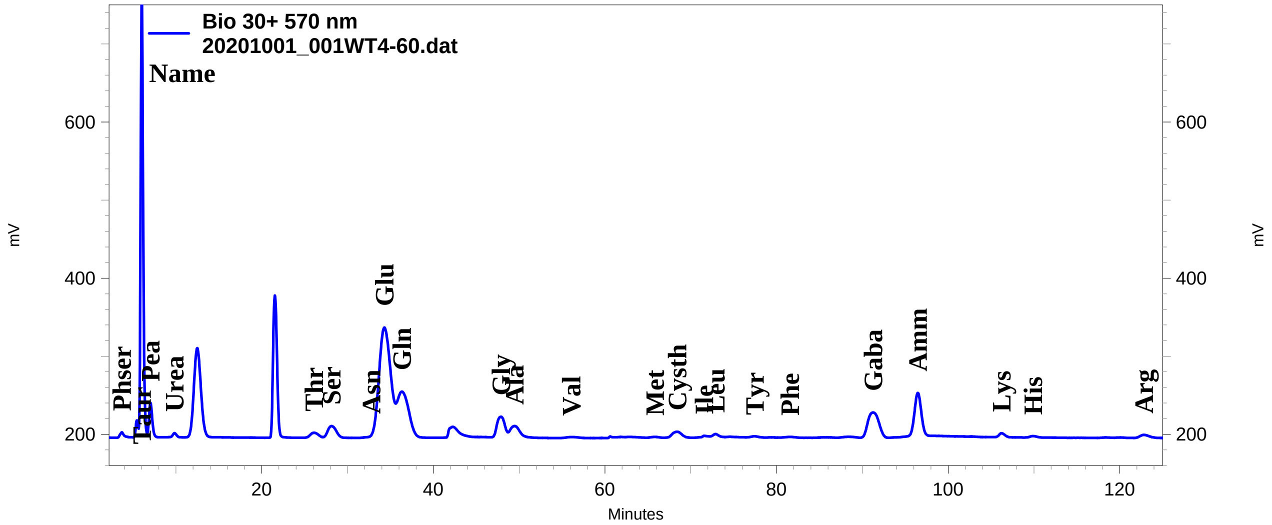

Bio 30+ 570 nm
Results

Pk #	Name	Retention Time	Area	ESTD concentration	Units
1	Phser	3.700	20726447	14.420	µmol/L
3	Taur	6.033	1148595179	1015.013	µmol/L
4	Pea	7.033	118807476	143.728	µmol/L
5	Urea	9.833	15399900	404.223	µmol/L
	Asp			0.000 BDL	µmol/L
8	Thr	26.100	40774860	31.765	µmol/L
9	Ser	28.133	103082038	79.344	µmol/L
10	Asn	32.767	5924217	7.585	µmol/L
11	Glu	34.300	1332784212	1054.662	µmol/L
12	Gln	36.333	557534185	440.297	µmol/L
	Sarc			0.000 BDL	µmol/L
	AAAA			0.000 BDL	µmol/L
14	Gly	47.900	166755282	121.139	µmol/L
15	Ala	49.467	114610901	90.617	µmol/L
	Citr			0.000 BDL	µmol/L
	Aaba			0.000 BDL	µmol/L
16	Val	56.100	8025202	6.631	µmol/L
	Cys			0.000 BDL	µmol/L
18	Met	65.833	5606615	4.348	µmol/L
19	Cysth	68.433	58989758	42.706	µmol/L
20	Ile	71.567	6846665	5.422	µmol/L
21	Leu	72.900	18513998	13.865	µmol/L
	Nleu			0.000 BDL	µmol/L
22	Tyr	77.467	7646198	6.107	µmol/L
	B-ala			0.000 BDL	µmol/L
23	Phe	81.567	5303869	4.158	µmol/L
	Baiba			0.000 BDL	µmol/L
	Homocys			0.000 BDL	µmol/L
24	Gaba	91.267	287032954	287.742	µmol/L
	Ethan			0.000 BDL	µmol/L
25	Amm	96.467	312975195	231.784	µmol/L
	Hylys			0.000 BDL	µmol/L
	Orn			0.000 BDL	µmol/L
26	Lys	106.233	22536174	16.626	µmol/L
	1-Mhis			0.000 BDL	µmol/L
27	His	109.900	9435467	6.670	µmol/L
	Trp			0.000 BDL	µmol/L
	3-Mhis			0.000 BDL	µmol/L
	Ans			0.000 BDL	µmol/L
	Car			0.000 BDL	µmol/L
28	Arg	122.833	28566918	23.081	µmol/L

Totals			4396473710	4051.935	

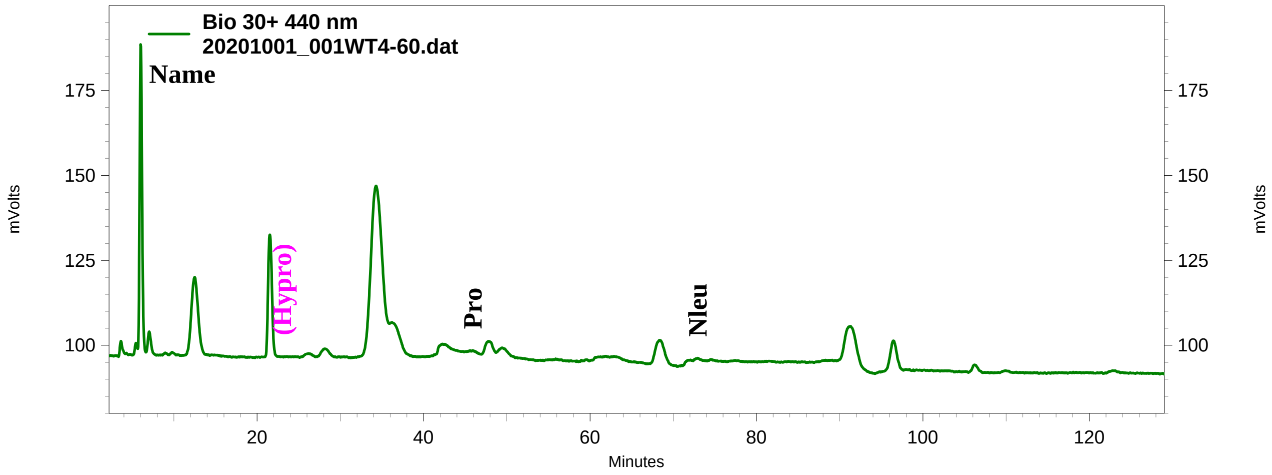

Bio 30+ 440 nm
Results

Pk #	Name	Retention Time	Area	ESTD concentration	Units
	Hypro			0.000 BDL	µmol/L
15	Pro	45.900	4478091	9.713	µmol/L
20	Nleu	72.933	12205231	42.675	µmol/L

Totals			16683322	52.389	
