# Supplementary material for: Pathophysiology of Cerebellar Degeneration in Mitochondrial Disorders: Insights from the Harlequin Mouse
Source: Int J Mol Sci. 2023 Jun 30;24(13):10973. doi: 10.3390/ijms241310973 (PMC10341771; doi:10.3390/ijms241310973)
Supplement: Supplementary file 1 [file ijms-24-10973-s001.zip › Amino acids 6 m brain/20201001_001WT54_Method Report.pdf]

Biochrom 30+ Final Test

Method: C:\Biochrom\OpenLAB Projects\Default\Method\20180828mod.met
Standard: C:\Biochrom\OpenLAB Projects\Default\Result\20201001_001WT54.dat
Date : 10/7/2020 10:07:38 AM (GMT +02:00)

Instrument Serial No : 133260
Column No : H-0795
Resin No : 132-56

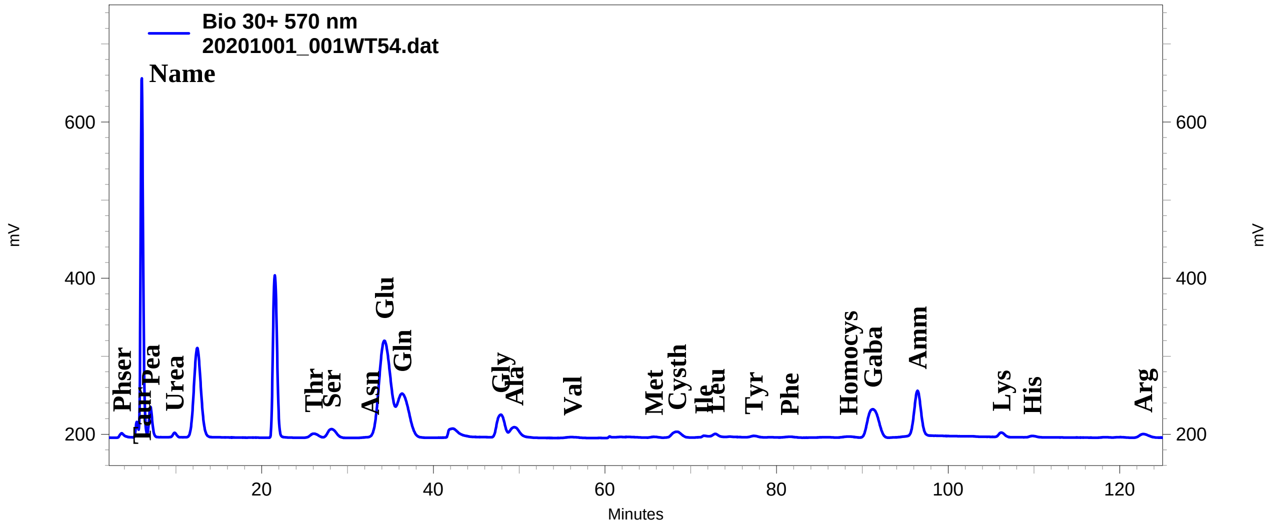

Bio 30+ 570 nm
Results

Pk #	Name	Retention Time	Area	ESTD concentration	Units
1	Phser	3.700	18575320	12.924	µmol/L
3	Taur	6.033	940833166	831.414	µmol/L
4	Pea	7.033	104727959	126.695	µmol/L
5	Urea	9.833	16061702	421.594	µmol/L
	Asp			0.000 BDL	µmol/L
8	Thr	26.067	32979378	25.692	µmol/L
9	Ser	28.133	76439242	58.837	µmol/L
10	Asn	32.633	3604045	4.614	µmol/L
11	Glu	34.300	1177249271	931.584	µmol/L
12	Gln	36.367	533347899	421.197	µmol/L
	Sarc			0.000 BDL	µmol/L
	AAAA			0.000 BDL	µmol/L
14	Gly	47.867	182834879	132.820	µmol/L
15	Ala	49.433	100345080	79.338	µmol/L
	Citr			0.000 BDL	µmol/L
	Aaba			0.000 BDL	µmol/L
16	Val	56.233	7728372	6.386	µmol/L
	Cys			0.000 BDL	µmol/L
18	Met	65.700	5564010	4.315	µmol/L
19	Cysth	68.400	57362389	41.528	µmol/L
20	Ile	71.533	6946522	5.501	µmol/L
21	Leu	72.900	19584554	14.666	µmol/L
	Nleu			0.000 BDL	µmol/L
22	Tyr	77.333	9778040	7.810	µmol/L
	B-ala			0.000 BDL	µmol/L
23	Phe	81.500	5923754	4.644	µmol/L
	Baiba			0.000 BDL	µmol/L
24	Homocys	88.367	9823846	3.928	µmol/L
25	Gaba	91.233	323806348	324.607	µmol/L
	Ethan			0.000 BDL	µmol/L
26	Amm	96.433	329489640	244.014	µmol/L
	Hylys			0.000 BDL	µmol/L
	Orn			0.000 BDL	µmol/L
27	Lys	106.200	25941743	19.138	µmol/L
	1-Mhis			0.000 BDL	µmol/L
28	His	109.800	9120982	6.447	µmol/L
	Trp			0.000 BDL	µmol/L
	3-Mhis			0.000 BDL	µmol/L
	Ans			0.000 BDL	µmol/L
	Car			0.000 BDL	µmol/L
29	Arg	122.733	34676220	28.017	µmol/L

Totals			4032744361	3757.712	

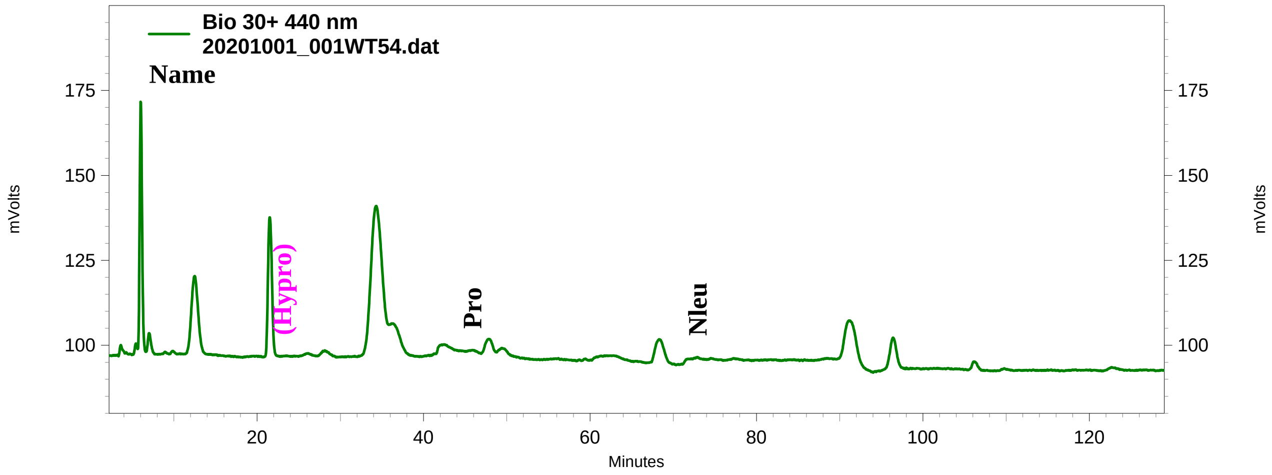

Bio 30+ 440 nm
Results

Pk #	Name	Retention Time	Area	ESTD concentration	Units
	Hypro			0.000 BDL	µmol/L
16	Pro	45.867	3785246	8.211	µmol/L
22	Nleu	72.933	9330951	32.625	µmol/L

Totals			13116197	40.836	
