# Supplementary material for: Pathophysiology of Cerebellar Degeneration in Mitochondrial Disorders: Insights from the Harlequin Mouse
Source: Int J Mol Sci. 2023 Jun 30;24(13):10973. doi: 10.3390/ijms241310973 (PMC10341771; doi:10.3390/ijms241310973)
Supplement: Supplementary file 1 [file ijms-24-10973-s001.zip › Amino acids 6 m cerebellum/20200324_001HQ-4-19_Method Report.pdf]

Biochrom 30+ Final Test

Method: C:\Biochrom\OpenLAB Projects\Default\Method\20180828mod.met
Standard: C:\Biochrom\OpenLAB Projects\Default\Result\20200324_001HQ-4-19.dat
Date : 4/1/2020 10:20:51 AM (GMT +02:00)

Instrument Serial No : 133260
Column No : H-0795
Resin No : 132-56

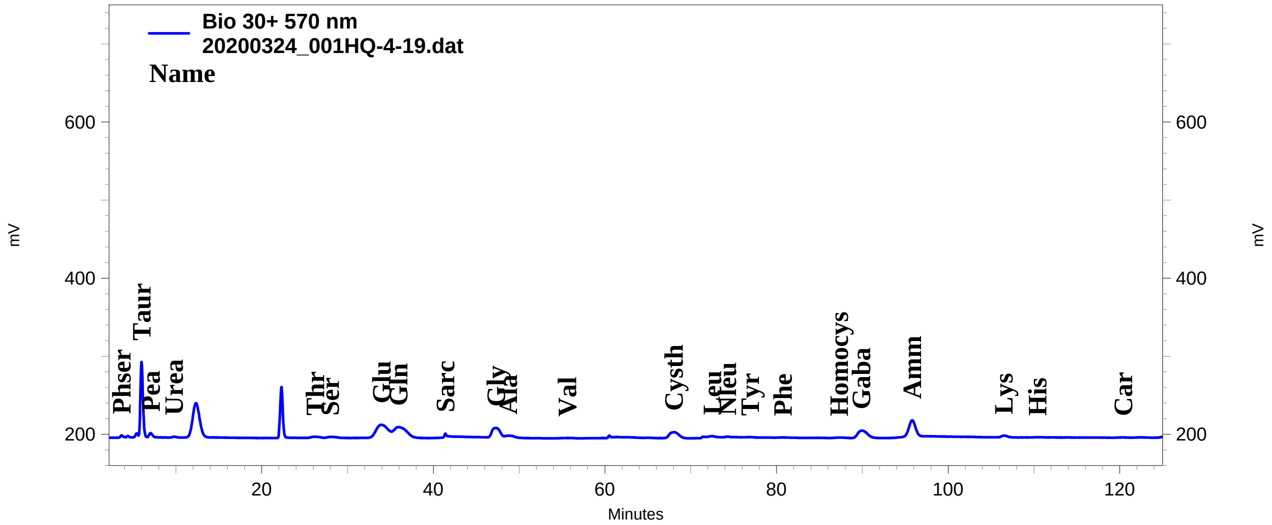

Bio 30+ 570 nm
Results

Pk #	Name	Retention Time	Area	ESTD concentration	Units
1	Phser	3.667	6942977	4.831	µmol/L
4	Taur	6.000	200151997	176.874	µmol/L
5	Pea	7.067	17309810	20.941	µmol/L
6	Urea	9.767	3319831	87.140	µmol/L
	Asp			0.000 BDL	µmol/L
9	Thr	26.233	9025729	7.031	µmol/L
10	Ser	27.933	9580869	7.375	µmol/L
	Asn			0.000 BDL	µmol/L
11	Glu	33.967	158345978	125.303	µmol/L
12	Gln	35.933	145943966	115.255	µmol/L
13	Sarc	41.400	11677886	72.874	µmol/L
	AAAA			0.000 BDL	µmol/L
14	Gly	47.300	80158915	58.231	µmol/L
15	Ala	48.700	18676679	14.767	µmol/L
	Citr			0.000 BDL	µmol/L
	Aaba			0.000 BDL	µmol/L
16	Val	55.600	5722476	4.728	µmol/L
	Cys			0.000 BDL	µmol/L
	Met			0.000 BDL	µmol/L
18	Cysth	68.033	56166364	40.662	µmol/L
	Ile			0.000 BDL	µmol/L
19	Leu	72.500	18917331	14.167	µmol/L
20	Nleu	74.233	3738498	0.000	µmol/L
21	Tyr	76.900	2528130	2.019	µmol/L
	B-ala			0.000 BDL	µmol/L
22	Phe	80.733	1931284	1.514	µmol/L
	Baiba			0.000 BDL	µmol/L
23	Homocys	87.267	5741184	2.296	µmol/L
24	Gaba	89.867	73533060	73.715	µmol/L
	Ethan			0.000 BDL	µmol/L
25	Amm	95.800	117517954	87.032	µmol/L
	Hylys			0.000 BDL	µmol/L
	Orn			0.000 BDL	µmol/L
26	Lys	106.467	9631358	7.105	µmol/L
	1-Mhis			0.000 BDL	µmol/L
27	His	110.400	2542402	1.797	µmol/L
	Trp			0.000 BDL	µmol/L
	3-Mhis			0.000 BDL	µmol/L
	Ans			0.000 BDL	µmol/L
28	Car	120.400	2149206	3.762	µmol/L
29	Arg	125.400	8518312	6.883	µmol/L
Totals			969772196	936.302	

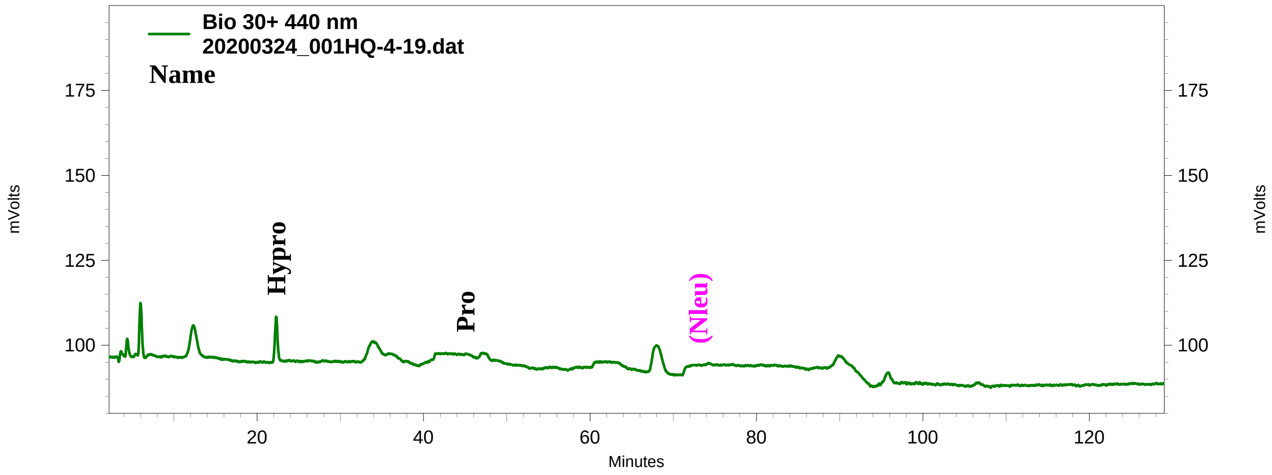

Bio 30+ 440 nm
Results

Pk #	Name	Retention Time	Area	ESTD concentration	Units
8	Hypro	22.300	30149656	120.340	µmol/L
12	Pro	45.033	2247340	4.875	µmol/L
	Nleu			0.000 BDL	µmol/L

Totals			32396996	125.214	
