# Supplementary material for: Pathophysiology of Cerebellar Degeneration in Mitochondrial Disorders: Insights from the Harlequin Mouse
Source: Int J Mol Sci. 2023 Jun 30;24(13):10973. doi: 10.3390/ijms241310973 (PMC10341771; doi:10.3390/ijms241310973)
Supplement: Supplementary file 1 [file ijms-24-10973-s001.zip › Amino acids 6 m cerebellum/20200324_001HQ-4-61_Method Report.pdf]

Biochrom 30+ Final Test

Method: C:\Biochrom\OpenLAB Projects\Default\Method\20180828mod.met
Standard: C:\Biochrom\OpenLAB Projects\Default\Result\20200324_001HQ-4-61.dat
Date : 4/1/2020 10:24:15 AM (GMT +02:00)

Instrument Serial No : 133260
Column No : H-0795
Resin No : 132-56

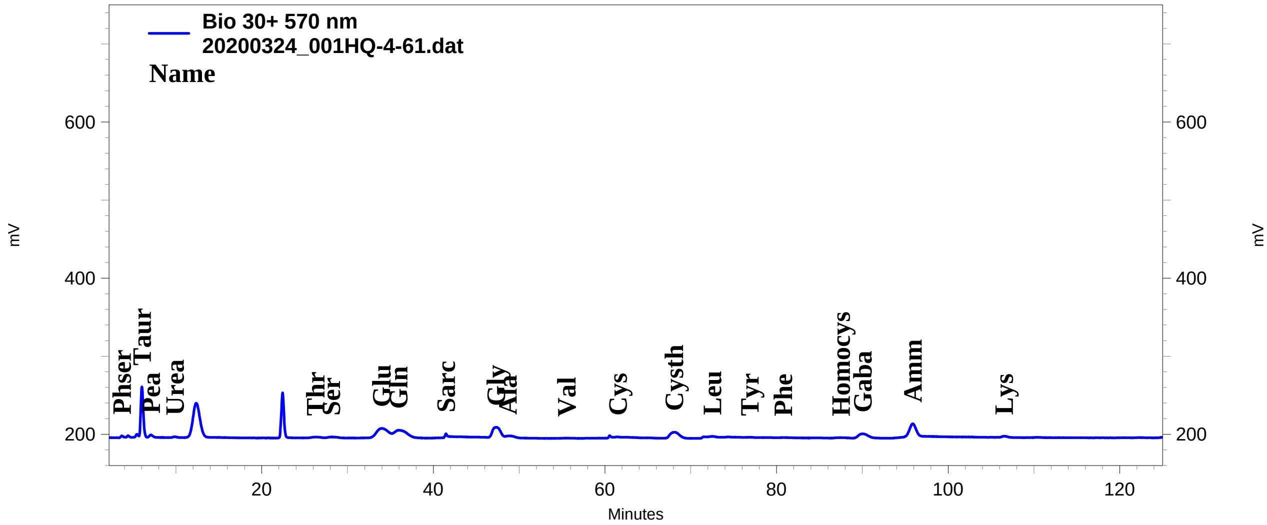

Bio 30+ 570 nm
Results

Pk #	Name	Retention Time	Area	ESTD concentration	Units
1	Phser	3.700	5612588	3.905	µmol/L
4	Taur	6.033	133092378	117.614	µmol/L
5	Pea	7.100	9524540	11.522	µmol/L
6	Urea	9.867	3206250	84.159	µmol/L
	Asp			0.000 BDL	µmol/L
9	Thr	26.267	6088255	4.743	µmol/L
10	Ser	28.067	9155006	7.047	µmol/L
	Asn			0.000 BDL	µmol/L
11	Glu	33.967	114012093	90.220	µmol/L
12	Gln	35.933	101598215	80.234	µmol/L
13	Sarc	41.467	10818522	67.511	µmol/L
	AAAA			0.000 BDL	µmol/L
14	Gly	47.300	84888815	61.667	µmol/L
15	Ala	48.667	16351383	12.928	µmol/L
	Citr			0.000 BDL	µmol/L
	Aaba			0.000 BDL	µmol/L
16	Val	55.533	2711326	2.240	µmol/L
18	Cys	61.500	3911970	2.659	µmol/L
	Met			0.000 BDL	µmol/L
19	Cysth	68.067	56365818	40.806	µmol/L
	Ile			0.000 BDL	µmol/L
20	Leu	72.500	15463207	11.580	µmol/L
	Nleu			0.000 BDL	µmol/L
21	Tyr	76.867	1415508	1.131	µmol/L
	B-ala			0.000 BDL	µmol/L
22	Phe	80.767	1041394	0.816	µmol/L
	Baiba			0.000 BDL	µmol/L
23	Homocys	87.500	5434992	2.173	µmol/L
24	Gaba	90.033	40632425	40.733	µmol/L
	Ethan			0.000 BDL	µmol/L
25	Amm	95.867	94350346	69.874	µmol/L
	Hylys			0.000 BDL	µmol/L
	Orn			0.000 BDL	µmol/L
26	Lys	106.500	7199985	5.312	µmol/L
	1-Mhis			0.000 BDL	µmol/L
	His			0.000 BDL	µmol/L
	Trp			0.000 BDL	µmol/L
	3-Mhis			0.000 BDL	µmol/L
	Ans			0.000 BDL	µmol/L
	Car			0.000 BDL	µmol/L
27	Arg	125.433	6849878	5.534	µmol/L

Totals			729724894	724.410	

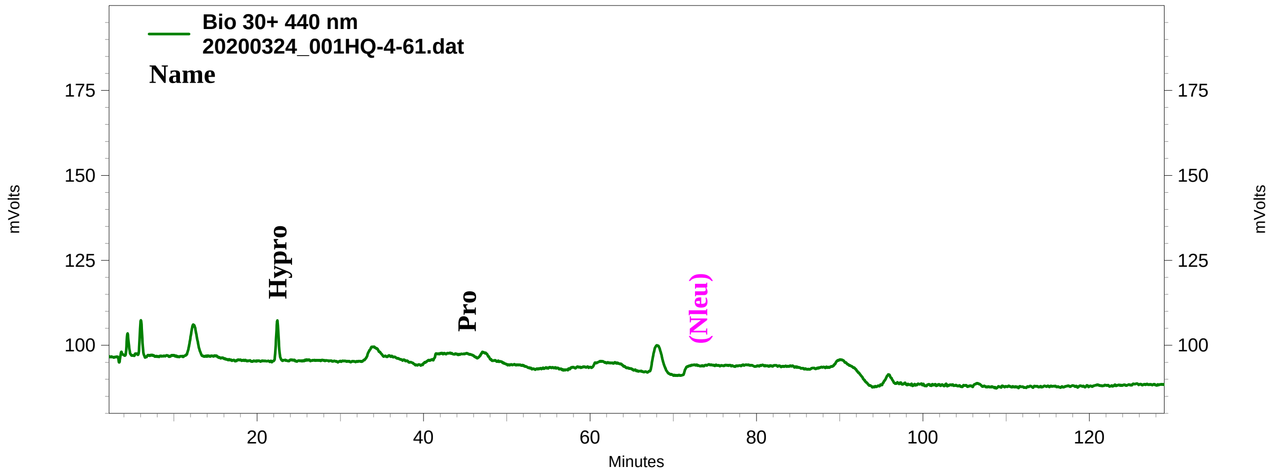

Bio 30+ 440 nm
Results

Pk #	Name	Retention Time	Area	ESTD concentration	Units
7	Hypro	22.433	26038751	103.931	µmol/L
14	Pro	45.200	4742153	10.286	µmol/L
	Nleu			0.000 BDL	µmol/L
Totals			30780904	114.218	
