# Supplementary material for: Pathophysiology of Cerebellar Degeneration in Mitochondrial Disorders: Insights from the Harlequin Mouse
Source: Int J Mol Sci. 2023 Jun 30;24(13):10973. doi: 10.3390/ijms241310973 (PMC10341771; doi:10.3390/ijms241310973)
Supplement: Supplementary file 1 [file ijms-24-10973-s001.zip › Amino acids 6 m cerebellum/20200324_001HQ-55_Method Report.pdf]

Biochrom 30+ Final Test

Method: C:\Biochrom\OpenLAB Projects\Default\Method\20180828mod.met
Standard: C:\Biochrom\OpenLAB Projects\Default\Result\20200324_001HQ-55.dat
Date : 4/1/2020 10:22:38 AM (GMT +02:00)

Instrument Serial No : 133260
Column No : H-0795
Resin No : 132-56

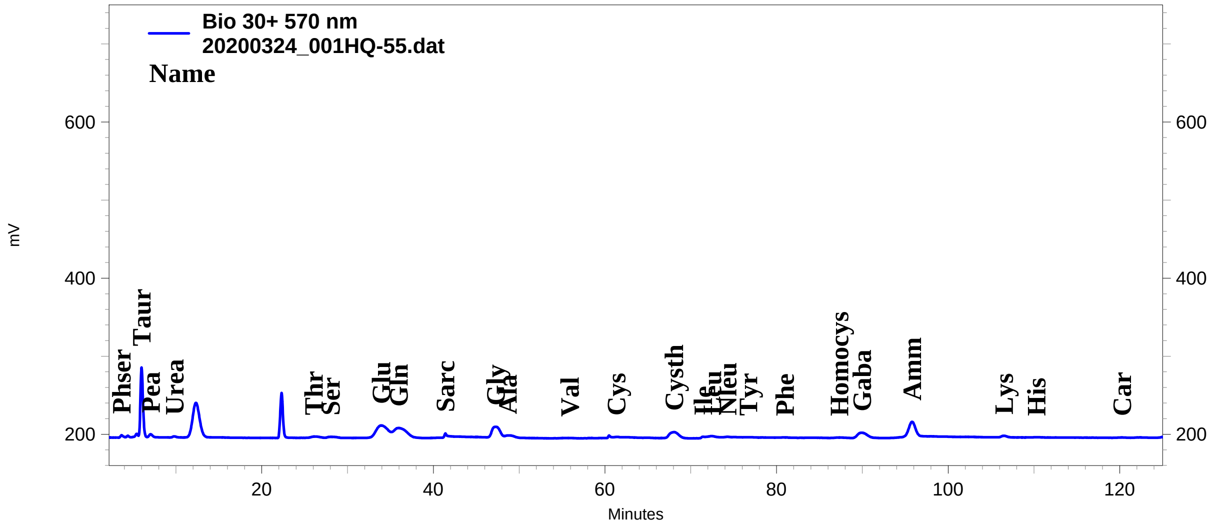

Bio 30+ 570 nm
Results

Pk #	Name	Retention Time	Area	ESTD concentration	Units
1	Phser	3.667	7002391	4.872	µmol/L
4	Taur	6.000	184769743	163.281	µmol/L
5	Pea	7.067	12540153	15.171	µmol/L
6	Urea	9.833	4214576	110.626	µmol/L
	Asp			0.000 BDL	µmol/L
9	Thr	26.067	9758554	7.602	µmol/L
10	Ser	28.033	9329445	7.181	µmol/L
	Asn			0.000 BDL	µmol/L
11	Glu	33.933	150239564	118.888	µmol/L
12	Gln	35.967	136304576	107.643	µmol/L
13	Sarc	41.400	13096550	81.727	µmol/L
	AAAA			0.000 BDL	µmol/L
14	Gly	47.267	89362421	64.917	µmol/L
15	Ala	48.667	20677807	16.349	µmol/L
	Citr			0.000 BDL	µmol/L
	Aaba			0.000 BDL	µmol/L
16	Val	55.900	3690016	3.049	µmol/L
18	Cys	61.333	2827143	1.921	µmol/L
	Met			0.000 BDL	µmol/L
19	Cysth	68.067	56435225	40.856	µmol/L
20	Ile	71.433	3987725	3.158	µmol/L
21	Leu	72.500	15950755	11.945	µmol/L
22	Nleu	74.267	3494701	0.000	µmol/L
23	Tyr	76.700	1240108	0.991	µmol/L
	B-ala			0.000 BDL	µmol/L
24	Phe	80.967	1223442	0.959	µmol/L
	Baiba			0.000 BDL	µmol/L
25	Homocys	87.300	4726437	1.890	µmol/L
26	Gaba	89.967	51902858	52.031	µmol/L
	Ethan			0.000 BDL	µmol/L
27	Amm	95.800	108743032	80.533	µmol/L
	Hylys			0.000 BDL	µmol/L
	Orn			0.000 BDL	µmol/L
28	Lys	106.500	7230020	5.334	µmol/L
	1-Mhis			0.000 BDL	µmol/L
29	His	110.300	1705337	1.205	µmol/L
	Trp			0.000 BDL	µmol/L
	3-Mhis			0.000 BDL	µmol/L
	Ans			0.000 BDL	µmol/L
30	Car	120.267	2256206	3.949	µmol/L
31	Arg	125.300	6270274	5.066	µmol/L

Totals			908979059	911.146	

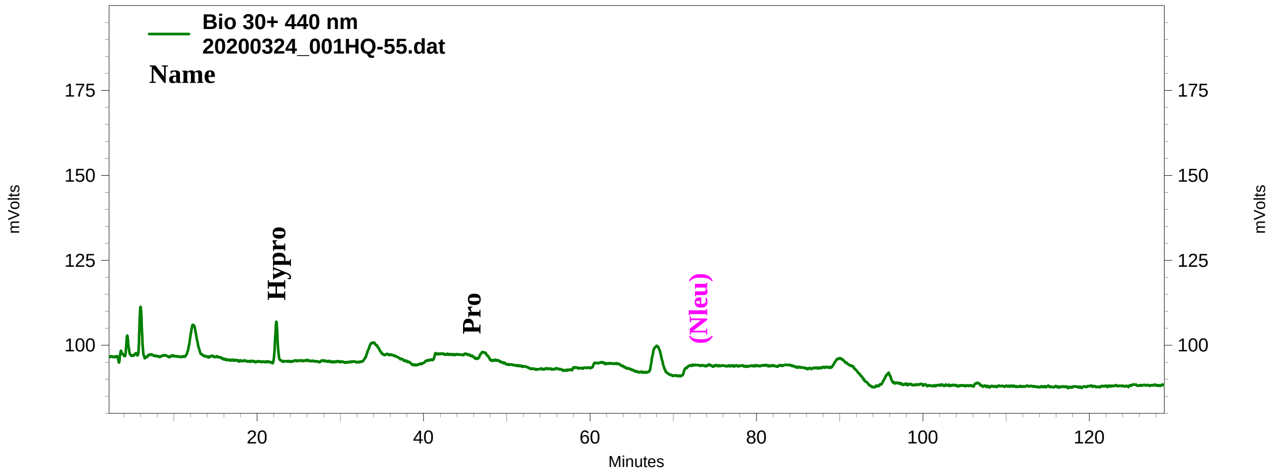

Bio 30+ 440 nm
Results

Pk #	Name	Retention Time	Area	ESTD concentration	Units
10	Hypro	22.300	26597484	106.162	µmol/L
16	Pro	45.767	1146038	2.486	µmol/L
	Nleu			0.000 BDL	µmol/L

Totals			27743522	108.647	
