# Supplementary material for: Pathophysiology of Cerebellar Degeneration in Mitochondrial Disorders: Insights from the Harlequin Mouse
Source: Int J Mol Sci. 2023 Jun 30;24(13):10973. doi: 10.3390/ijms241310973 (PMC10341771; doi:10.3390/ijms241310973)
Supplement: Supplementary file 1 [file ijms-24-10973-s001.zip › Amino acids 6 m cerebellum/20200324_001HQ-76_Method Report.pdf]

Biochrom 30+ Final Test

Method: C:\Biochrom\OpenLAB Projects\Default\Method\20180828mod.met
Standard: C:\Biochrom\OpenLAB Projects\Default\Result\20200324_001HQ-76.dat
Date : 4/1/2020 10:28:44 AM (GMT +02:00)

Instrument Serial No : 133260
Column No : H-0795
Resin No : 132-56

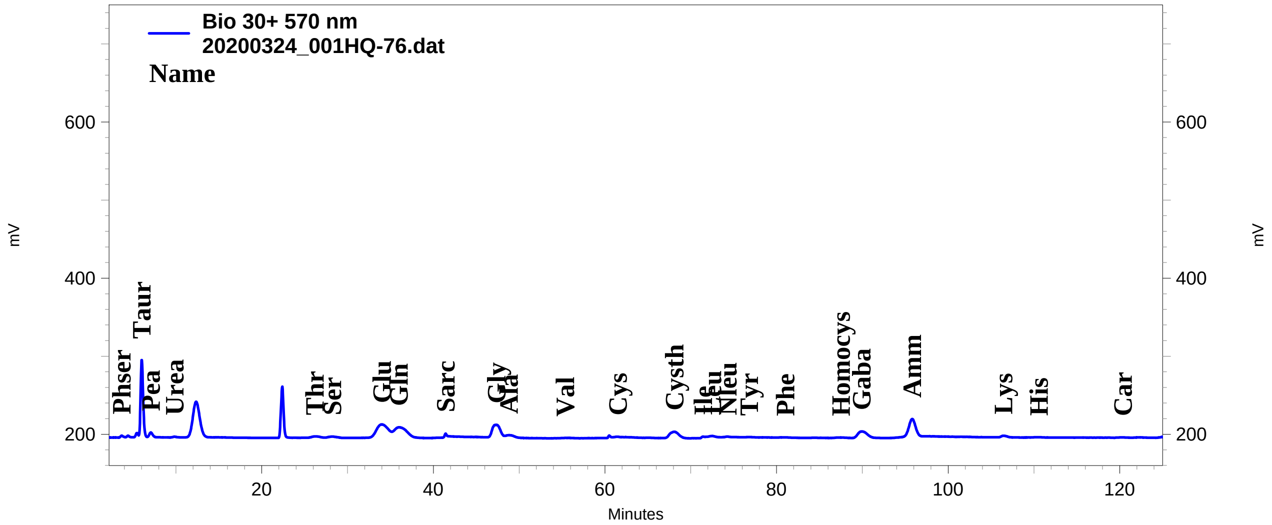

Bio 30+ 570 nm
Results

Pk #	Name	Retention Time	Area	ESTD concentration	Units
1	Phser	3.667	5539443	3.854	µmol/L
4	Taur	6.000	204074570	180.341	µmol/L
5	Pea	7.067	17698595	21.411	µmol/L
6	Urea	9.833	2984213	78.331	µmol/L
	Asp			0.000 BDL	µmol/L
9	Thr	26.167	10576192	8.239	µmol/L
10	Ser	28.200	10138552	7.804	µmol/L
	Asn			0.000 BDL	µmol/L
11	Glu	34.000	161413400	127.730	µmol/L
12	Gln	35.967	139753208	110.366	µmol/L
13	Sarc	41.433	11933690	74.470	µmol/L
	AAAA			0.000 BDL	µmol/L
14	Gly	47.367	104973759	76.258	µmol/L
15	Ala	48.800	21694729	17.153	µmol/L
	Citr			0.000 BDL	µmol/L
	Aaba			0.000 BDL	µmol/L
16	Val	55.367	4893250	4.043	µmol/L
18	Cys	61.500	3746870	2.547	µmol/L
	Met			0.000 BDL	µmol/L
19	Cysth	68.100	58137896	42.089	µmol/L
20	Ile	71.433	3997549	3.166	µmol/L
21	Leu	72.500	15078263	11.292	µmol/L
22	Nleu	74.300	3932036	0.000	µmol/L
23	Tyr	76.800	1716874	1.371	µmol/L
	B-ala			0.000 BDL	µmol/L
24	Phe	81.033	3134272	2.457	µmol/L
	Baiba			0.000 BDL	µmol/L
25	Homocys	87.500	4461960	1.784	µmol/L
26	Gaba	89.900	62026657	62.180	µmol/L
	Ethan			0.000 BDL	µmol/L
27	Amm	95.800	127014300	94.065	µmol/L
	Hylys			0.000 BDL	µmol/L
	Orn			0.000 BDL	µmol/L
28	Lys	106.433	8995708	6.636	µmol/L
	1-Mhis			0.000 BDL	µmol/L
29	His	110.567	2722838	1.925	µmol/L
	Trp			0.000 BDL	µmol/L
	3-Mhis			0.000 BDL	µmol/L
	Ans			0.000 BDL	µmol/L
30	Car	120.367	2767934	4.845	µmol/L
31	Arg	125.367	9262910	7.484	µmol/L

Totals			1002669668	951.842	

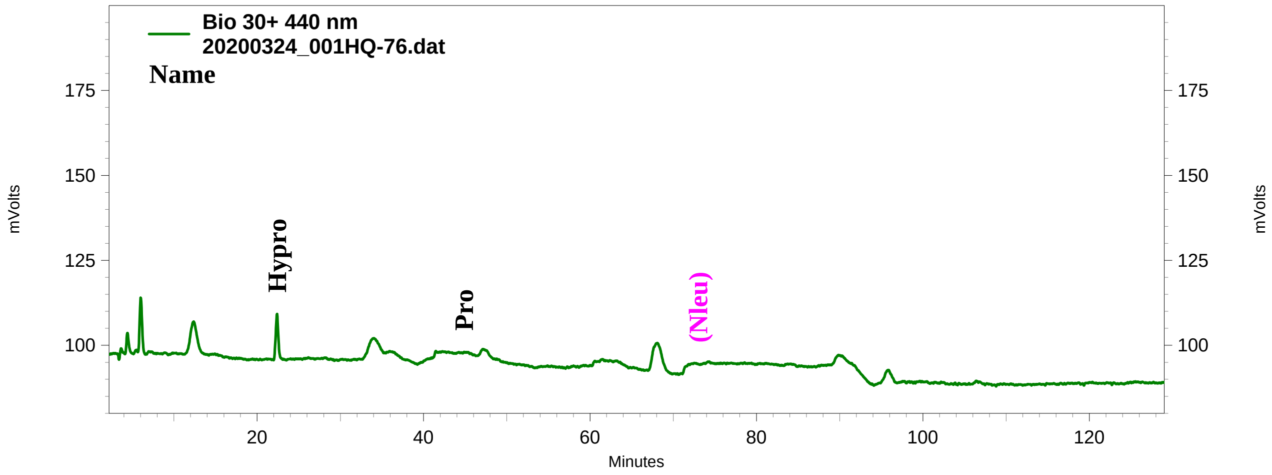

Bio 30+ 440 nm
Results

Pk #	Name	Retention Time	Area	ESTD concentration	Units
13	Hypro	22.400	30792328	122.905	µmol/L
19	Pro	44.867	1037704	2.251	µmol/L
	Nleu			0.000 BDL	µmol/L

Totals			31830032	125.156	
