# Supplementary material for: Pathophysiology of Cerebellar Degeneration in Mitochondrial Disorders: Insights from the Harlequin Mouse
Source: Int J Mol Sci. 2023 Jun 30;24(13):10973. doi: 10.3390/ijms241310973 (PMC10341771; doi:10.3390/ijms241310973)
Supplement: Supplementary file 1 [file ijms-24-10973-s001.zip › Amino acids 6 m cerebellum/20200324_001HQ-8-93_Method Report.pdf]

Biochrom 30+ Final Test

Method: C:\Biochrom\OpenLAB Projects\Default\Method\20180828mod.met
Standard: C:\Biochrom\OpenLAB Projects\Default\Result\20200324_001HQ-8-93.dat
Date : 4/1/2020 10:30:05 AM (GMT +02:00)

Instrument Serial No : 133260
Column No : H-0795
Resin No : 132-56

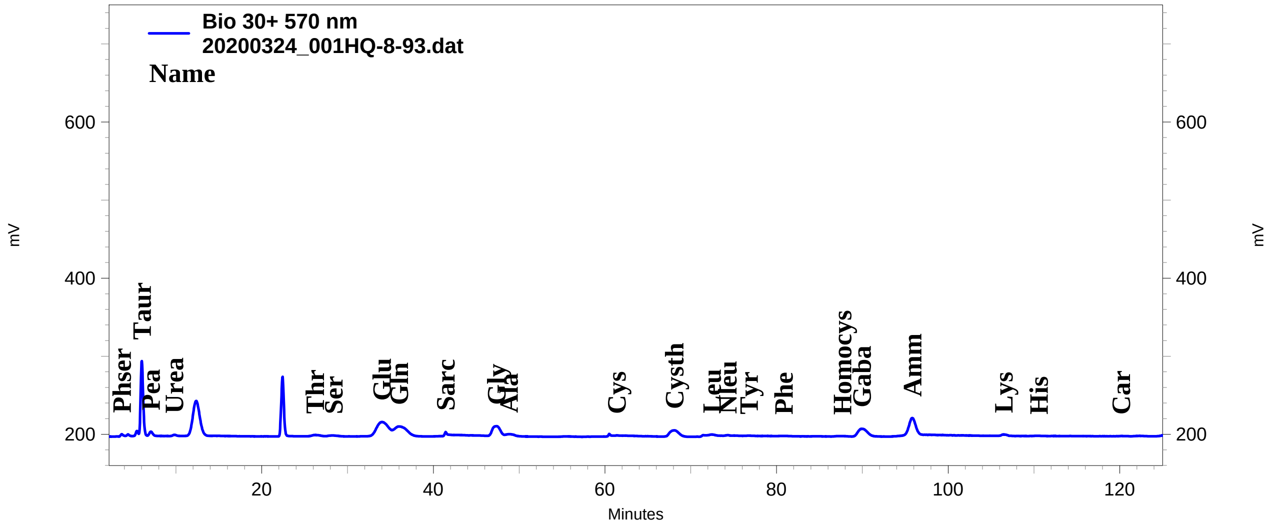

Bio 30+ 570 nm
Results

Pk #	Name	Retention Time	Area	ESTD concentration	Units
1	Phser	3.700	6561634	4.565	µmol/L
4	Taur	6.033	198273085	175.214	µmol/L
5	Pea	7.067	16372431	19.807	µmol/L
6	Urea	9.800	4324038	113.499	µmol/L
	Asp			0.000 BDL	µmol/L
9	Thr	26.200	9954548	7.755	µmol/L
10	Ser	28.367	8714220	6.707	µmol/L
	Asn			0.000 BDL	µmol/L
11	Glu	34.000	173488678	137.286	µmol/L
12	Gln	36.000	129932952	102.611	µmol/L
13	Sarc	41.433	10660708	66.526	µmol/L
	AAAA			0.000 BDL	µmol/L
14	Gly	47.367	80805400	58.701	µmol/L
15	Ala	48.800	17796829	14.071	µmol/L
	Citr			0.000 BDL	µmol/L
	Aaba			0.000 BDL	µmol/L
	Val			0.000 BDL	µmol/L
17	Cys	61.367	2576466	1.751	µmol/L
	Met			0.000 BDL	µmol/L
18	Cysth	68.100	58325628	42.225	µmol/L
	Ile			0.000 BDL	µmol/L
19	Leu	72.467	15752997	11.797	µmol/L
20	Nleu	74.300	1412974	0.000	µmol/L
21	Tyr	76.767	2394926	1.913	µmol/L
	B-ala			0.000 BDL	µmol/L
22	Phe	80.833	1249200	0.979	µmol/L
	Baiba			0.000 BDL	µmol/L
23	Homocys	87.667	5462266	2.184	µmol/L
24	Gaba	89.933	73705792	73.888	µmol/L
	Ethan			0.000 BDL	µmol/L
25	Amm	95.833	123844987	91.717	µmol/L
	Hylys			0.000 BDL	µmol/L
	Orn			0.000 BDL	µmol/L
26	Lys	106.467	7976494	5.885	µmol/L
	1-Mhis			0.000 BDL	µmol/L
27	His	110.567	2029538	1.435	µmol/L
	Trp			0.000 BDL	µmol/L
	3-Mhis			0.000 BDL	µmol/L
	Ans			0.000 BDL	µmol/L
28	Car	120.167	1212526	2.122	µmol/L
29	Arg	125.367	8773840	7.089	µmol/L
Totals			961602157	949.728	

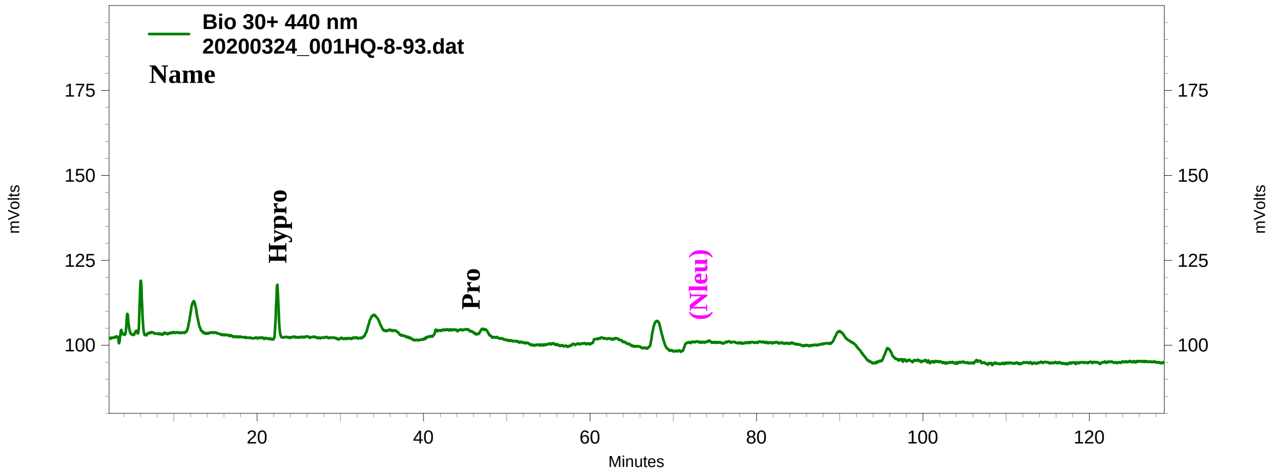

Bio 30+ 440 nm
Results

Pk #	Name	Retention Time	Area	ESTD concentration	Units
10	Hypro	22.433	34500451	137.706	µmol/L
13	Pro	45.667	669519	1.452	µmol/L
	Nleu			0.000 BDL	µmol/L

Totals			35169970	139.158	
