# Supplementary material for: Pathophysiology of Cerebellar Degeneration in Mitochondrial Disorders: Insights from the Harlequin Mouse
Source: Int J Mol Sci. 2023 Jun 30;24(13):10973. doi: 10.3390/ijms241310973 (PMC10341771; doi:10.3390/ijms241310973)
Supplement: Supplementary file 1 [file ijms-24-10973-s001.zip › Amino acids 6 m cerebellum/20200324_001WT-3-23_Method Report.pdf]

Biochrom 30+ Final Test

Method: C:\Biochrom\OpenLAB Projects\Default\Method\20180828mod.met
Standard: C:\Biochrom\OpenLAB Projects\Default\Result\20200324_001WT-3-23.dat
Date : 4/1/2020 9:19:44 AM (GMT +02:00)

Instrument Serial No : 133260
Column No : H-0795
Resin No : 132-56

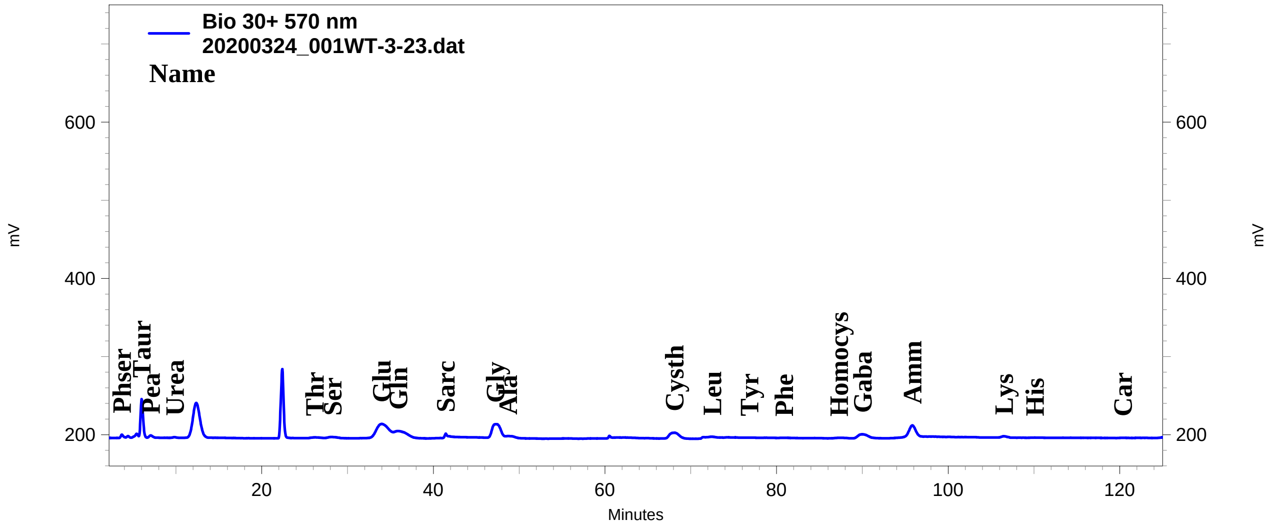

Bio 30+ 570 nm
Results

Pk #	Name	Retention Time	Area	ESTD concentration	Units
1	Phser	3.700	9202128	6.402	µmol/L
4	Taur	6.000	103480128	91.445	µmol/L
5	Pea	7.067	9193698	11.122	µmol/L
6	Urea	9.867	2394428	62.850	µmol/L
	Asp			0.000 BDL	µmol/L
9	Thr	26.167	5954737	4.639	µmol/L
10	Ser	28.233	10334009	7.954	µmol/L
	Asn			0.000 BDL	µmol/L
11	Glu	33.967	182340646	144.290	µmol/L
12	Gln	35.900	93594048	73.913	µmol/L
13	Sarc	41.433	14905264	93.014	µmol/L
	AAAA			0.000 BDL	µmol/L
14	Gly	47.233	121553652	88.303	µmol/L
15	Ala	48.700	16731617	13.229	µmol/L
	Citr			0.000 BDL	µmol/L
	Aaba			0.000 BDL	µmol/L
	Val			0.000 BDL	µmol/L
	Cys			0.000 BDL	µmol/L
	Met			0.000 BDL	µmol/L
17	Cysth	68.100	56291650	40.752	µmol/L
	Ile			0.000 BDL	µmol/L
18	Leu	72.500	14717441	11.022	µmol/L
	Nleu			0.000 BDL	µmol/L
19	Tyr	76.833	820020	0.655	µmol/L
	B-ala			0.000 BDL	µmol/L
20	Phe	80.867	536622	0.421	µmol/L
	Baiba			0.000 BDL	µmol/L
21	Homocys	87.267	4765202	1.906	µmol/L
22	Gaba	90.033	39827172	39.926	µmol/L
	Ethan			0.000 BDL	µmol/L
23	Amm	95.833	84458945	62.549	µmol/L
	Hylys			0.000 BDL	µmol/L
	Orn			0.000 BDL	µmol/L
24	Lys	106.500	7570570	5.585	µmol/L
	1-Mhis			0.000 BDL	µmol/L
25	His	110.100	2404398	1.700	µmol/L
	Trp			0.000 BDL	µmol/L
	3-Mhis			0.000 BDL	µmol/L
	Ans			0.000 BDL	µmol/L
26	Car	120.367	1586516	2.777	µmol/L
27	Arg	125.333	8562872	6.919	µmol/L

Totals			791225763	771.372	

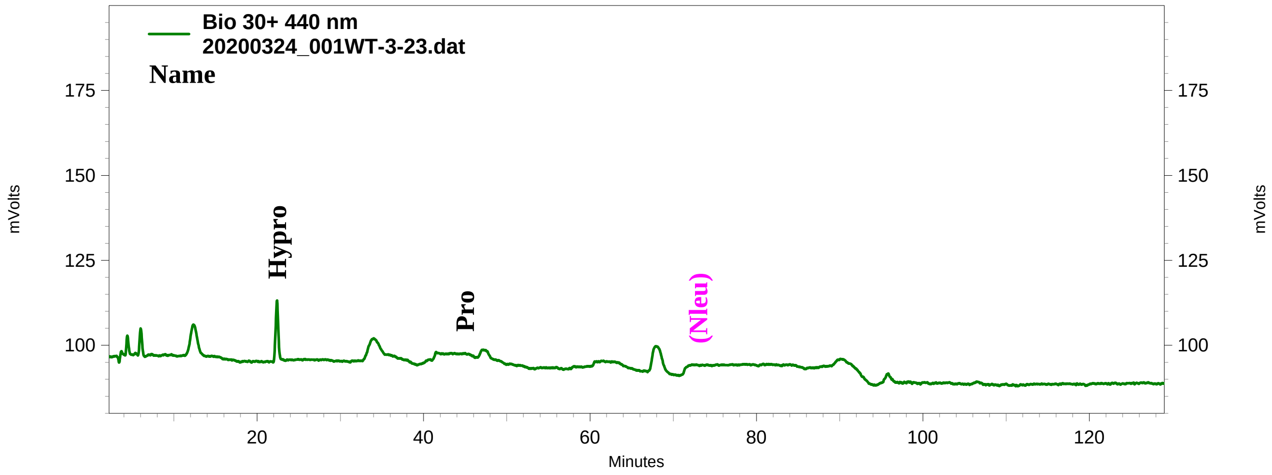

Bio 30+ 440 nm
Results

Pk #	Name	Retention Time	Area	ESTD concentration	Units
7	Hypro	22.400	41629846	166.162	µmol/L
11	Pro	44.967	3299864	7.158	µmol/L
	Nleu			0.000 BDL	µmol/L

Totals			44929710	173.320	
