# Supplementary material for: Pathophysiology of Cerebellar Degeneration in Mitochondrial Disorders: Insights from the Harlequin Mouse
Source: Int J Mol Sci. 2023 Jun 30;24(13):10973. doi: 10.3390/ijms241310973 (PMC10341771; doi:10.3390/ijms241310973)
Supplement: Supplementary file 1 [file ijms-24-10973-s001.zip › Amino acids 6 m cerebellum/20200324_001WT-3-25_Method Report.pdf]

Biochrom 30+ Final Test

Method: C:\Biochrom\OpenLAB Projects\Default\Method\20180828mod.met
Standard: C:\Biochrom\OpenLAB Projects\Default\Result\20200324_001WT-3-25.dat
Date : 4/1/2020 9:24:16 AM (GMT +02:00)

Instrument Serial No : 133260
Column No : H-0795
Resin No : 132-56

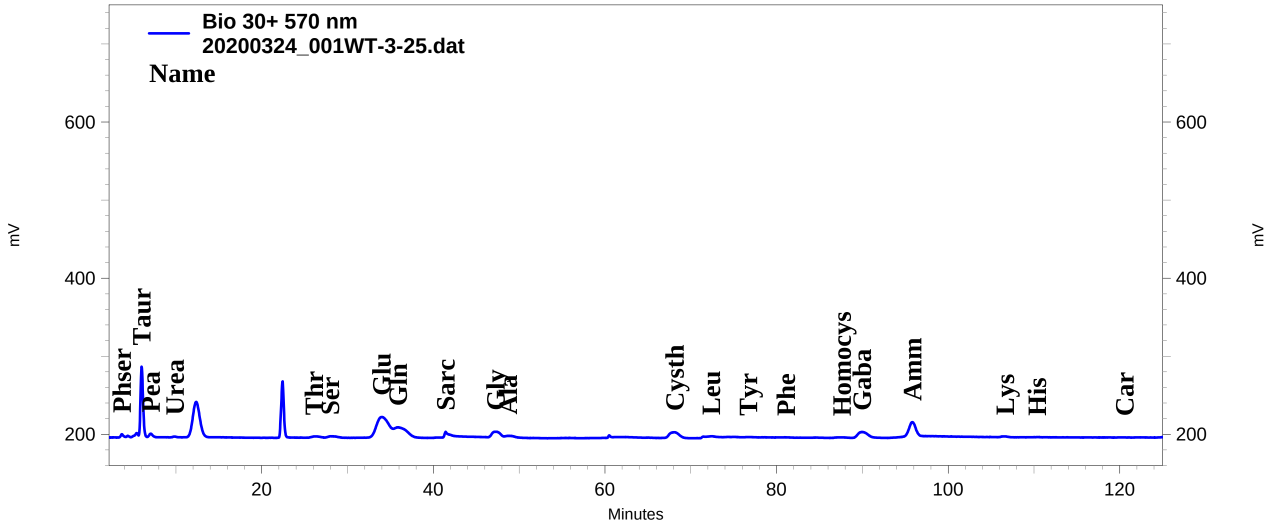

Bio 30+ 570 nm
Results

Pk #	Name	Retention Time	Area	ESTD concentration	Units
1	Phser	3.700	9009089	6.268	µmol/L
4	Taur	6.000	187668436	165.843	µmol/L
5	Pea	7.067	13749221	16.633	µmol/L
6	Urea	9.867	3175170	83.343	µmol/L
	Asp			0.000 BDL	µmol/L
9	Thr	26.100	10375180	8.083	µmol/L
10	Ser	27.967	13506174	10.396	µmol/L
	Asn			0.000 BDL	µmol/L
11	Glu	33.967	269148728	212.983	µmol/L
12	Gln	35.867	133240406	105.223	µmol/L
13	Sarc	41.433	27678343	172.722	µmol/L
	AAAA			0.000 BDL	µmol/L
14	Gly	47.267	47984593	34.858	µmol/L
15	Ala	48.700	16709887	13.212	µmol/L
	Citr			0.000 BDL	µmol/L
	Aaba			0.000 BDL	µmol/L
	Val			0.000 BDL	µmol/L
	Cys			0.000 BDL	µmol/L
	Met			0.000 BDL	µmol/L
17	Cysth	68.133	56357293	40.800	µmol/L
	Ile			0.000 BDL	µmol/L
18	Leu	72.367	15505818	11.612	µmol/L
	Nleu			0.000 BDL	µmol/L
19	Tyr	76.700	2395548	1.913	µmol/L
	B-ala			0.000 BDL	µmol/L
20	Phe	81.100	1064396	0.834	µmol/L
	Baiba			0.000 BDL	µmol/L
21	Homocys	87.600	5582750	2.232	µmol/L
22	Gaba	89.967	57896537	58.040	µmol/L
	Ethan			0.000 BDL	µmol/L
23	Amm	95.833	105756786	78.322	µmol/L
	Hylys			0.000 BDL	µmol/L
	Orn			0.000 BDL	µmol/L
24	Lys	106.633	4653285	3.433	µmol/L
	1-Mhis			0.000 BDL	µmol/L
25	His	110.367	2045978	1.446	µmol/L
	Trp			0.000 BDL	µmol/L
	3-Mhis			0.000 BDL	µmol/L
	Ans			0.000 BDL	µmol/L
26	Car	120.567	1098607	1.923	µmol/L
27	Arg	125.333	7314513	5.910	µmol/L

Totals			991916738	1036.030	

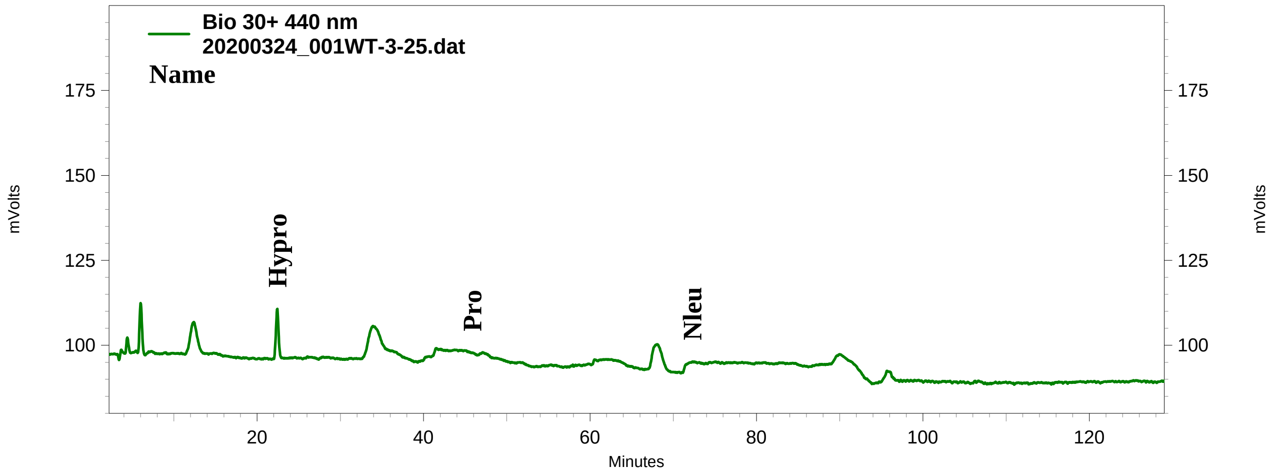

Bio 30+ 440 nm
Results

Pk #	Name	Retention Time	Area	ESTD concentration	Units
7	Hypro	22.433	32052248	127.934	µmol/L
10	Pro	45.867	562909	1.221	µmol/L
15	Nleu	72.300	12848137	44.923	µmol/L

Totals			45463294	174.078	
