# Supplementary material for: Pathophysiology of Cerebellar Degeneration in Mitochondrial Disorders: Insights from the Harlequin Mouse
Source: Int J Mol Sci. 2023 Jun 30;24(13):10973. doi: 10.3390/ijms241310973 (PMC10341771; doi:10.3390/ijms241310973)
Supplement: Supplementary file 1 [file ijms-24-10973-s001.zip › Amino acids 6 m cerebellum/20200324_001WT-4-14_Method Report.pdf]

Biochrom 30+ Final Test

Method: C:\Biochrom\OpenLAB Projects\Default\Method\20180828mod.met
Standard: C:\Biochrom\OpenLAB Projects\Default\Result\20200324_001WT-4-14.dat
Date : 4/1/2020 9:17:01 AM (GMT +02:00)

Instrument Serial No : 133260
Column No : H-0795
Resin No : 132-56

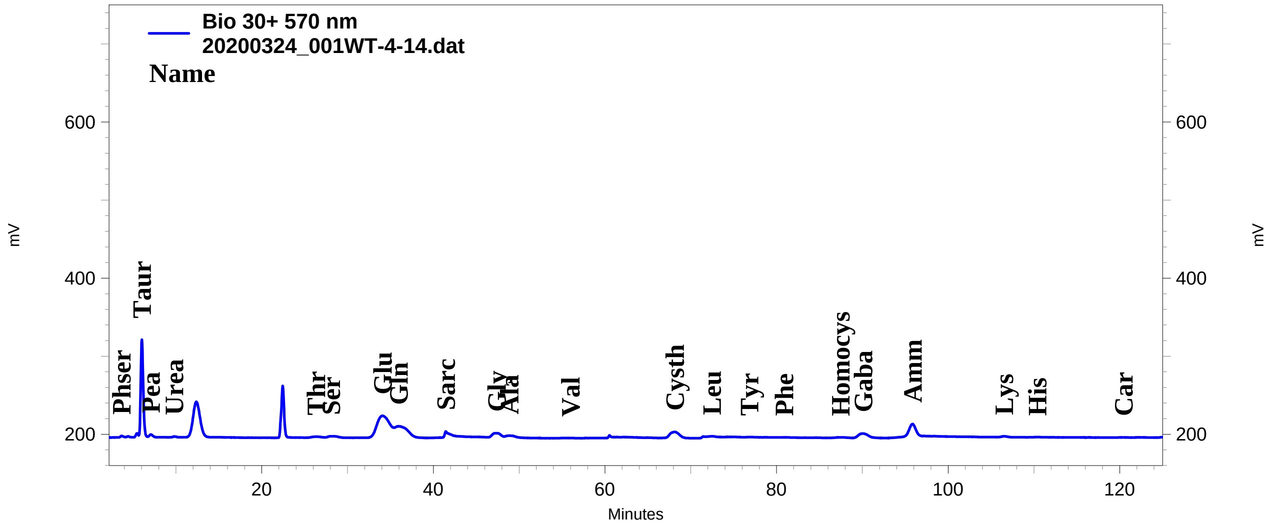

Bio 30+ 570 nm
Results

Pk #	Name	Retention Time	Area	ESTD concentration	Units
1	Phser	3.667	4847357	3.373	µmol/L
4	Taur	6.033	254091111	224.540	µmol/L
5	Pea	7.067	11094270	13.421	µmol/L
6	Urea	9.800	3077656	80.784	µmol/L
	Asp			0.000 BDL	µmol/L
9	Thr	26.333	8499863	6.622	µmol/L
10	Ser	28.067	14699787	11.315	µmol/L
	Asn			0.000 BDL	µmol/L
11	Glu	34.100	291922829	231.005	µmol/L
12	Gln	35.967	153293891	121.060	µmol/L
13	Sarc	41.467	33197976	207.166	µmol/L
	AAAA			0.000 BDL	µmol/L
14	Gly	47.400	35992662	26.147	µmol/L
15	Ala	48.867	18991192	15.015	µmol/L
	Citr			0.000 BDL	µmol/L
	Aaba			0.000 BDL	µmol/L
16	Val	56.000	3227784	2.667	µmol/L
	Cys			0.000 BDL	µmol/L
	Met			0.000 BDL	µmol/L
18	Cysth	68.167	56011382	40.550	µmol/L
	Ile			0.000 BDL	µmol/L
19	Leu	72.467	13946884	10.445	µmol/L
	Nleu			0.000 BDL	µmol/L
20	Tyr	76.833	2313182	1.848	µmol/L
	B-ala			0.000 BDL	µmol/L
21	Phe	80.900	1092856	0.857	µmol/L
	Baiba			0.000 BDL	µmol/L
22	Homocys	87.467	6217226	2.486	µmol/L
23	Gaba	90.100	43538562	43.646	µmol/L
	Ethan			0.000 BDL	µmol/L
24	Amm	95.867	89392848	66.203	µmol/L
	Hylys			0.000 BDL	µmol/L
	Orn			0.000 BDL	µmol/L
25	Lys	106.533	5498983	4.057	µmol/L
	1-Mhis			0.000 BDL	µmol/L
26	His	110.400	2130952	1.506	µmol/L
	Trp			0.000 BDL	µmol/L
	3-Mhis			0.000 BDL	µmol/L
	Ans			0.000 BDL	µmol/L
27	Car	120.467	1507464	2.639	µmol/L
28	Arg	125.333	6816478	5.508	µmol/L
Totals			1061403195	1122.858	

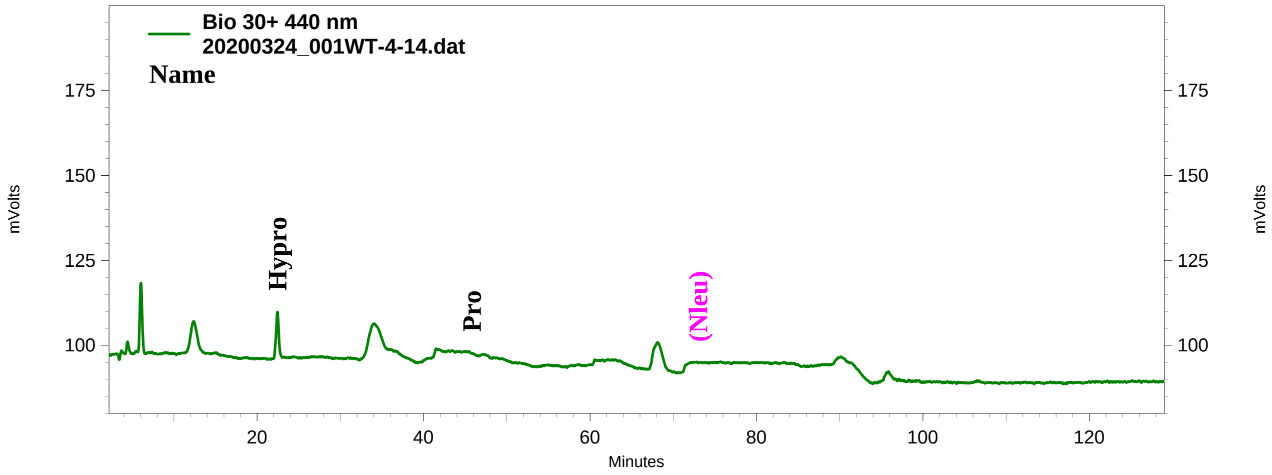

Bio 30+ 440 nm
Results

Pk #	Name	Retention Time	Area	ESTD concentration	Units
7	Hypro	22.433	31061894	123.981	µmol/L
13	Pro	45.800	605320	1.313	µmol/L
	Nleu			0.000 BDL	µmol/L
Totals			31667214	125.294	
