# Supplementary material for: Pathophysiology of Cerebellar Degeneration in Mitochondrial Disorders: Insights from the Harlequin Mouse
Source: Int J Mol Sci. 2023 Jun 30;24(13):10973. doi: 10.3390/ijms241310973 (PMC10341771; doi:10.3390/ijms241310973)
Supplement: Supplementary file 1 [file ijms-24-10973-s001.zip › Amino acids 6 m cerebellum/20200324_001WT-4-60_Method Report.pdf]

**Biochrom 30+ Final Test**

Method: C:\Biochrom\OpenLAB Projects\Default\Method\20180828mod.met
Standard: C:\Biochrom\OpenLAB Projects\Default\Result\20200324_001WT-4-60.dat
Date : 4/1/2020 9:40:50 AM (GMT +02:00)

Instrument Serial No : 133260
Column No : H-0795
Resin No : 132-56

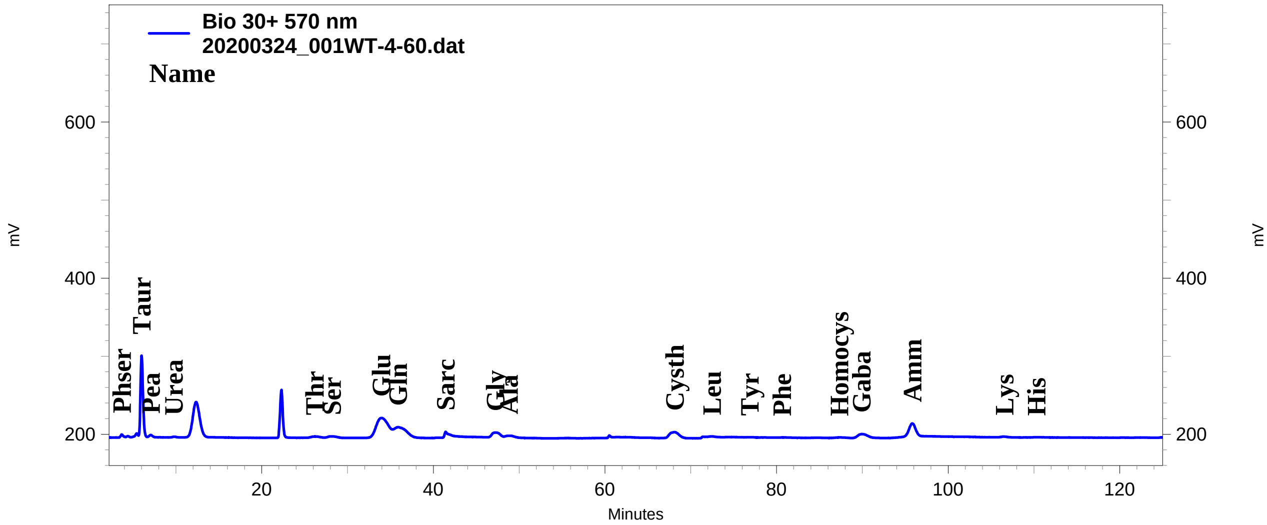


**Bio 30+ 570 nm**
**Results**

| Pk # | Name | Retention Time | Area | ESTD concentration | Units |
|---|---|---|---|---|---|
| 1 | Phser | 3.700 | 8272446 | 5.756 | µmol/L |
| 4 | Taur | 6.000 | 215948097 | 190.833 | µmol/L |
| 5 | Pea | 7.067 | 9140598 | 11.058 | µmol/L |
| 6 | Urea | 9.733 | 2627705 | 68.973 | µmol/L |
| | Asp | | | 0.000 BDL | µmol/L |
| 9 | Thr | 26.167 | 9656368 | 7.523 | µmol/L |
| 10 | Ser | 28.167 | 12771930 | 9.831 | µmol/L |
| | Asn | | | 0.000 BDL | µmol/L |
| 11 | Glu | 33.933 | 249706742 | 197.599 | µmol/L |
| 12 | Gln | 35.867 | 142674804 | 112.674 | µmol/L |
| 13 | Sarc | 41.433 | 29266030 | 182.630 | µmol/L |
| | AAAA | | | 0.000 BDL | µmol/L |
| 14 | Gly | 47.233 | 40312811 | 29.285 | µmol/L |
| 15 | Ala | 48.800 | 18203827 | 14.393 | µmol/L |
| | Citr | | | 0.000 BDL | µmol/L |
| | Aaba | | | 0.000 BDL | µmol/L |
| | Val | | | 0.000 BDL | µmol/L |
| | Cys | | | 0.000 BDL | µmol/L |
| | Met | | | 0.000 BDL | µmol/L |
| 17 | Cysth | 68.133 | 56797224 | 41.118 | µmol/L |
| | Ile | | | 0.000 BDL | µmol/L |
| 18 | Leu | 72.467 | 13650175 | 10.222 | µmol/L |
| | Nleu | | | 0.000 BDL | µmol/L |
| 19 | Tyr | 76.867 | 1715510 | 1.370 | µmol/L |
| | B-ala | | | 0.000 BDL | µmol/L |
| 20 | Phe | 80.633 | 1552936 | 1.217 | µmol/L |
| | Baiba | | | 0.000 BDL | µmol/L |
| 21 | Homocys | 87.300 | 5633851 | 2.253 | µmol/L |
| 22 | Gaba | 89.900 | 38262993 | 38.358 | µmol/L |
| | Ethan | | | 0.000 BDL | µmol/L |
| 23 | Amm | 95.833 | 94842670 | 70.239 | µmol/L |
| | Hylys | | | 0.000 BDL | µmol/L |
| | Orn | | | 0.000 BDL | µmol/L |
| 24 | Lys | 106.567 | 4386918 | 3.236 | µmol/L |
| | 1-Mhis | | | 0.000 BDL | µmol/L |
| 25 | His | 110.300 | 2829195 | 2.000 | µmol/L |
| | Trp | | | 0.000 BDL | µmol/L |
| | 3-Mhis | | | 0.000 BDL | µmol/L |
| | Ans | | | 0.000 BDL | µmol/L |
| | Car | | | 0.000 BDL | µmol/L |
| 26 | Arg | 125.367 | 6951444 | 5.617 | µmol/L |
| Totals | | | 965204274 | 1006.184 | |

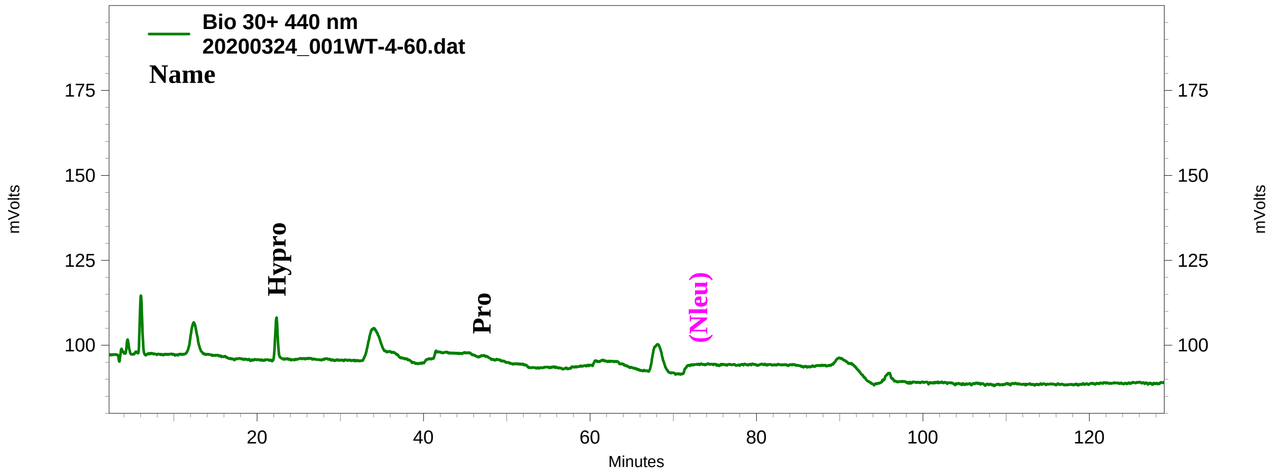


**Bio 30+ 440 nm**
**Results**

| Pk # | Name | Retention Time | Area | ESTD concentration | Units |
|---|---|---|---|---|---|
| 6 | Hypro | 22.333 | 28274936 | 112.857 | µmol/L |
| 12 | Pro | 46.967 | 2793162 | 6.059 | µmol/L |
| | Nleu | | | 0.000 BDL | µmol/L |

| | | | | | |
|---|---|---|---|---|---|
| Totals | | | 31068098 | 118.916 | |
