# Supplementary material for: Pathophysiology of Cerebellar Degeneration in Mitochondrial Disorders: Insights from the Harlequin Mouse
Source: Int J Mol Sci. 2023 Jun 30;24(13):10973. doi: 10.3390/ijms241310973 (PMC10341771; doi:10.3390/ijms241310973)
Supplement: Supplementary file 1 [file ijms-24-10973-s001.zip › Amino acids 6 m cerebellum/20200324_001WT-54_Method Report.pdf]

Biochrom 30+ Final Test

Method: C:\Biochrom\OpenLAB Projects\Default\Method\20180828mod.met
Standard: C:\Biochrom\OpenLAB Projects\Default\Result\20200324_001WT-54.dat
Date : 4/1/2020 9:38:55 AM (GMT +02:00)

Instrument Serial No : 133260
Column No : H-0795
Resin No : 132-56

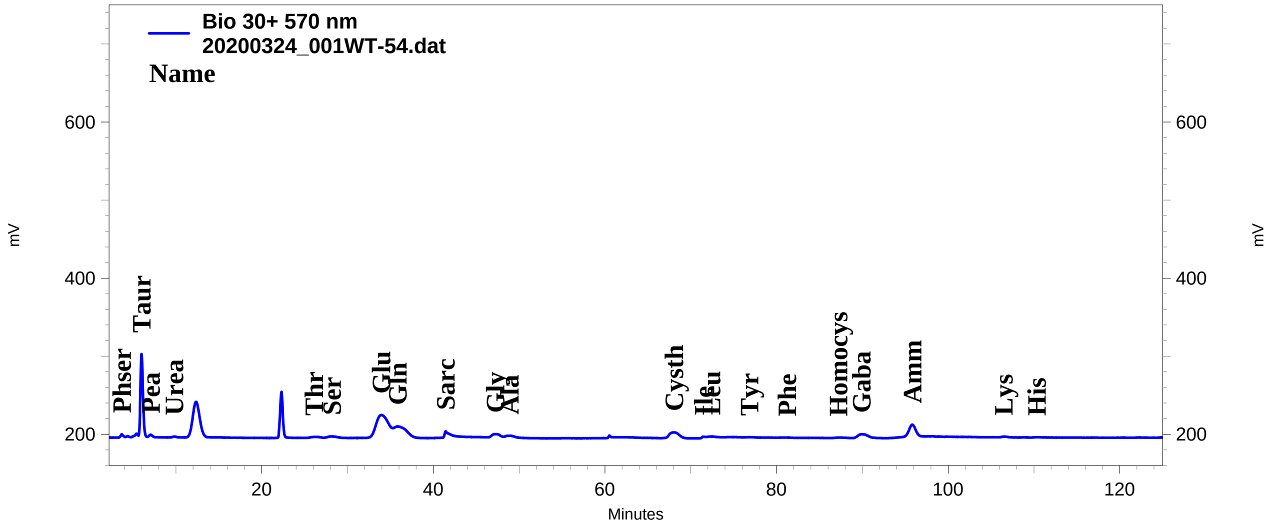

Bio 30+ 570 nm
Results

Pk #	Name	Retention Time	Area	ESTD concentration	Units
1	Phser	3.700	9219585	6.414	µmol/L
4	Taur	6.000	222372607	196.511	µmol/L
5	Pea	7.067	9605235	11.620	µmol/L
6	Urea	9.800	3532126	92.713	µmol/L
	Asp			0.000 BDL	µmol/L
9	Thr	26.100	7647272	5.958	µmol/L
10	Ser	28.167	12916628	9.942	µmol/L
	Asn			0.000 BDL	µmol/L
11	Glu	33.933	294309204	232.894	µmol/L
12	Gln	35.833	151462240	119.613	µmol/L
13	Sarc	41.433	39032585	243.576	µmol/L
	AAAA			0.000 BDL	µmol/L
14	Gly	47.233	27651886	20.088	µmol/L
15	Ala	48.900	18121467	14.328	µmol/L
	Citr			0.000 BDL	µmol/L
	Aaba			0.000 BDL	µmol/L
	Val			0.000 BDL	µmol/L
	Cys			0.000 BDL	µmol/L
	Met			0.000 BDL	µmol/L
17	Cysth	68.067	56510588	40.911	µmol/L
18	Ile	71.500	4277643	3.388	µmol/L
19	Leu	72.400	8591930	6.434	µmol/L
	Nleu			0.000 BDL	µmol/L
20	Tyr	76.833	2341729	1.870	µmol/L
	B-ala			0.000 BDL	µmol/L
21	Phe	81.233	2045676	1.604	µmol/L
	Baiba			0.000 BDL	µmol/L
22	Homocys	87.200	4828667	1.931	µmol/L
23	Gaba	89.900	37982800	38.077	µmol/L
	Ethan			0.000 BDL	µmol/L
24	Amm	95.833	88997170	65.910	µmol/L
	Hylys			0.000 BDL	µmol/L
	Orn			0.000 BDL	µmol/L
25	Lys	106.467	4060594	2.996	µmol/L
	1-Mhis			0.000 BDL	µmol/L
26	His	110.333	2216441	1.567	µmol/L
	Trp			0.000 BDL	µmol/L
	3-Mhis			0.000 BDL	µmol/L
	Ans			0.000 BDL	µmol/L
	Car			0.000 BDL	µmol/L
27	Arg	125.300	5404282	4.366	µmol/L
Totals			1013128355	1122.709	

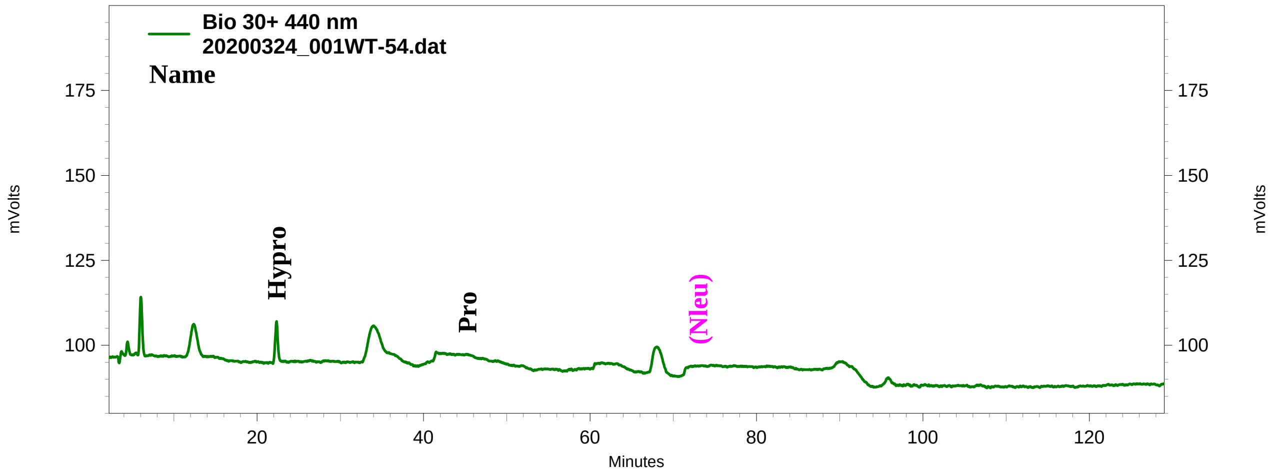

Bio 30+ 440 nm
Results

Pk #	Name	Retention Time	Area	ESTD concentration	Units
6	Hypro	22.333	27281176	108.890	µmol/L
11	Pro	45.267	0	0.000	µmol/L
	Nleu			0.000 BDL	µmol/L

Totals					
			27281176	108.890	
