# Supplementary material for: Pathophysiology of Cerebellar Degeneration in Mitochondrial Disorders: Insights from the Harlequin Mouse
Source: Int J Mol Sci. 2023 Jun 30;24(13):10973. doi: 10.3390/ijms241310973 (PMC10341771; doi:10.3390/ijms241310973)
Supplement: Supplementary file 1 [file ijms-24-10973-s001.zip › Supplementary Figure 1..pdf]

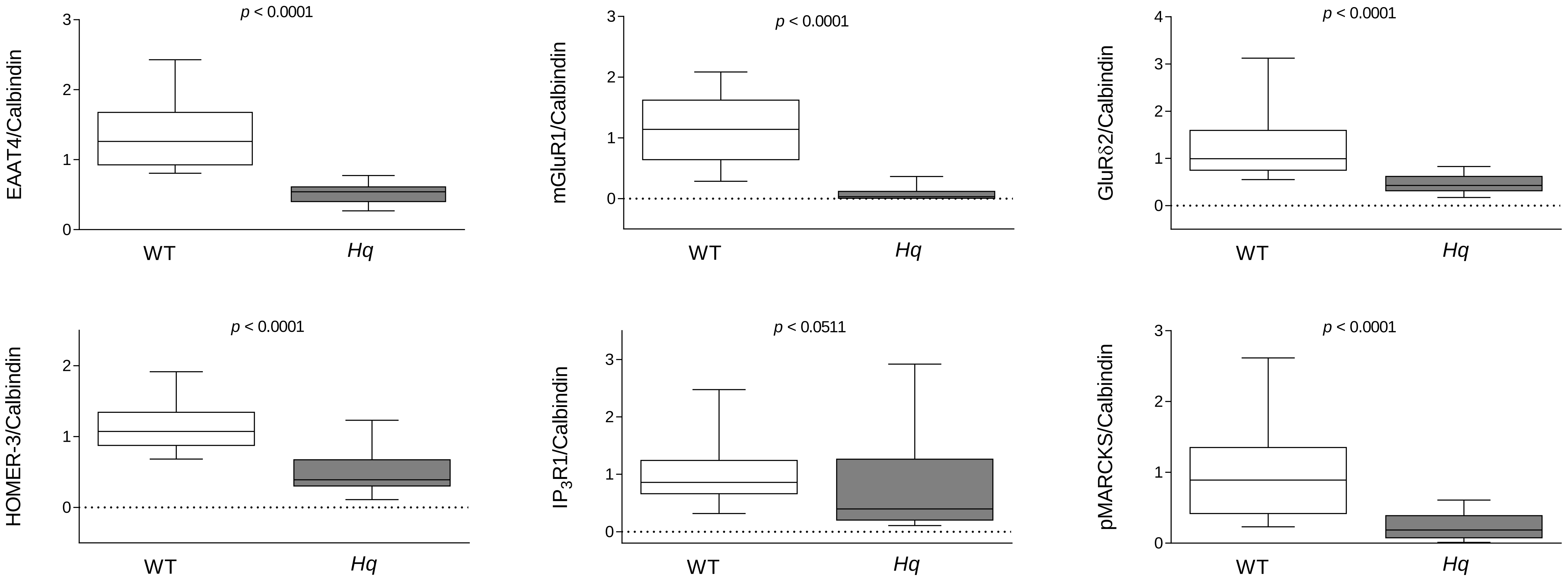

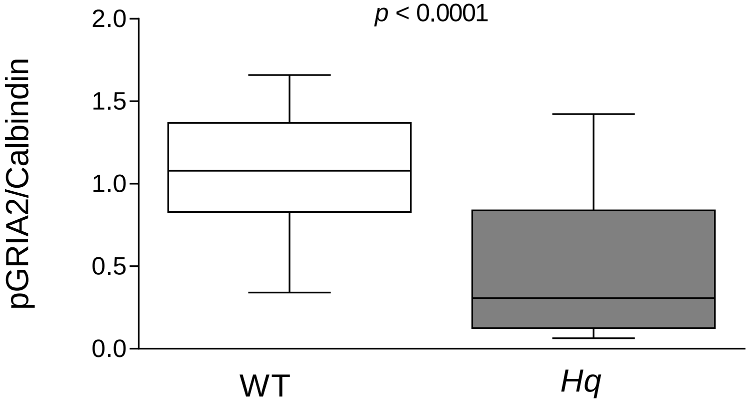

Supplementary Figure 1. Purkinje cell-related proteins corrected by calbindin levels in 5.5-month-old wild type (WT, n= 11-21) and *Harlequin* (*Hq,* n=10-20) mice. Data (mean, interquartile range, and min and max values) are expressed relative to the WT group. *P*-values for differences between WT and *Hq* groups (Mann-Whitney U test) are shown above the graph.
