# Supplementary material for: Pathophysiology of Cerebellar Degeneration in Mitochondrial Disorders: Insights from the Harlequin Mouse
Source: Int J Mol Sci. 2023 Jun 30;24(13):10973. doi: 10.3390/ijms241310973 (PMC10341771; doi:10.3390/ijms241310973)
Supplement: Supplementary file 1 [file ijms-24-10973-s001.zip › Supplementary Figure 2.pdf]

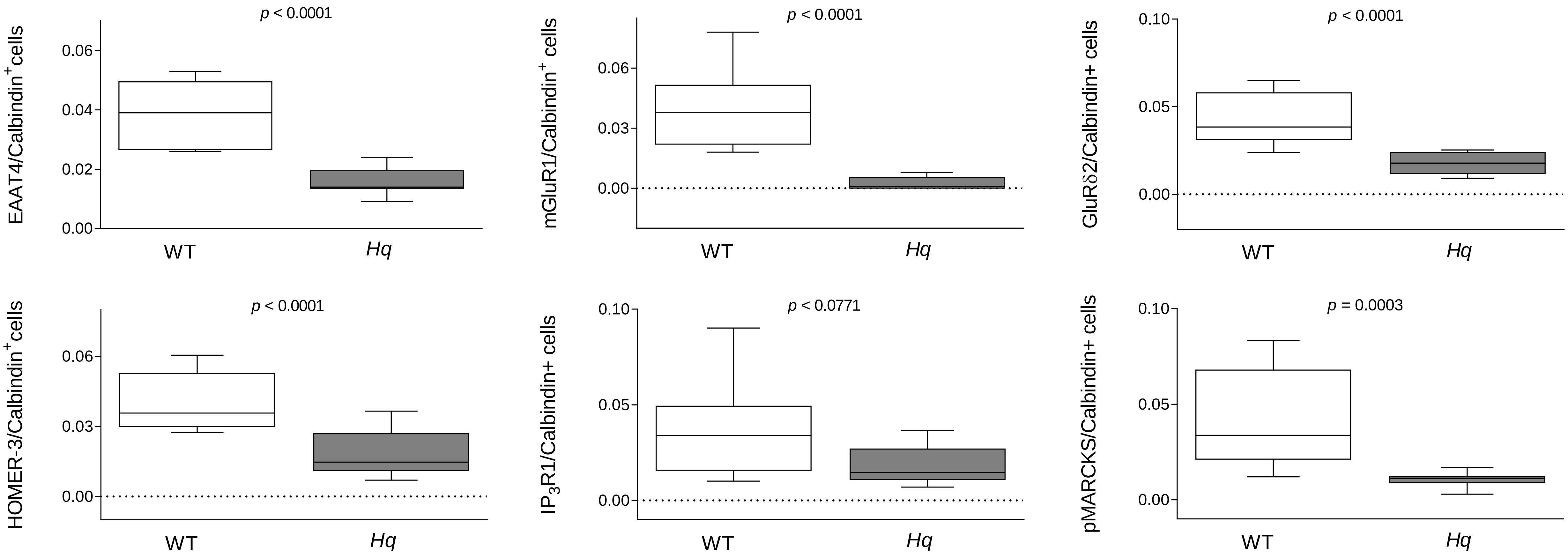

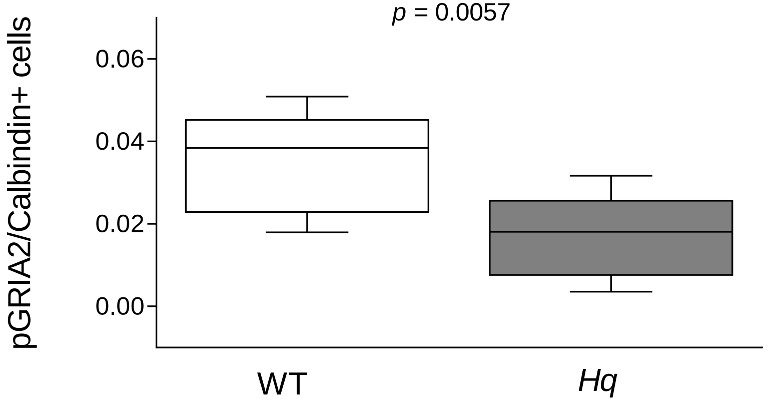

Supplementary Figure 2. Purkinje cell-related proteins corrected by the number of calbindin positive cells measured in sagittal sections of cerebella of 5.5-month-old wild type (WT, n= 8-10) and Harlequin (*Hq,* n=9-10) mice. Data (mean, interquartile range, and min and max values) are expressed relative to the WT group. *P*-values for differences between WT and *Hq* groups (Mann-Whitney U test) are shown above the graph.
