# Supplementary material for: Pathophysiology of Cerebellar Degeneration in Mitochondrial Disorders: Insights from the Harlequin Mouse
Source: Int J Mol Sci. 2023 Jun 30;24(13):10973. doi: 10.3390/ijms241310973 (PMC10341771; doi:10.3390/ijms241310973)
Supplement: Supplementary file 1 [file ijms-24-10973-s001.zip › Supplementary Figure 3.pdf]

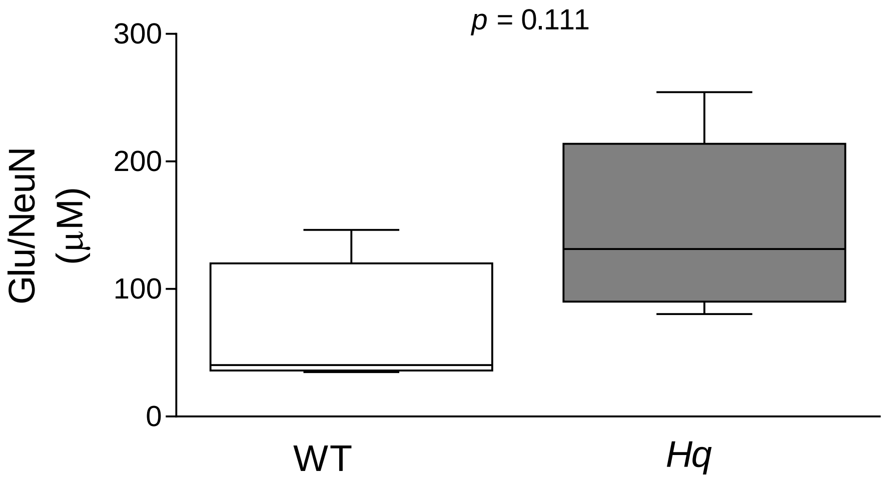

Supplementary Figure 3. Glutamate levels in wild type (WT, n=4) and *Harlequin* (*Hq* n= 5) mice cerebellum at 6 months of age normalized by the granular cell marker NeuN (previously corrected by γ-tubulin, adimensional value). Data are shown as mean, interquartile range, and min and max values. *P*-value for differences between WT and *Hq* groups (Mann-Whitney U test) is shown above the graph.
