# Supplementary material for: Pathophysiology of Cerebellar Degeneration in Mitochondrial Disorders: Insights from the Harlequin Mouse
Source: Int J Mol Sci. 2023 Jun 30;24(13):10973. doi: 10.3390/ijms241310973 (PMC10341771; doi:10.3390/ijms241310973)
Supplement: Supplementary file 1 [file ijms-24-10973-s001.zip › Table S1 Primary Antibodies.pdf]

Supplementary Table 1.Primary Antibodies.

Primary antibody	Reference	Dilution WB	Dilution IMF
AQP4	Sigma HPA014784	1:1000	
Calbindin	Abcam ab11426	1:1000	
EAAT2 (Slc1a2)	Sigma SAB2102171	1:1000	
EAAT4 (Slc1a6)	Abcam ab41650	1:1000	1.100
GAT-3	Abcam ab122430	1:1000	
GFAP	Sigma G3893		1:500
GluRδ2	Abcam ab190358	1:2000	
GRIA2 (Ser880)	Abcam ab52180	1:1000	
HOMER-3	Abcam ab175852	1:1000	
IP3R	Abcam ab5804	1:1000	
MARCKS (Ser152/156)	Merck #07-1238	1:1000	
NeuN	Abcam ab177487	1:1000	
γ-tubulin	Sigma T6557	1:5000	
