# Supplementary material for: Pathophysiology of Cerebellar Degeneration in Mitochondrial Disorders: Insights from the Harlequin Mouse
Source: Int J Mol Sci. 2023 Jun 30;24(13):10973. doi: 10.3390/ijms241310973 (PMC10341771; doi:10.3390/ijms241310973)
Supplement: Supplementary file 1 [file ijms-24-10973-s001.zip › Western blot images.pptx]

## Slide 1
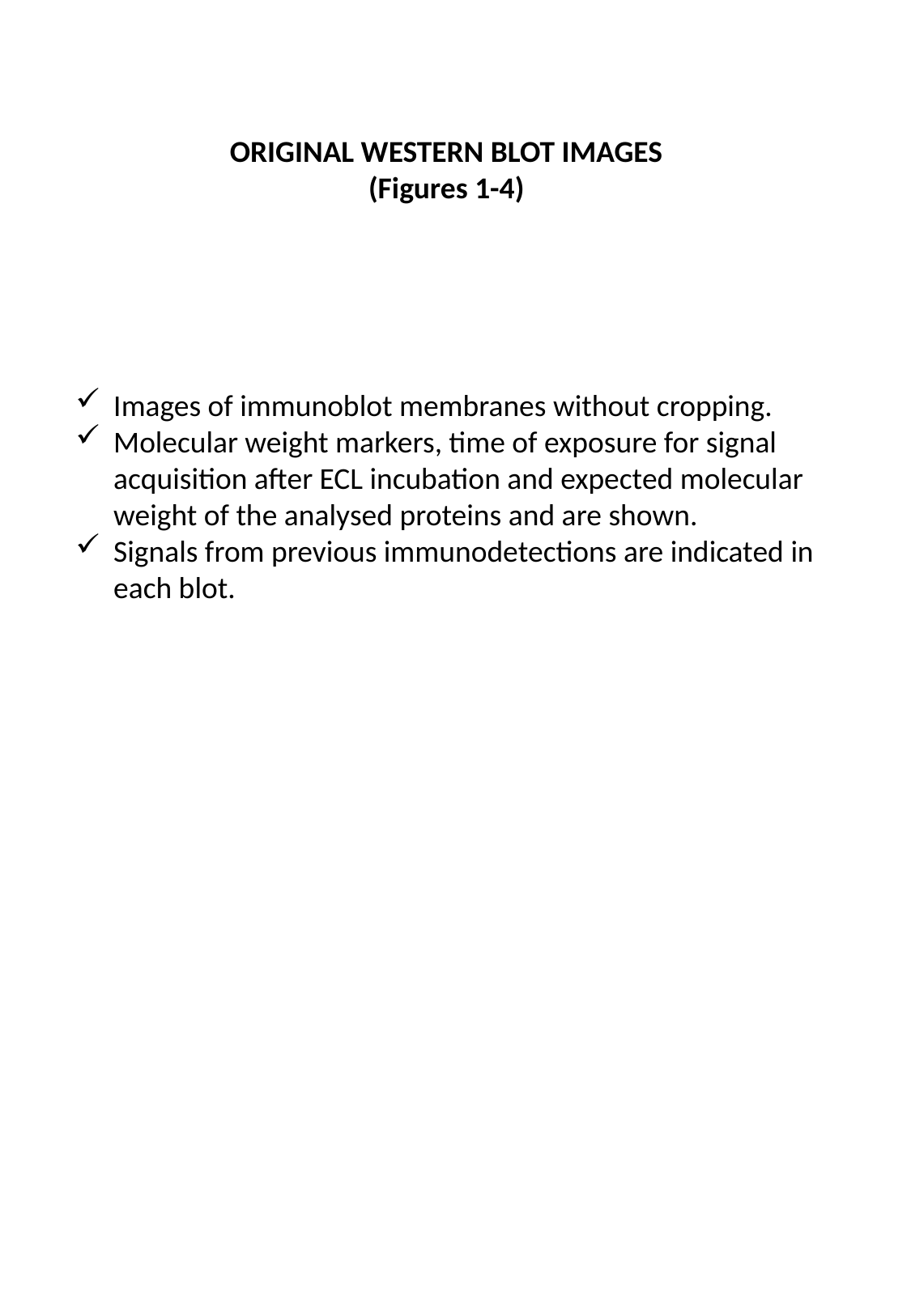

ORIGINAL WESTERN BLOT IMAGES
(Figures 1-4)
Images of immunoblot membranes without cropping.
Molecular weight markers, time of exposure for signal acquisition after ECL incubation and expected molecular weight of the analysed proteins and are shown.
Signals from previous immunodetections are indicated in each blot.

## Slide 2
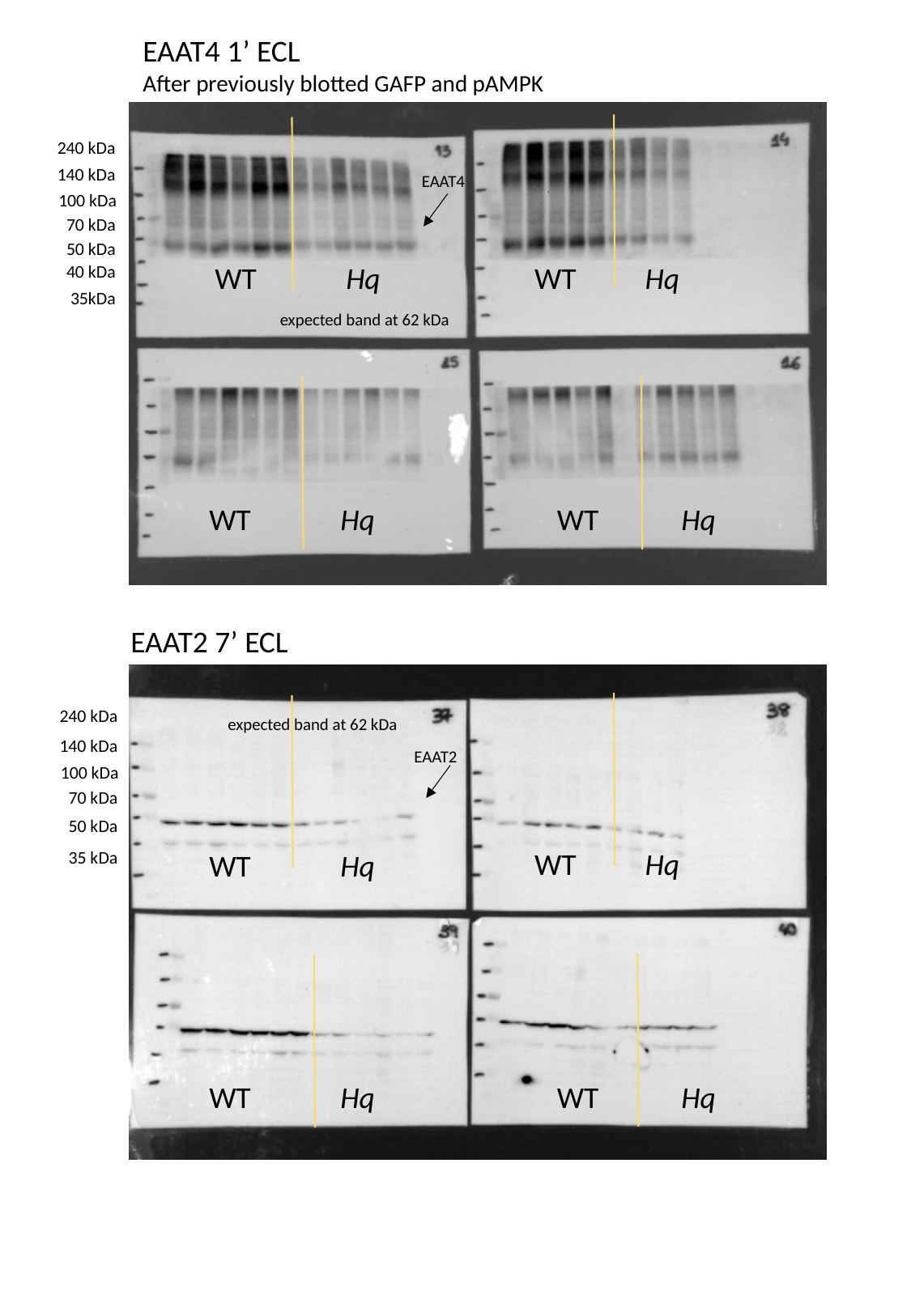

EAAT4 1’ ECL
After previously blotted GAFP and pAMPK
240 kDa
140 kDa
100 kDa
70 kDa
50 kDa
40 kDa
35kDa
EAAT4
WT Hq
WT Hq
expected band at 62 kDa
WT Hq
WT Hq
EAAT2 7’ ECL
240 kDa
140 kDa
100 kDa
70 kDa
50 kDa
35 kDa
expected band at 62 kDa
EAAT2
WT Hq
WT Hq
WT Hq
WT Hq

## Slide 3
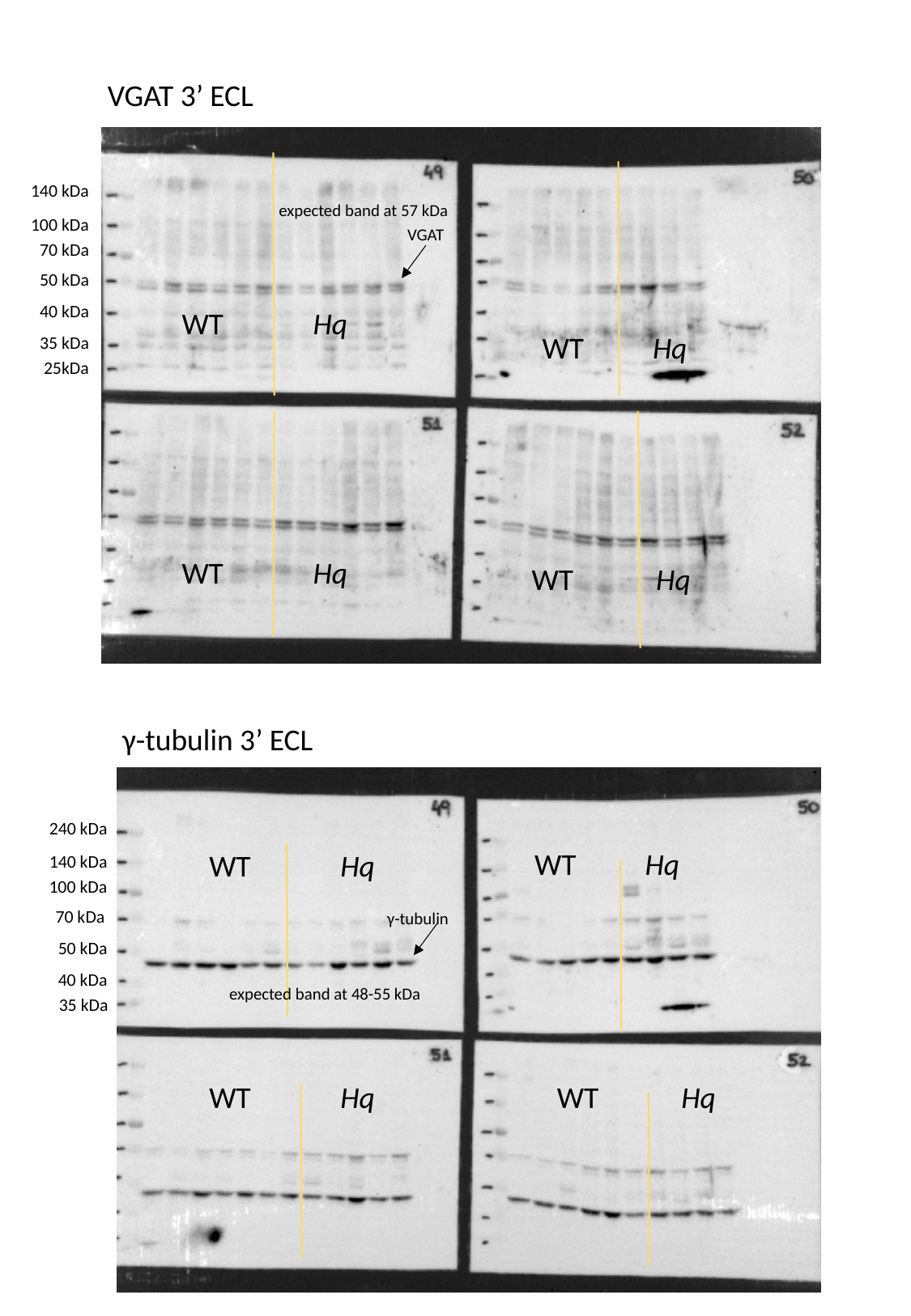

VGAT 3’ ECL
140 kDa
100 kDa
70 kDa
50 kDa
40 kDa
35 kDa
25kDa
expected band at 57 kDa
VGAT
WT Hq
WT Hq
WT Hq
WT Hq
γ-tubulin 3’ ECL
240 kDa
140 kDa
100 kDa
70 kDa
50 kDa
40 kDa
35 kDa
WT Hq
WT Hq
γ-tubulin
expected band at 48-55 kDa
WT Hq
WT Hq

## Slide 4
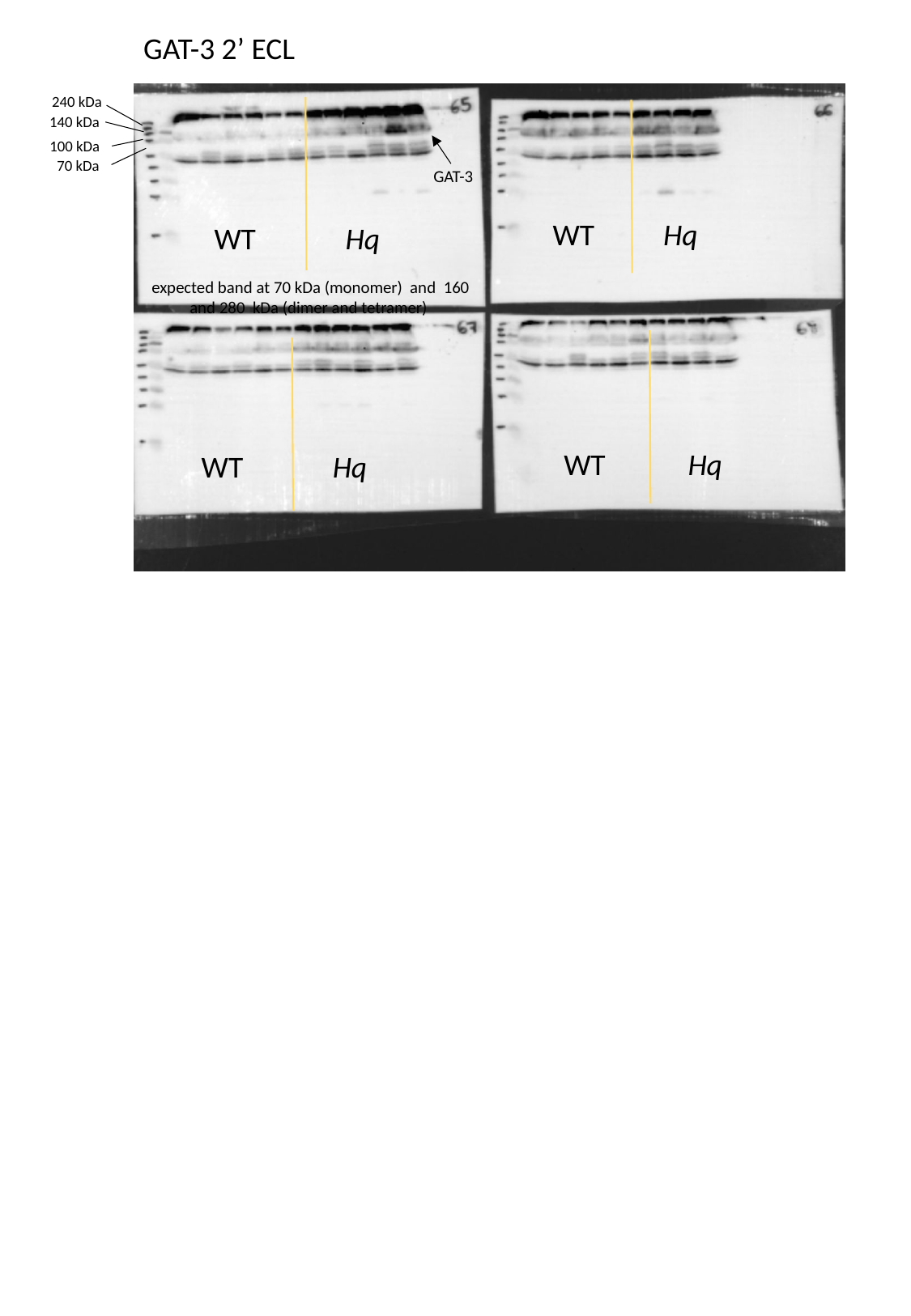

GAT-3 2’ ECL
240 kDa
140 kDa
100 kDa
70 kDa
GAT-3
WT Hq
WT Hq
expected band at 70 kDa (monomer) and 160 and 280 kDa (dimer and tetramer)
WT Hq
WT Hq

## Slide 5
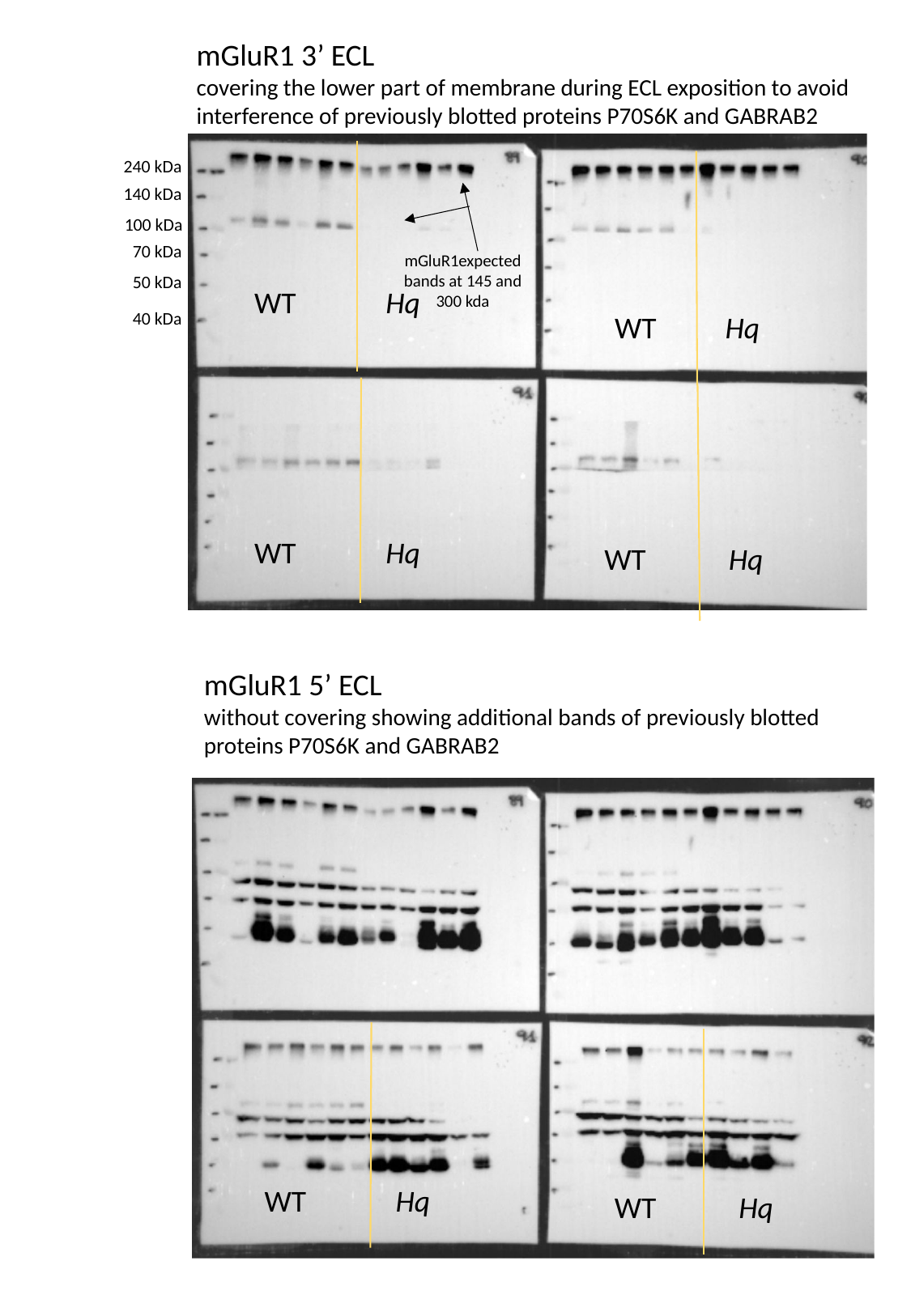

mGluR1 3’ ECL
covering the lower part of membrane during ECL exposition to avoid interference of previously blotted proteins P70S6K and GABRAB2
240 kDa
140 kDa
100 kDa
70 kDa
50 kDa
40 kDa
mGluR1expected bands at 145 and 300 kda
WT Hq
WT Hq
WT Hq
WT Hq
mGluR1 5’ ECL
without covering showing additional bands of previously blotted proteins P70S6K and GABRAB2
WT Hq
WT Hq

## Slide 6
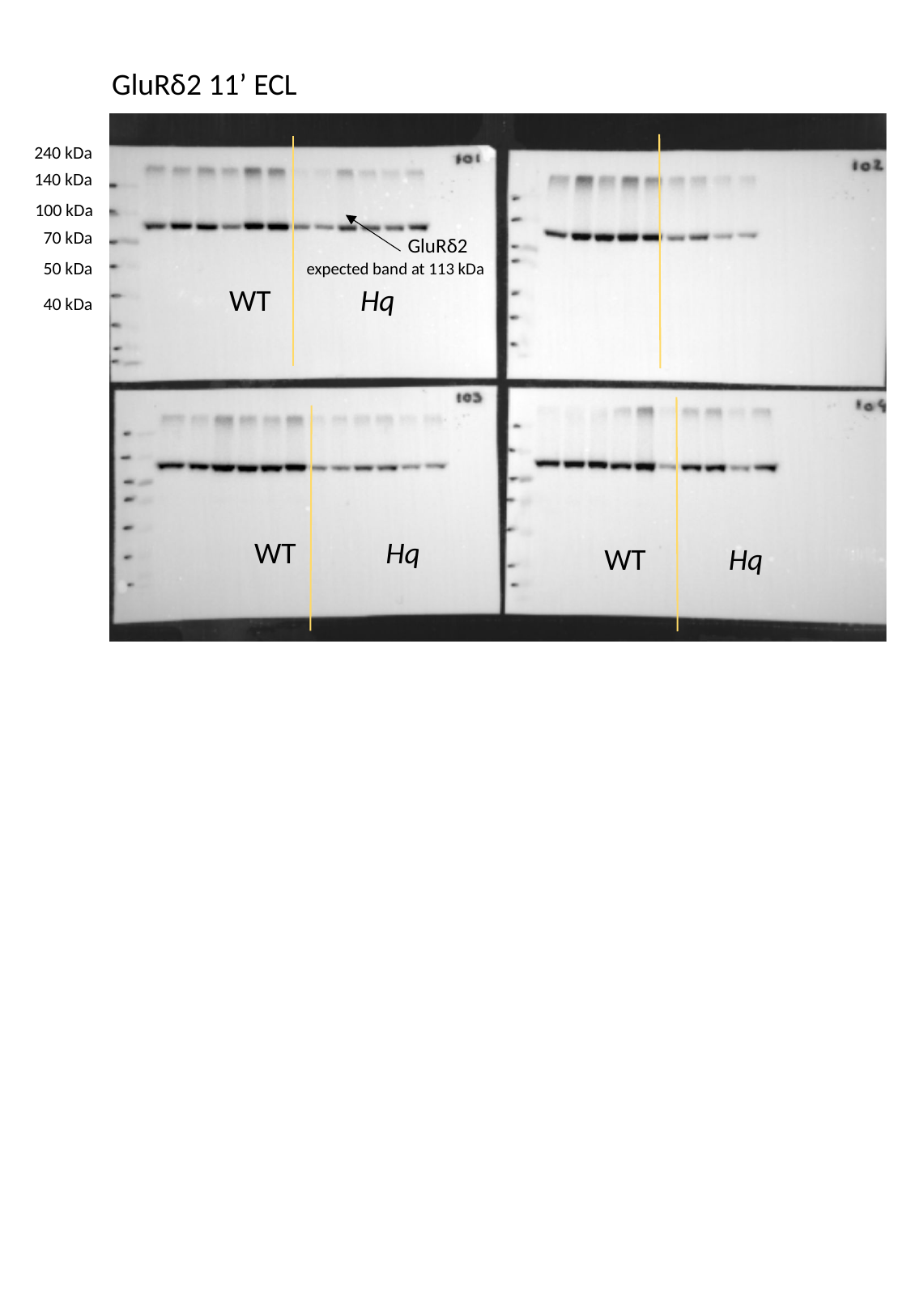

GluRδ2 11’ ECL
240 kDa
140 kDa
100 kDa
70 kDa
50 kDa
40 kDa
GluRδ2
expected band at 113 kDa
WT Hq
WT Hq
WT Hq

## Slide 7
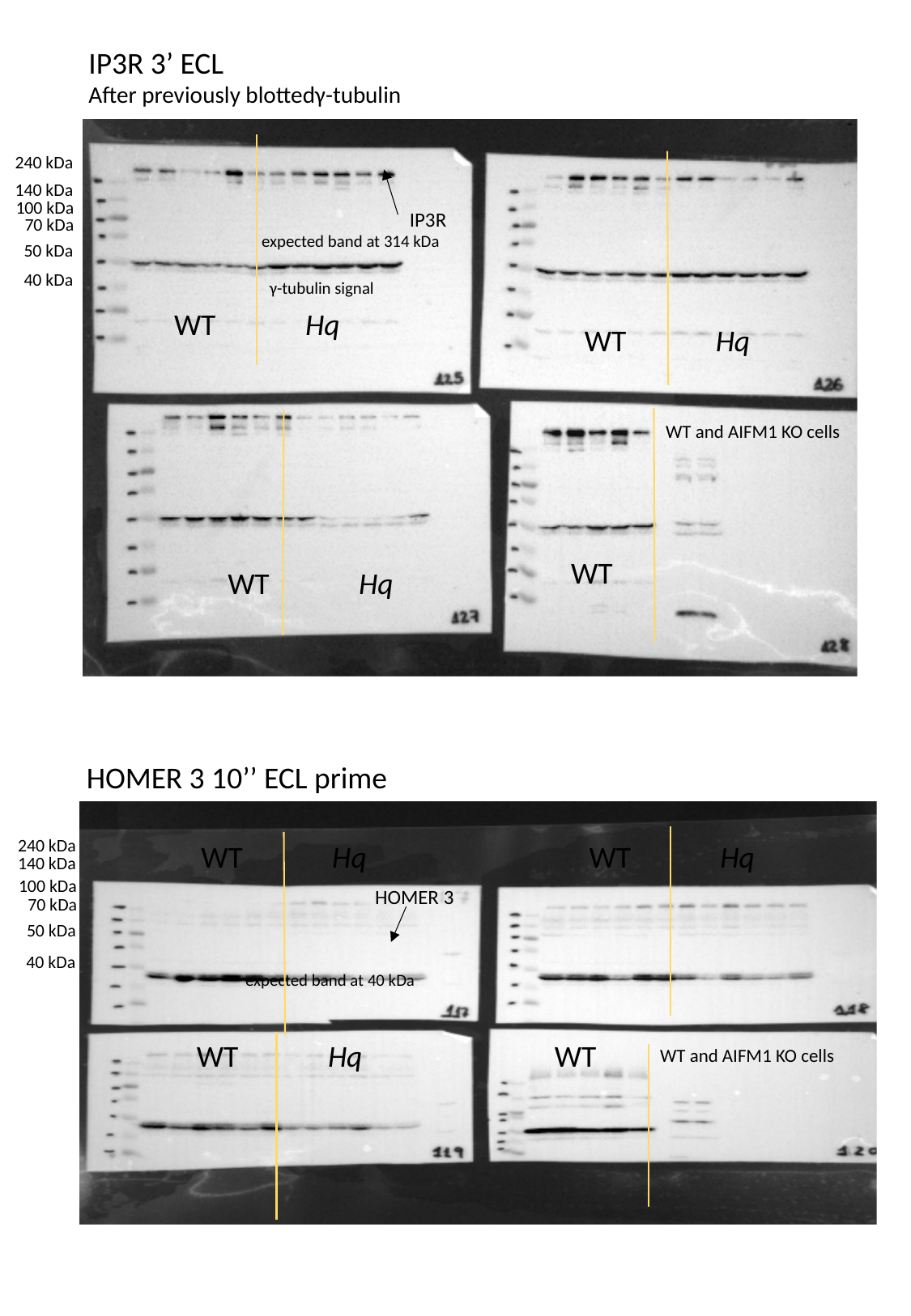

IP3R 3’ ECL
After previously blottedγ-tubulin
240 kDa
140 kDa
100 kDa
70 kDa
50 kDa
40 kDa
IP3R
expected band at 314 kDa
γ-tubulin signal
WT Hq
WT Hq
WT and AIFM1 KO cells
WT
WT Hq
HOMER 3 10’’ ECL prime
240 kDa
140 kDa
100 kDa
70 kDa
50 kDa
40 kDa
WT Hq
WT Hq
HOMER 3
expected band at 40 kDa
WT Hq
WT
WT and AIFM1 KO cells

## Slide 8
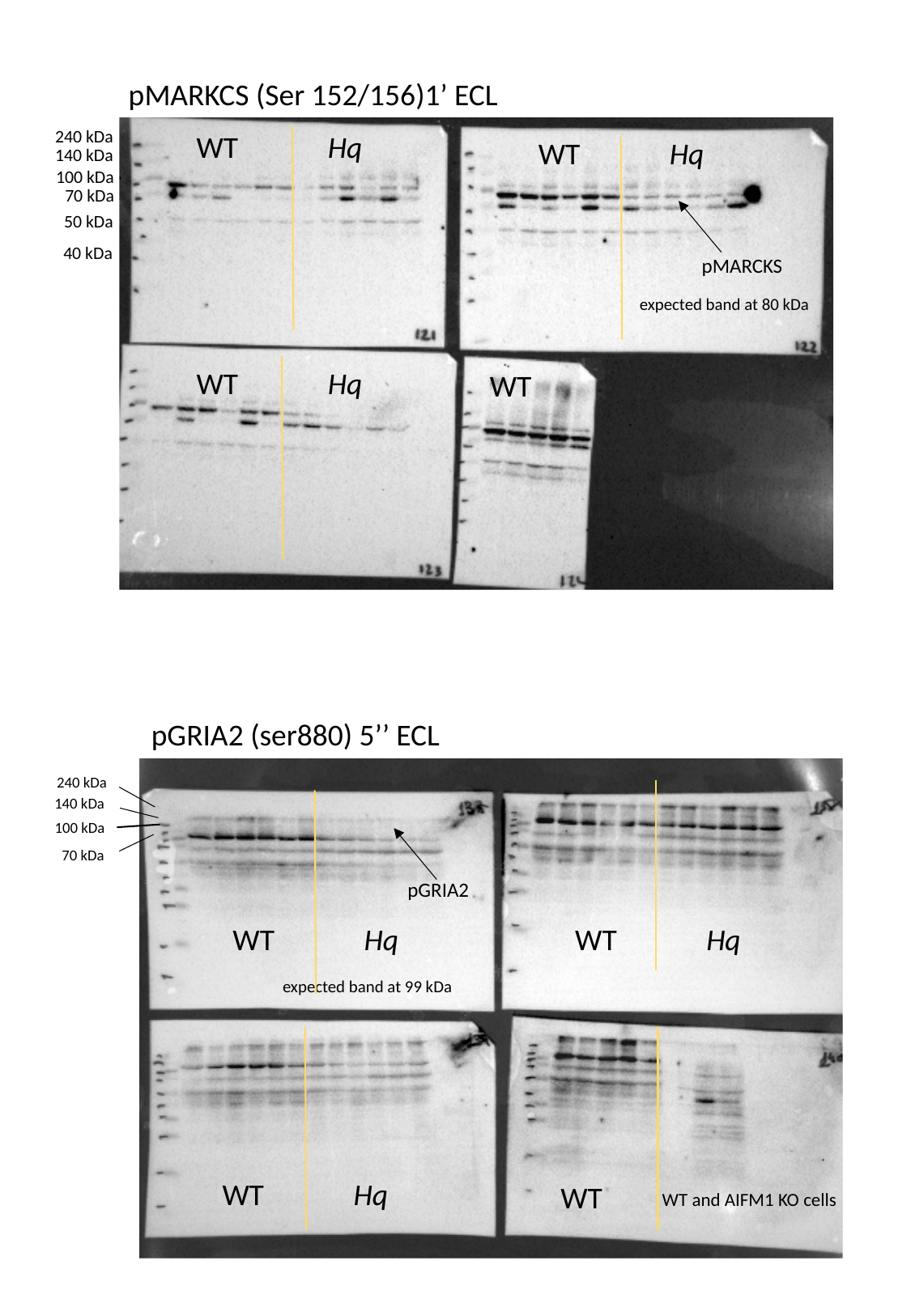

pMARKCS (Ser 152/156)1’ ECL
240 kDa
140 kDa
100 kDa
70 kDa
50 kDa
40 kDa
WT Hq
WT Hq
pMARCKS
expected band at 80 kDa
WT Hq
WT
pGRIA2 (ser880) 5’’ ECL
240 kDa
140 kDa
100 kDa
70 kDa
pGRIA2
WT Hq
WT Hq
expected band at 99 kDa
WT Hq
WT
WT and AIFM1 KO cells

## Slide 9
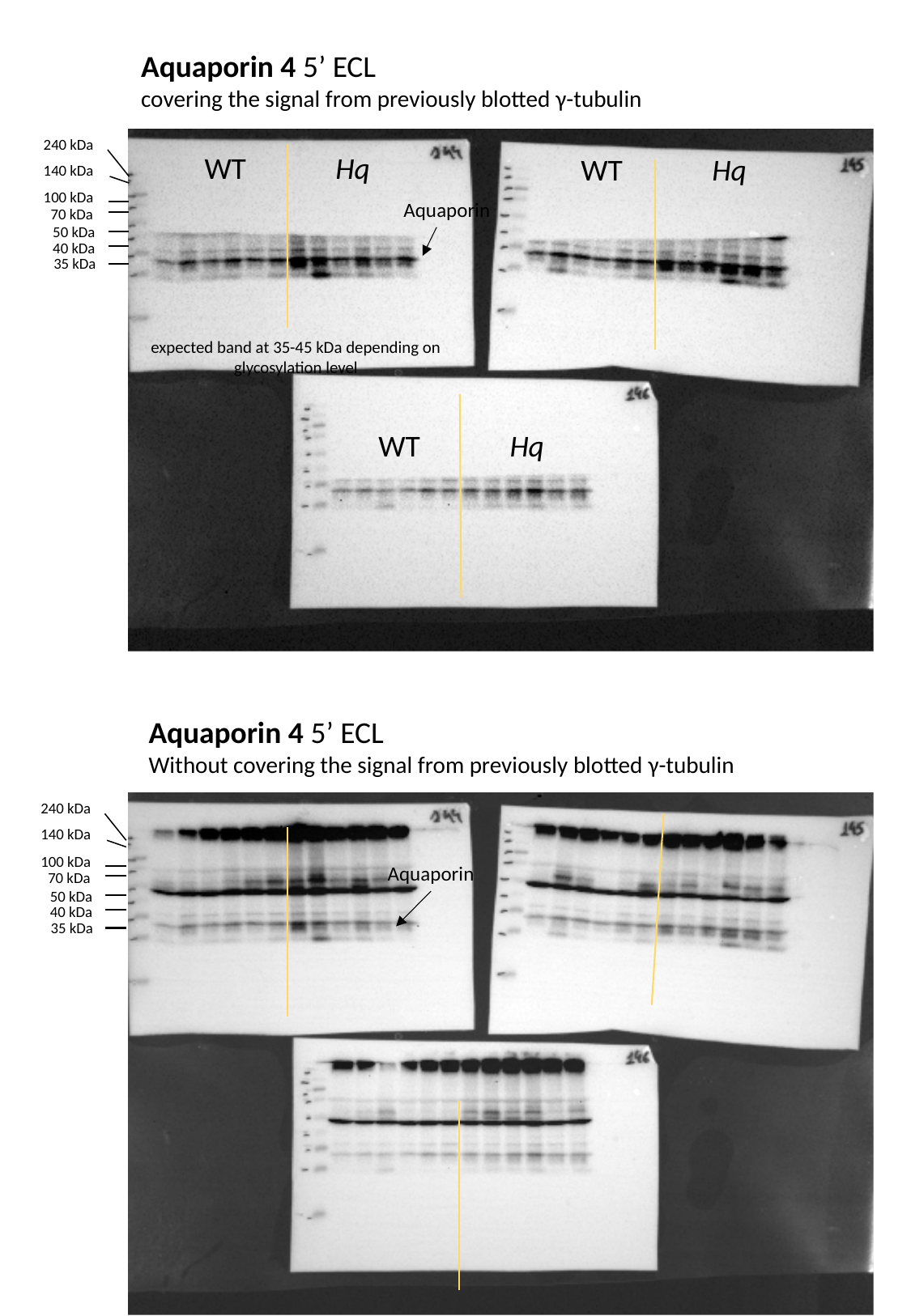

Aquaporin 4 5’ ECL
covering the signal from previously blotted γ-tubulin
240 kDa
140 kDa
100 kDa
70 kDa
50 kDa
40 kDa
35 kDa
WT Hq
WT Hq
Aquaporin
expected band at 35-45 kDa depending on glycosylation level
WT Hq
Aquaporin 4 5’ ECL
Without covering the signal from previously blotted γ-tubulin
240 kDa
140 kDa
100 kDa
70 kDa
50 kDa
40 kDa
35 kDa
Aquaporin
